# Probing GFP Chromophore Analogs as Anti-HIV Agents Targeting LTR-III G-Quadruplex

**DOI:** 10.3390/biom11101409

**Published:** 2021-09-26

**Authors:** Dmitriy Y. Ryazantsev, Mikhail Yu. Myshkin, Vera A. Alferova, Vladimir B. Tsvetkov, Elena Y. Shustova, Polina N. Kamzeeva, Polina V. Kovalets, Elvira R. Zaitseva, Nadezhda S. Baleeva, Timofei S. Zatsepin, Zakhar O. Shenkarev, Mikhail S. Baranov, Liubov I. Kozlovskaya, Andrey V. Aralov

**Affiliations:** 1 Shemyakin-Ovchinnikov Institute of Bioorganic Chemistry, Russian Academy of Sciences, 117997 Moscow, Russia; d.yu.ryazantsev@gmail.com (D.Y.R.); mikhail.myshkin@phystech.edu (M.Y.M.); alferovava@gmail.com (V.A.A.); polinabast@yandex.ru (P.N.K.); kovalecarz@yandex.ru (P.V.K.); elvira19999@mail.ru (E.R.Z.); nsbaleeva@gmail.com (N.S.B.); zakhar-shenkarev@yandex.ru (Z.O.S.); baranovmikes@gmail.com (M.S.B.); 2 Gause Institute of New Antibiotics, B. Pirogovskaya 11, 119021 Moscow, Russia; 3 World-Class Research Center “Digital Biodesign and Personalized Healthcare”, Sechenov First Moscow State Medical University, 8/2 Trubetskaya Str., 119146 Moscow, Russia; v.b.tsvetkov@gmail.com; 4 Federal Research and Clinical Center of Physical-Chemical Medicine of Federal Medical Biological Agency, 119435 Moscow, Russia; 5 Chumakov Federal Scientific Center for Research and Development of Immune-and-Biological Products of Russian Academy of Sciences, 108819 Moscow, Russia; Riw.sun@list.ru; 6 D. Mendeleev University of Chemical Technology of Russia, 9 Miusskaya Sq., 125047 Moscow, Russia; 7 Pirogov Russian National Research Medical University, Ostrovitianov 1, 117997 Moscow, Russia; 8 Center for Life Sciences, Skolkovo Institute of Science and Technology, 121205 Moscow, Russia; t.zatsepin@skoltech.ru; 9 Sechenov First Moscow State Medical University, 119991 Moscow, Russia; 10 G4_Interact, USERN, University of Pavia, 27100 Pavia, Italy

**Keywords:** G-quadruplex, FRET-melting, green fluorescent protein (GFP) chromophore, antiviral activity, cytotoxicity

## Abstract

Green fluorescent protein (GFP) chromophore and its congeners draw significant attention mostly for bioimaging purposes. In this work we probed these compounds as antiviral agents. We have chosen LTR-III DNA G4, the major G-quadruplex (G4) present in the long terminal repeat (LTR) promoter region of the human immunodeficiency virus-1 (HIV-1), as the target for primary screening and designing antiviral drug candidates. The stabilization of this G4 was previously shown to suppress viral gene expression and replication. FRET-based high-throughput screening (HTS) of 449 GFP chromophore-like compounds revealed a number of hits, sharing some general structural features. Structure-activity relationships (SAR) for the most effective stabilizers allowed us to establish structural fragments, important for G4 binding. Synthetic compounds, developed on the basis of SAR analysis, exhibited high LTR-III G4 stabilization level. NMR spectroscopy and molecular modeling revealed the possible formation of LTR-III G4-ligand complex with one of the lead selective derivative ZS260.1 positioned within the cavity, thus supporting the LTR-III G4 attractiveness for drug targeting. Selected compounds showed moderate activity against HIV-I (EC50 1.78–7.7 μM) in vitro, but the activity was accompanied by pronounced cytotoxicity.

## 1. Introduction

**Figure 1:**
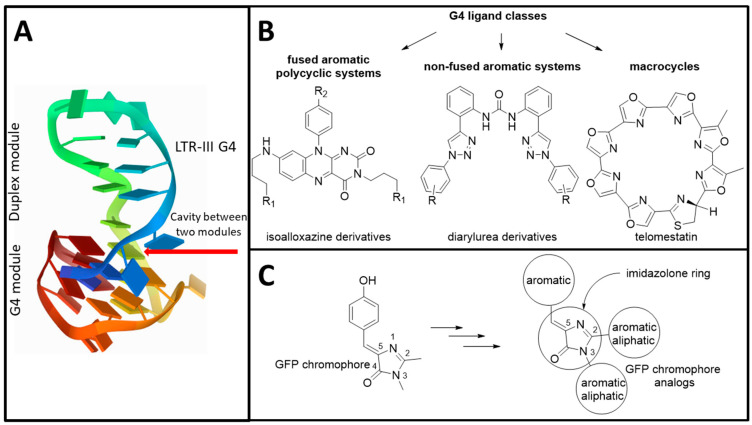
G4 target studied and the principles of ligand design: (**A**) HIV-1 LTR-III G4 structure (PDB 6H1K); (**B**) major types of G4-binding compounds; (**C**) the structure of GFP chromophore and its synthetic derivatization strategy.

G-quadruplexes (G4s) are alternative secondary structures formed by guanine-rich nucleic acids. In quadruplex-folded polynucleotides, four guanine bases of the same or different strands form a planar structure stabilized by Hoogsteen hydrogen bonds [1]. G4s are currently considered as promising and attractive targets for anti-cancer [2], anti-viral [3], and antibacterial therapy [4]. The sites capable of forming RNA G-quadruplexes have been identified in the genomes of human immunodeficiency virus 1 (HIV-1), Zika virus, Ebola Virus, papillomaviruses, etc., [3,5,6]. Recently a number of G4s in the HIV-1 DNA have been extensively studied [7,8,9,10]. Among the most attractive targets for repressing virus production are G4s formed in the 5′-long terminal repeat (LTR) promoter, which is crucial for effective progression of the viral cycle [11]. Among these G4s, the LTR-III G4 is considered to be major G4 structure with the highest thermal stability and has an unusual duplex-quadruplex junction that can be potentially specifically targeted (Figure 1A) [12]. Therefore, in this work we screened ligands for HIV-1 LTR-III G4 as potential anti-HIV agents.

G4 ligands are low molecular weight compounds that can bind to G4s and modulate their thermal stability. This interaction can lead to the disregulation of replication, translation, or transcription, and therefore may be used for drug design [13,14,15,16]. There are three major structural types of G4-interacting compounds: (1) fused aromatic polycyclic systems; (2) non-fused aromatic systems, in which the aromatic fragments are connected by short linkers; and (3) macrocyclic compounds (Figure 1B) [1]. Recently, benzylamine-like motifs have been successively used to selectively bind with high affinity to quadruplex–duplex junctions of LTR-III G4, and they could be attributed to the class of the fused aromatic polycyclic systems [17].

GFP chromophore and its synthetic analogues find diverse applications in bioimaging and fluorescent probes design [18,19,20,21]. Interestingly, the structural features of these compounds (Figure 1C) resemble, to a significant extent, the second type of G4-binding compounds, the non-fused aromatic systems (Figure 1B). Therefore, we decided to probe a number of GFP chromophore derivatives as G4 ligands and antiviral agents.

## 2. Materials and Methods

### 2.1. Fret Melting Assay

To evaluate stabilizing properties of the ligands in a complex with oligodeoxyribonucleotides (ODNs) melting with fluorescence registration was used. ODN (0.5 μM) (Table 1), labeled with 6-carboxyfluorescein (FAM) and 6-carboxytetramethylrhodamine (TAMRA) at the 5′- and 3′-end, respectively, were folded in lithium cacodylate buffer (10 mM, pH 7.4) with 100 mM KCl by heating at 95 °C for 5 min and gradually cooling to r.t. GFP chromophore analogs (5 mM solution in DMSO) were diluted with the same buffer to the final concentration of 25 μM and mixed with an equal volume of 0.5 μM ODN solution resulting in a molar ratio of 50:1 (total volume was 50 μL). For the titration experiments molar ratios of 20:1 and 2:1 were used. Samples were analyzed using DTprime qPCR system (DNA-Technology LLC, Moscow, Russia). Fluorescence was registered by detection of FAM emission at 520 nm upon excitation at 495 nm in the temperature range of 30–95 °C with 1 °C/min gradient. T_m_ was determined using the instrument software and ΔT_m_ was calculated relative to ODN T_m_ in a buffer solution containing 0.25% (*v/v*) DMSO. The primary and secondary screening were carried out in singlicate and triplicate, respectively.

### 2.2. NMR Experiments

All NMR spectra were acquired on a Bruker Avance III 800 MHz spectrometer equipped with a cryoprobe. Titration of 50 μM LTR-III samples (70 mM KCl, 20 mM potassium phosphate buffer, pH 7.0) with 2 mM ZS260.1 and N979 ligands dissolved in DMSO was performed. 1D ^1^H NMR spectra were used to track the changes during titration. ^1^H-^13^C HSQC and HMBC at 35 °C were used to transfer the assignment of H6/C6 and H8/C8 signals from previously obtained assignment [12]. To identify the LTR-III residues that are most influenced by addition of ZS260.1 ligand, the intensities of cross-peaks in the ^1^H-^13^C HSQC spectra measured with and without added ligand were compared. The H6/C6 and H8/C8 cross-peak intensities were measured at molar ratio of LTR-III to ZS260.1 of 5:1 (35 °C, 0.5 mM LTR-III G4).

### 2.3. Molecular Modeling

Preparation of target and ligand models, docking and molecular dynamics were implemented according to the protocols described in the article [22]. The 3D models of the studied structures were built using molecular graphics software package Sybyl-X software (Certara, Inc., St. Louis, MO, USA). Partial charges on ZS260.1 atoms were calculated using MP2, conductor-like polarizable continuum model (CPCM) [23], 6–31 g* basis sets and Merz-Singh-Kollman scheme [24] with RESP (Restrained ElectroStatic Potential) method [25]. All quantum mechanics simulations were carried out using Gaussian 09 program [26]. To define the most probable binding site of ZS260.1 on LTR-III surface, the procedure of flexible ligand docking was performed using ICM-Pro 3.8.6 [27]. MD simulations were performed using Amber 20 software [28]. An influence of the solvent simulated with application model of water molecules OPC3 [29]. The simulation performed using periodical boundary conditions and rectangular box. The buffer between DNA-ligand complex and the periodic box wall was at least 15 Å. For neutralizing of the negative charge of DNA backbone K+ ions were used. The parameters needed for interatomic energy calculation, were taken from the force fields OL15 [30,31] for DNA and from general amber force field (gaff2) for ZS260.1. The MD simulations in production phase were carried out using constant temperature (T = 300 K) and constant pressure (*p* = 1 atm) over 80 ns. To control the temperature Langevin thermostat was used with the collision frequency of 1 ps-1. Energies were estimated by using the MM-GBSA approach. The polar contribution EGB was computed using the Generalized Born (GB) method and the algorithm for calculating the effective Born radii [32]. The non-polar contribution to the solvation energy (Esurf), which includes solute-solvent van der Waals interactions and the free energy of cavity formation in solvent, was estimated from a solvent-accessible surface area (SASA).

### 2.4. Biological Experiments

#### 2.4.1. Cells and Viruses

Human T cell leukemia (MT4) cell line was maintained in RPMI-1640 (“Chumakov FSC R&D IBP RAS”, Moscow, Russia) with 10% of fetal bovine serum (FBS, Invitrogen, Waltham, MA, USA), L-glutamine and gentamicin. Green monkey kidney (Vero, Beach, FL, USA) cell line was maintained in 2 × Eagle MEM (“Chumakov FSC R&D IBP RAS”, Russia) with 5% FBS with L-glutamine, penicillin and streptomycin. Cell cultures were incubated in the present of 5% CO_2_ at 37 °C. HIV-1 strain NL4-3 was obtained via cell transfection with pNL4-3 3 (ARP2006, NIBSC, Potters Bar, UK).

#### 2.4.2. Cytotoxicity Test

Two-fold dilutions of studied compounds and DMSO as a negative control were prepared in RPMI-1640 (“Chumakov FSC R&D IBP RAS”, Russia). MT4 cell suspensions were added to the wells with compound dilutions and DMSO control (approx. 2 × 10^4^ cells per well). The final concentration series of eight dilutions started from 25 μM. After incubation at 37 °C in a CO_2_-incubator for nine days, cells were analyzed by microscopy. Cytotoxic concentration (CC_50_) values (compound concentration required to induce death of 50% of the cells in monolayer) were calculated according to the Karber method [33]. All experimental procedures were performed in two replicates and were repeated two to four times. Mean and SD were calculated with OriginPro 8 (OriginLab, St. Louis, MO, USA).

#### 2.4.3. Cytopathic Effect Inhibition Test

Eight two-fold dilutions of stock solutions of the compounds in two replicates were prepared in RPMI-1640 (“Chumakov FSC R&D IBP RAS”, Russia). Compound dilutions were mixed with equal volumes of the virus suspension containing 100 CCID_50_ (50% tissue culture infectious dose). Control cells were treated with the same sequential concentrations of DMSO as in compound dilutions. Then, the MT4 cell suspension (approx. 2 × 10^4^ cells per well) in RPMI-1640 containing 10% FBS (Invitrogen, South America) was added to experimental mixtures. A final concentration series started from 25 μM. Each experiment contained virus dose titration in the inoculate to assure the acceptable dose-range. After a nine-day incubation (5% CO_2_, at 37 °C), cytopathic effect (CPE) was visually accessed via microscope. EC_50_ values were calculated according to the Karber method [33]. All experimental procedures were performed in two replicates and were repeated two to four times. Mean and SD were calculated with OriginPro 8 (OriginLab, St. Louis, MO, USA).

#### 2.4.4. Cell Staining for Confocal Microscopy

Vero cells were seeded on glass coverslips in 12-well plates (a full monolayer on the third day). After 72 h, the culture medium was removed from the cells. Test compound solutions with final concentration of 10 μM in DMEM were added to the cells and incubated at 37 °C with CO_2_ for 24 h. Then the medium was removed and 5 μM Hoechst 33,342 (Sigma, St. Louis, MO, USA) in DMEM was added to each well and incubated at 5% CO_2_ and 37 °C for 30 min. Cells were washed with PBS, incubated with 3.7% paraformaldehyde in PBS for 15 min at room temperature, and washed with PBS again. The coverslips were placed onto the slides with a drop of 10% Mowiol in 0.1M Tris-HCl pH 8.5. Slides were analyzed using Eclipse TE2000 confocal microscope (Nikon, Tokyo, Japan) with 515/30 (blue) and 590/50 (green) filters sets.

## 3. Results and Discussion

### 3.1. Primary Screeening of GFP Chromophore Analogs

**Figure 2:**
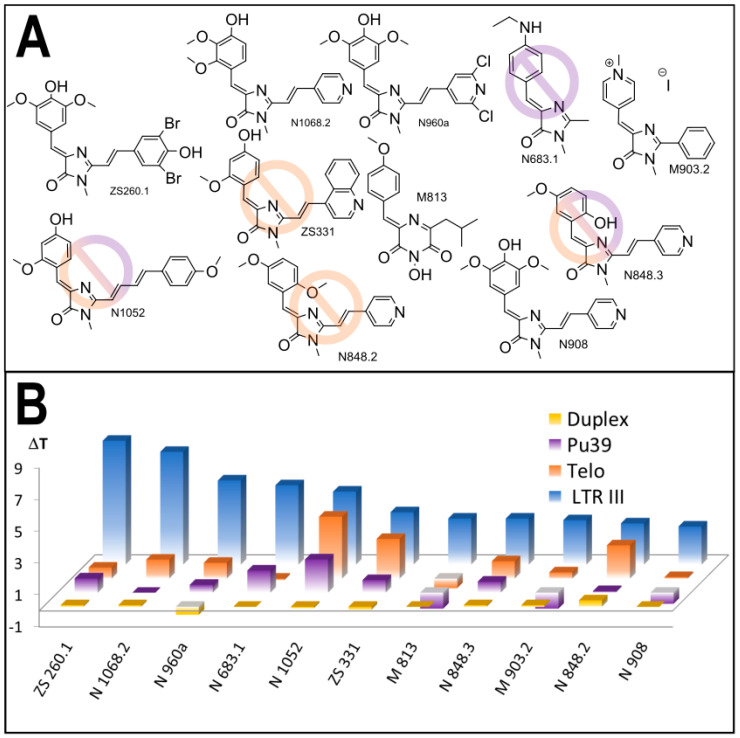
The results of the primary screening: (**A**) the structures of the compounds, selected in primary screening, exhibiting HIV-1 LTR-III G4 stabilization over 2 °C; prohibition sign corresponds to the lack of selectivity; (**B**) comparative stabilization of various sequences by the lead compounds.

Initially we screened an in-house library of 440 synthetic GFP chromophore analogues for interaction with HIV-1 LTR-III sequence, which modulates HIV-1 promoter activity [7,12], using high-throughput screening (HTS) FRET melting assay (Table S1) [34]. The primary probing revealed a number of compounds, capable of HIV-1 LTR-III G4 stabilization. We selected 11 compounds (Figure 2A), displaying stabilization in the range of 2.4–7.8 °C, and evaluated their ability to stabilize other G4-forming sequences, including human telomeric sequence Telo (one of the most common G4 structures in the human genome [35], having two conformations, Hybrid-1 and Hybrid-2 [36]) and model G4 Pu36 (the G-rich Pu39 region of the P1 promoter of the oncogene BCL-2, an apoptosis regulator [37] with polymorphic conformational structure [38]), as well as double stranded oligonucleotide (duplex) control sequence (Figure 2B, Table S2). Some of the selected derivatives showed significant LTR-III selectivity except for N1052, N683.1, ZS331 N848.2 and N848.3.

### 3.2. Synthesis of the Lead Compounds Congeners

The first synthetic efforts for structure optimization included the synthesis of congeners, containing aminoalkyl moiety at three-position of imidazolone ring. This approach was previously successfully utilized for the design of scaffolds for thermal stability enhancement of non-canonical nucleic acids secondary structures [39,40]. Variations, containing protected or protonated aminoalkyl tether (AR556, AR558, AR559, Table S3) were obtained on the basis of the most effective compound ZS260.1. Unfortunately, this modification led to complete loss of stabilization properties (Table S3), suggesting that ionic interactions do not make a significant contribution to or impair the stabilizing properties for this ligand class.

**Figure 3:**
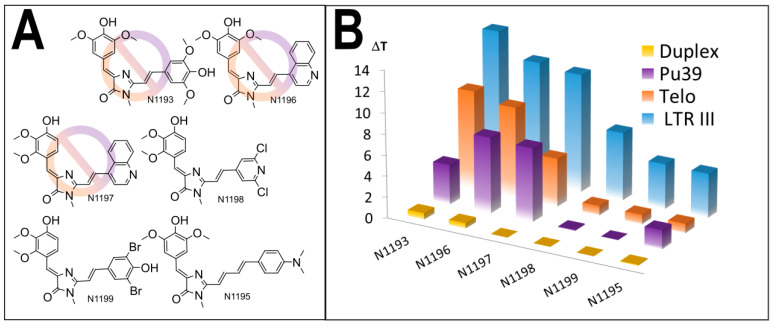
The designed GFP-chromophore-based LTR-III G4 ligands: (**A**) the structures of the compounds, synthesized on the basis of the SAR findings; prohibition sign corresponds to the lack of selectivity; (**B**) comparative stabilization of various DNA structures by the synthesized compounds.

The analysis of structure-activity relationships among the selected compounds, as well as the comparison with the tested structures, containing the same fragments, but exhibiting low or none stabilization capability (Supplement S4), revealed the fragments, beneficial for LTR-III G4 stabilization. For the substituents at five-position of the GFP chromophore core (Figure 1) 2,3- or 3,5-dimethoxy-4-hydroxyaryl moieties (e.g., in compounds ZS260.1, N960a, N908, N1068) were found to be the most suitable for G4-stabilization (Supplement S4-1). In turn, pyridine or dibromophenyl moieties at two-position led to the enhanced stabilizing ability (Supplement S4-2, compounds N1068, N848.3, N848.2, N908, ZS260.1). We designed six additional structures on the basis of these findings (Figure 3A), synthesized them and evaluated their interaction with the same set of G4-forming sequences and double stranded control (Figure 3B, Tables S3 and S5).

Interestingly, all of the compounds exhibited high stabilization of LTR-III G4, the most effective compounds having stabilization range 11–14 °C (N1193, N1196, N1197), significantly exceeding maximal values for the best primary selected compounds (7.8 °C for ZS260.1). These results corroborate our assumption of crucial role of the used fragments for binding and stabilization of the target. Nonetheless, the most effective compounds were also not selective; stabilization range of alternative G4-forming sequences was significant (4–10 °C). Meanwhile, less stabilizing compounds N1198, N1199 and N1195 increased the melting temperature of LTR-III G4 by 4–7 °C, comparable with the best initially tested compounds. Titration experiments (Table S6) revealed that the most potent stabilizers N1193, N1196 and N1197 retained significant stabilizing ability (7–9 °C) at a LTR-III G4/ligand molar ratio of 1:20 and for N1196, N1197 a detectable increase in melting temperature was also observed at a molar ratio of 1:2. These compounds were found to be quite selective and therefore were picked for further biological studies whereas ZS260.1, the most selective hit, was used for in-depth analysis of its selectivity.

### 3.3. Mechanistic Studies

#### 3.3.1. NMR Studies of the HIV-1 LTR-III-G4 Complex with ZS260.1

To investigate the mechanism of selective ZS260.1 interaction with LTR-III G4 we performed NMR and MD studies.

The HIV-1 LTR-III G4 has unusual two-module structure (Figure 1A) and its unique G4 structural features can provide an opportunity for selective binding and stabilization by the compounds with a specific geometry of the aromatic system and the substituent arrangement. Here, we studied the interaction of the most promising LTR-III G4-selective compound ZS260.1 in comparison with its non-stabilizing structural congener N979 (the structure of N979 can be found on Figure S4-1) using NMR.

**Figure 4:**
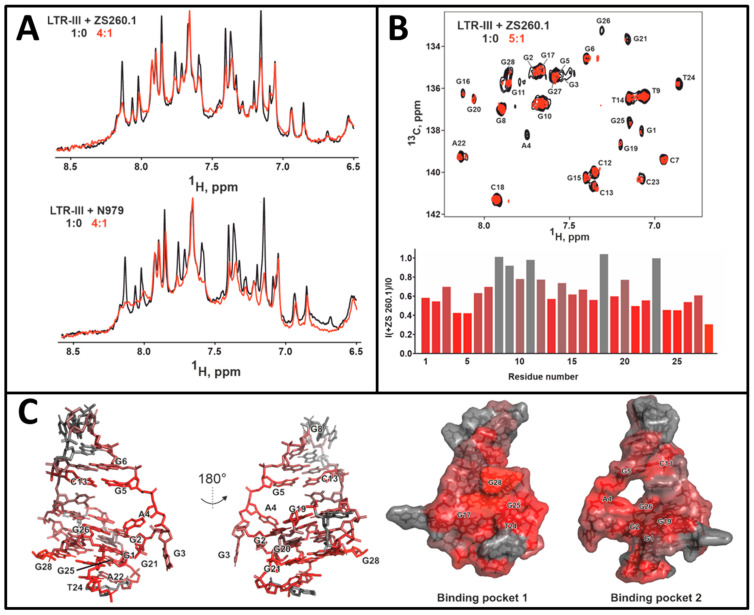
The results of NMR titration studies. (**A**) Changes in 1D ^1^H spectra (region, corresponding to pyrimidine H6 and purine H8 protons) of 50 μM LTR upon titration with ligands ZS260.1 and N979. (**B**) Overlay of ^1^H-^13^C HSQC spectra of 0.5 mM LTR before (black) and after (red) addition of ZS260.1 (LTR-III/ZS260.1 = 5:1) at 25 °C (**top**). The assignment of H6/C6 (T, C) and H8/C8 (A, G) cross-peaks is shown. Diagram corresponds to the relative decrease in intensities upon the addition of ZS260.1 (**bottom**). (**C**) Nucleotides of the LTR-III G4 structure (PDB: 6H1K), most sensitive to the addition of ZS260.1 (**left**) and two possible binding pockets (**right**). The structure is colored according to attenuation of H6/C6 and H8/C8 cross-peaks in ^1^H-^13^C HSQC spectrum (panel **B**, **bottom**).

Titration of LTR-III by ZS260.1 and N979 resulted in a drop of intensity of some LTR-III signals in HN (13.0–10.5 ppm) and H6/H8 (8.5–6.5 ppm) regions of the ^1^H NMR spectrum (Figure 4A). The observed selective signal attenuation indicates that both ligands interact with the LTR-III molecule. The ligand binding processes are probably in intermediate to fast regime on the NMR timescale. Therefore, the resonances of residues that are close to the ligand binding site (s) become broadened and attenuated (intermediate exchange regime). At the same time the nucleotides that are far from the ligand binding site (s) demonstrated only weak changes in chemical shifts and was not broadened (see Figure S8-1, fast exchange regime). Note that the formation of complex with ligand does not necessary imply stabilization of the G4 structure.

We assessed the nucleotides that most likely take part in the binding of ZS260.1 by measuring attenuation of H6/C6 and H8/C8 cross-peaks in ^1^H-^13^C HSQC spectrum (Figure 4B). There are three clusters of residues most sensitive to ZS260.1 addition: on the bottom part of LTR-III G-quadruplex (G21, T24, G25, G28), on the top part of G-quadruplex (G1, G2, G19, G26), and in the loop region in the cavity between two modules (A4, G5, C13) (Figure 4C). The position of these nucleotides implies that there may be two ligand binding pockets on the surface of LTR-III: formed by G17, T24, G25, G28, and thus stabilizing 3′-terminus of LTR-III G4 (binding pocket 1); and formed by G1, G2, A4, G5, C13, G19, G26 in the cavity between the two modules stabilizing 5′-terminus of LTR-III G4 (binding pocket 2, Figure 4C).

To determine affinity of the compound ZS260.1 to LTR-III G4 we analyzed changes in H8 chemical shift of G8 residue in 1D ^1^H NMR spectra. This nucleotide is several residues apart from the second binding pocket and therefore demonstrates fast (on the NMR scale) chemical exchange. The titration data were successfully approximated by simple chemical reaction A + B = AB, that implies one to one stoichiometry of binding that is consistent with literature data (Suppl. S8) [17]. The dissociation constant for the LTR-III/ZS260.1 complex (K_d_) was on the order of 3 μM.

#### 3.3.2. MD Simulations of the LTR-III Complex with ZS260.1

**Figure 5:**
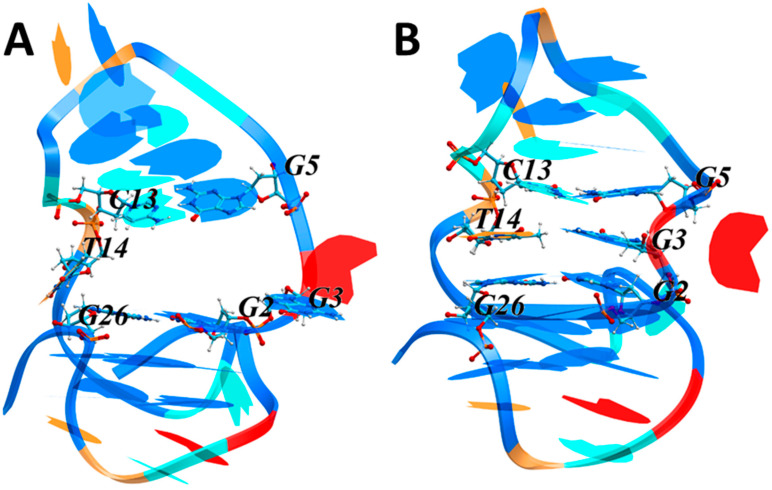
LTR-III G4 structure at the initial, 0 ns (**A**) and final, 80 ns (**B**) stages of MD simulations. The DNA shown by rendering with the nucleotides are colored as follows: G, blue; A, red; T, light brown; C, cyan. Cavity-forming nucleotides are in ball-and-stick representation. Atoms are represented by the following colors: carbon, ice blue; oxygen, red; nitrogen, blue; and hydrogen, white.

NMR spectroscopy did not allow establishing the structure of the complex, but only indicated the most probable sites of interaction. Therefore, to clarify the interaction mechanism and estimate the energies of possible LTR-III/ZS260.1 complexes we studied the target-ligand interaction in silico. To obtain the initial model of the LTR-III G4-duplex hybrid, we used the reported structure [PDB 6H1K] resolved by NMR spectroscopy [12]. Analysis of the NMR data revealed two possible states of the LTR-III hybrid: with or without cavity between the hairpin and the G4 modules. We questioned which of the two states is the most likely. Therefore, prior to investigating the hybrid-ligand interactions, we simulated molecular dynamics (MD) of the LTR-III hybrid structure. The conformation with the maximum volume of the cavity was used as the starting point. Figure 5 shows the initial and final conformations.

To illustrate the changes in the cavity geometry during the MD simulation, we used the following parameters characterizing the arrangement of nucleotides that form the boundary of the cavity: center of mass (COM) distances between the nucleobases and the angle between the normals to the planes in which the nucleobases are located. Time plots of the COM distances and the angles between the normals (Φ) are shown in Figure S9-1. The T14 residue, initially exposed to the external environment, began to enter the cavity after ~0.06 ns, and its entrance halved the cavity volume. Then, G3 began to enter the cavity after ~2.19 ns, embedding between/stacking with G2 and G5 and locating in the same plane as T14. As a result, the cavity got closed almost at the beginning of the MS simulation. Changes in various contributions to LTR-III free energy upon the closure of the cavity are shown in Figure S9-2.

The van der Waals contribution significantly increased upon the closure of the cavity as a result of stacking interactions of T14 and G3 with the adjacent nucleobases (Figure S9-2C). At the same time, the decrease in surface accessible to the solvent led to an increase in the hydrophobic contribution to the free energy (Figure S9-2E). Notably, the LTR-III hairpin is a diagonal loop, which makes the contact between T14 and G3 particularly advantageous. We concluded that the cavity formation was a possible but unlikely event.

**Figure 6:**
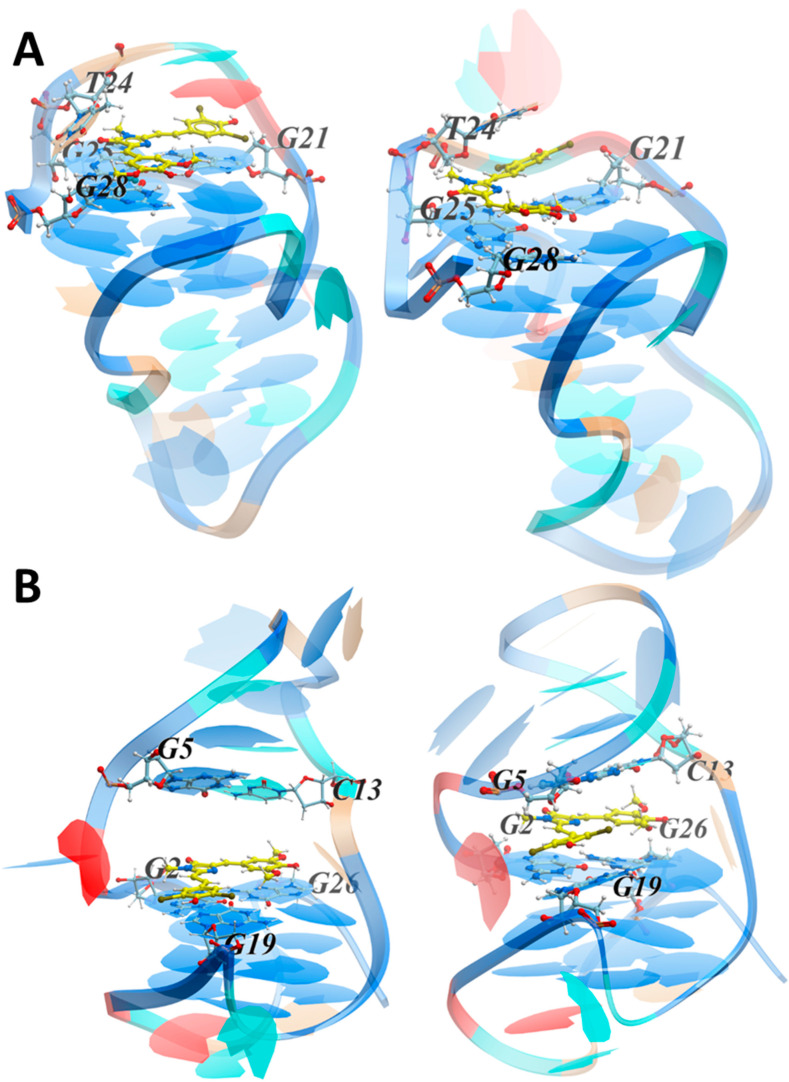
The conformations of the complexes with ZS260.1 on the outer G tetrad (**A**, complex 1) and within the cavity (**B**, complex 2) of the LTR-III structure at the initial, 0 ns (**left**) and final, 80 ns (**right**) stages of MD simulations. The DNA shown by rendering with the nucleotides are colored as follows: G, blue; A, red; T, light brown; C, cyan. Cavity-forming nucleotides and ligand are in ball-and-stick representation. Atoms are represented by the following colors: ligand carbon, yellow; DNA carbon, ice blue; oxygen, red; nitrogen, blue; and hydrogen, white.

The docking procedure was applied to search for the most probable location of ZS260.1 on the LTR-III surface. Taking into account the nonzero probability of the cavity formation, both the initial conformation with the cavity and the final one obtained at the last step of the MS simulation were used as targets. During the docking procedure, the sugar-phosphate backbone of the targets in the loops was flexible. The stability of the complexes obtained from docking was verified by MD simulations. The two most probable conformations, namely the one with ZS260.1 located on the outer G tetrad near 3′-terminus of LTR-III G4 (Figure 6A, complex 1) and the other with ZS260.1 within the cavity near 5′-terminus (Figure 6B and Figure S10, complex 2) at the initial (left) and final (right) stages of MD simulations are shown in Figure 6. Both structures of the LTR-III/ZS260.1 complexes obtained from docking were stable throughout the simulation. An analysis of the contributions to the interaction energy of the complexes are shown in Figure S11-1.

In complex 2 (the ligand is located in the cavity near 5′-terminus), the interactions were more efficient due to the van der Waals contribution resulting from the large number of nucleotides involved in stacking interactions with the ligand (Figure S11-1). The following parameters were used to analyze the ligand positioning on the LTR-III surface during the MD simulation. The distances between the COM of the involved nucleobases and the COM of the ligand aromatic rings, and the angles between the normals to the planes in which the nucleobases and the ligand aromatic rings are located. Time plots of the COM distances and angles (Φ) between the normals are shown in Figure S11-2.

For complex 1, in which the ligand was located on the outer G tetrad containing G17, G21, G25 and G28 near 3′-terminus, the dibromophenyl moiety stacked with G21, and also with A22 at the initial stage of the MD simulation. The interaction with A22 turned out to be unstable, and the stacking interaction with A22 was replaced by that with T24. The imidazolone ring was located above G25 and the dimethoxyphenyl moiety stacked with G28. These results are in good agreement with the NMR data, which revealed possible ligand interactions with G21, T24, G25 and G28 (binding pocket 1, Figure 4C). For complex 2, in which the ligand was located within the cavity, the dibromophenyl moiety stacked with G19, the imidazolone ring was located between and stacks with G2 and G5, and the dimethoxyphenyl moiety was sandwiched between and stacked with G26 and C13. These results also comply with the NMR data (binding pocket 2, Figure 4C). To conclude, if a cavity is formed between the hairpin and the G-quadruplex modules (e.g., due to thermal fluctuations), the ligand is localized in it, and the ligand-target interaction energy is maximum. However, the lifetime of the cavity is limited (the cavity-free conformation dominates), and in this case, ZS260.1 could interacts with the outer G-tetrad. Nevertheless, the ligand positioning within the LTR-III cavity (binding pocket 2) near 5′-terminus of G4 is presumably responsible for the selectivity over the other G4 targets containing outer G tetrads without additional ligand-interacting modules and for the observed thermal stabilization of LTR-III G4.

### 3.4. In Vitro Antiviral Properties

**Figure 7:**
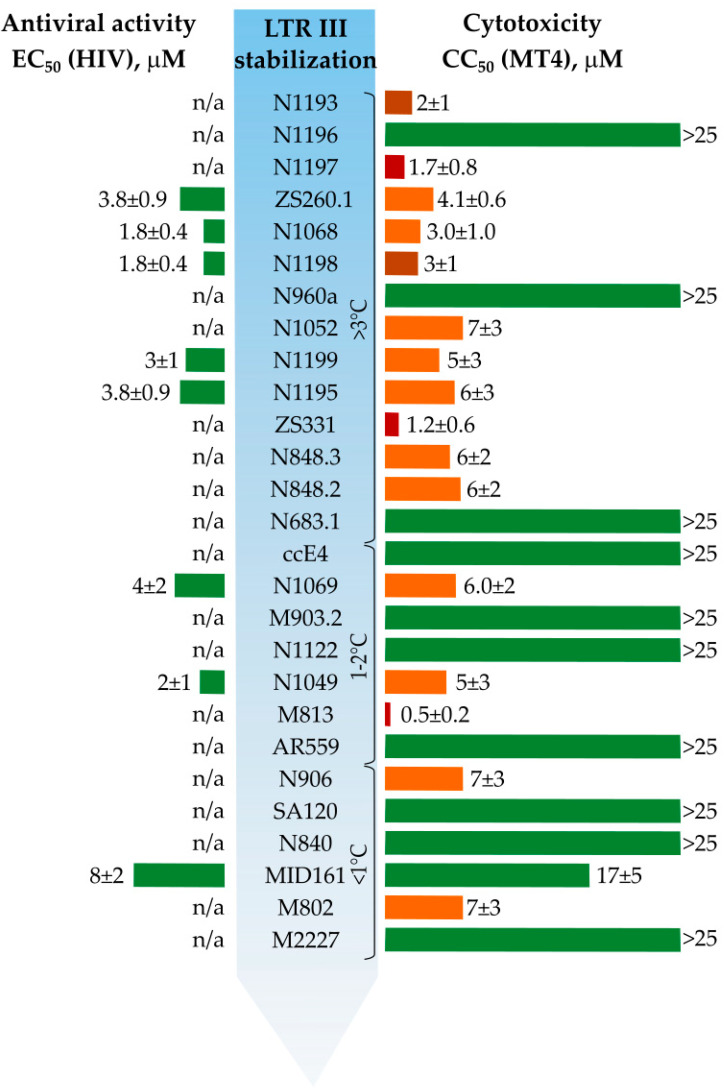
Thermal stabilization, cytotoxicity, and antiviral activity of the tested compounds in MT4 cells against HIV-1 (st. NL4-3). n/a—not active in non-toxic concentrations.

In vitro antiviral properties for the selected compounds were assessed in comparison with cytotoxicity (Table S12, Figure 7). Most of the compounds were toxic for MT4 cells (cytotoxic concentration (CC_50_) < 10 μM). None of the 10 non-toxic compounds (CC_50_ > 25 μM) inhibited HIV reproduction in MT4 cells. LTR-stabilizing compounds ZS260.1, N1068 and N1198 showed inhibitory activity with EC_50_ in micromolar range, although accompanied by significant cytotoxicity. The cytotoxicity mainly increased with LTR stabilization properties and correlated with inhibitory activity indicating that the compounds might have analogous targets in viral and human genomes, taking into account the presumable existence of quadruplex–duplex hybrids in the human genome [41]. The use of a limited G4 target panel seemed reasonable for rough selectivity evaluation in our screening assays. However, as evident from the inhibitory assays, more detailed selectivity analyses will be needed in future rounds of optimization. In addition, side effects of the selected compounds could also arise from the binding to intracellular proteins [42].

To summarize, comparison of LTR-III G4 stabilization data and antiviral properties in some cases reveal no strong correlation. However, the absence of detectable antiviral activity for some of the potent LTR-III G4 stabilizers could be the result of their high cytotoxic activity (low CC_50_ values, e.g., N1197).

**Figure 8:**
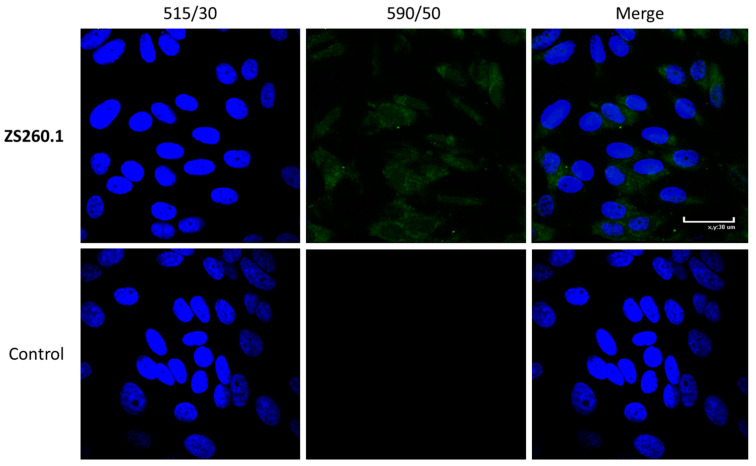
The results of confocal microscopy of the stained cells, treated with ZS260.1 or DMSO as control.

The DNA G4-mediated mechanism of action suggests the potential candidate should penetrate into the cell nucleus. Some of the previously studied GFP chromophore analogs were shown to penetrate the cell and the nucleus [43]. However, high hydrophobicity of the selected compounds may interfere with intracellular accumulation. In this regard, we tested the localization of the most promising LTR-III G4 stabilizer, ZS260.1 in the cells, using fluorescent imaging technique to visualize compound accumulation in the cells (Figure 8).

The obtained results show that the compound (visible in the green channel) is able to accumulate in the cells and can be detected both in the cytoplasm and in the nuclei of the cells (stained with Hoechst 33342).

## 4. Conclusions

GFP chromophore analogues find diverse applications for bioimaging properties. However, their potential as drug leads is rather under-investigated. In this work we screened a large in-house library of synthetic GFP chromophore analogues as ligands for promising target – HIV-1 LTR-III sequence. The screening revealed a number of potent stabilizers and allowed us to design improved structures by a fragment-based approach. Selectivity testing and in vitro antiviral activity studies revealed the most perspective lead compound—ZS260.1. The structure of the LTR-III/ZS260.1 complex was assessed using NMR studies and MD simulations, showing plausible location of the compound within the cavity between the hairpin and the G4 modules of LTR-III sequence. Thus, GFP chromophore analogues were revealed to be promising scaffolds for further antiviral drug design on the basis of G4-binding properties.

## Figures and Tables

**Table 1:** ODNs, used in this work.

Code	Sequence (5′→3′)
LTR-III	FAM-GGGAGGCGTGGCCTGGGCGGGACTGGGG-TAMRA
Telo	FAM-AGGGTTAGGGTTAGGGTTAGGG-TAMRA
dsDNA	FAM-CTATAGCGCGCTATAG-TAMRA
Pu39	FAM-AGGGGCGGGCGCGGGAGGAAGGGGGCGGGAGCGGGGCTG-TAMRA

## Data Availability

Not applicable.

## Supplementary Materials

The following are available online at https://www.mdpi.com/article/10.3390/biom11101409/s1, Table S1. Primary screening of GFP chromophore analogs and their intermediates for the stabilizing properties of FAM/TAMRA-labeled LTR-III G-quadruplex; Table S2: Comparison of stabilization of model G4s and double stranded control sequence by primary screening lead compounds; Table S3: Primary screening of newly synthesized GFP chromophore analogs for the stabilizing properties of FAM/TAMRA-labeled LTR-III G-quadruplex; Supplement S4: Structural motifs of the lead compounds in comparison with the other structurally related compounds; Table S5: Comparison of stabilization of model G4s and double stranded control sequences by newly synthesized compounds; Table S6: Titration experiments for N1193, N1196, N1197 at various DNA:ligand molar ratios; Supplement S7: Synthetic procedures for the synthesized GFP chromophore analogues; Supplement S8: NMR titration data for the LTR-III/ZS260.1 complex; Supplement S9: HIV LTR-III G4 MD simulation parameters; Supplement S10: The conformation of the LTR-III/ZS260.1 complex (additional simulation angles); Supplement S11: HIV LTR-III G4/ZS260.1 complex MD simulation parameters; Table S12: Antiviral activity of the tested compounds in MT4 cells against HIV-1 (st. NL4-3).
