# Supplementary material for: Probing GFP Chromophore Analogs as Anti-HIV Agents Targeting LTR-III G-Quadruplex
Source: Biomolecules. 2021 Sep 26;11(10):1409. doi: 10.3390/biom11101409 (PMC8533149; doi:10.3390/biom11101409)
Supplement: Supplementary file 1 [file biomolecules-11-01409-s001.zip › supplementary Biomolecules_revision2.pdf]

Supplementary Materials

Probing GFP Chromophore Analogs as anti-HIV agents targeting LTR-III G-Quadruplex

Dmitriy Y. Ryazantsev[1,†], Mikhail Yu. Myshkin[1,†], Vera A. Alferova[1,2,†], Vladimir B. Tsvetkov[3,4,†], Elena Y. Shustova[5], Polina N. Kamzeeva[1,6], Polina V. Kovalets[1], Elvira R. Zaitseva[1,6], Nadezhda S. Baleeva [1,7], Timofei S. Zatsepin [8], Zakhar O. Shenkarev[1], Mikhail S. Baranov [1,7], Liubov I. Kozlovskaya[5,9*] and Andrey V. Aralov[1,10*]

[1] Shemyakin-Ovchinnikov Institute of Bioorganic Chemistry, Russian Academy of Sciences, Moscow, 117997, Russia;
[2] Gause Institute of New Antibiotics, B. Pirogovskaya 11, Moscow, 119021, Russia
[3] World-Class Research Center "Digital biodesign and personalized healthcare", Sechenov First Moscow State Medical University, 8/2 Trubetskaya Str., Moscow, 119146, Russia
[4] Research and Clinical Center of Physical-Chemical Medicine of Federal Medical Biological Agency, Moscow, 119435, Russia
[5] Chumakov Federal Scientific Center for Research and Development of Immune-and-Biological Products of Russian Academy of Sciences, Moscow, 108819, Russia
[6] D. Mendeleev University of Chemical Technology of Russia, 9 Miusskaya Sq., Moscow, 125047, Russia
[7] Pirogov Russian National Research Medical University, Ostrovitianov 1, 117997 Moscow, Russia
[8] Center for Life Sciences, Skolkovo Institute of Science and Technology, Moscow, 121205, Russia
[9] Sechenov First Moscow State Medical University, Moscow, 119991, Russia
[10] G4_Interact, USERN, University of Pavia, Italy
* Correspondence: lubov_i_k@mail.ru (LIK), Baruh238@mail.ru (AVA)
† these authors contributed equally to this work

Contents

Table S1. Primary screening of GFP chromophore analogs and their intermediates for the stabilizing properties of FAM/TAMRA-labeled LTR-III G-quadruplex...... 3

Table S2. Comparison of stabilization of model G4s and double stranded control sequences by primary screening lead compounds. 75

Table S3. Primary screening of newly synthesized GFP chromophore analogs for the stabilizing properties of FAM/TAMRA-labeled LTR-III G-quadruplex. 76

Supplement S4. Structural motifs of the lead compounds in comparison with the other structurally related compounds. 78

Table S5. Comparison of stabilization of model G4s and double stranded control sequences by newly synthesized compounds.. 84

Table S6. Titration experiments for N1193, N1196, N1197 at various DNA:ligand molar ratios 85

Supplement S7. Synthetic procedures for the synthesized GFP chromophore analogues 86

Supplement S8. NMR titration data for the LTR-III/ZS260.1 complex.............. 124

Supplement S9. HIV LTR-III G4 MD simulation parameters 125

Supplement S10. The conformation of the LTR-III/ZS260.1 complex (additional simulation angles) 126

Supplement S11. HIV LTR-IIIG4/ZS260.1 complex MD simulation parameters 127

Table S12. Antiviral activity of the tested compounds in MT4 cells against HIV-1 (st. NL4-3). 129

Table S1. Primary screening of GFP chromophore analogs and their intermediates for the stabilizing properties of FAM/TAMRA-labeled LTR-III G-quadruplex.

No	code	Structure	LTR-III ΔT_m^a
1	AL 23	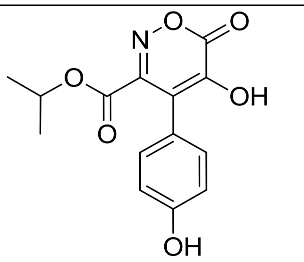	0
2	AL 25	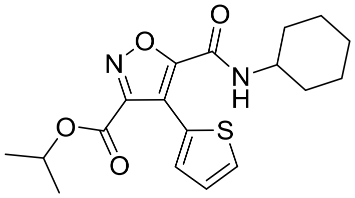	-0.1
3	AL 45	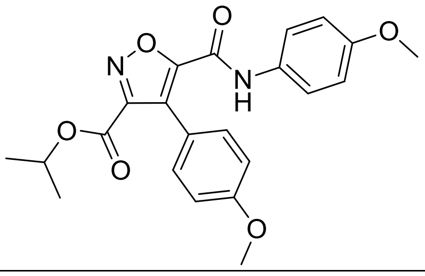	0.2
4	AL 58	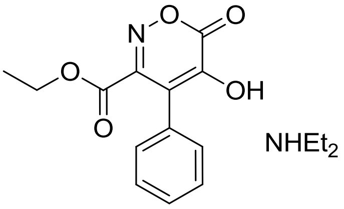	0.1
5	AI 89	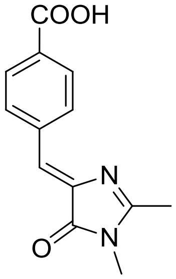	-0.5
6	AI 116	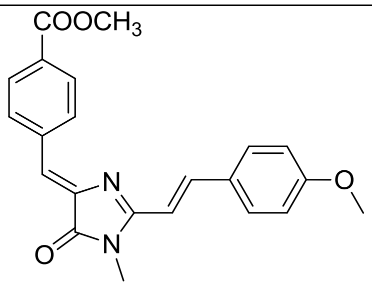	0.8

Back to Contents

7	AI 118	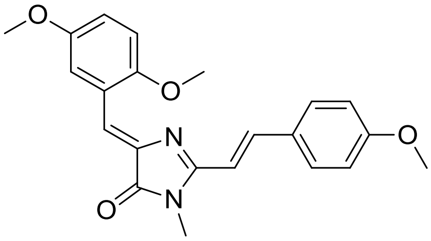	-0.1
8	AI 119	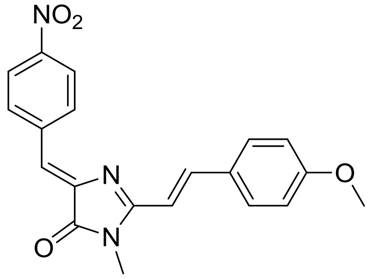	1.4
9	AL 122	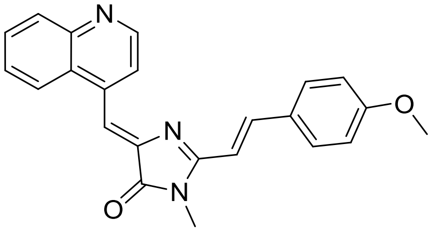	-0.6
10	AL 125	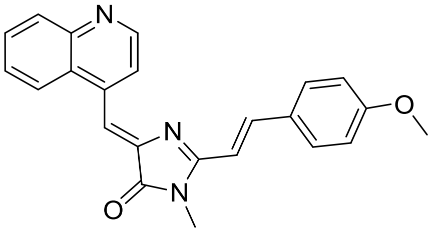	1.7
11	AI 126	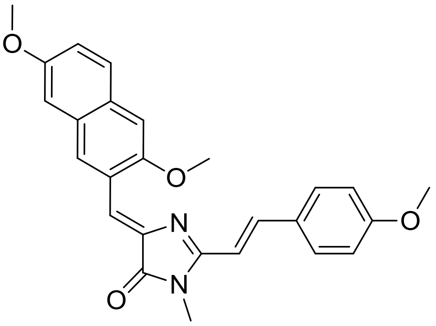	1.6
12	AL 135.2	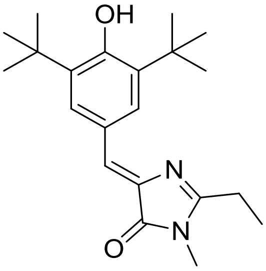	-1.3
13	AL 165	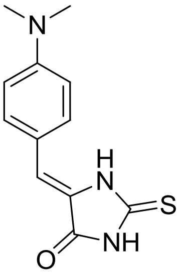	-0.2

[Back to Contents]

14	AL 166.1	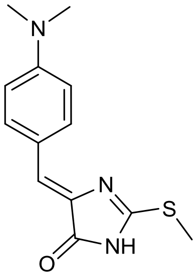	0.1
15	AL 172	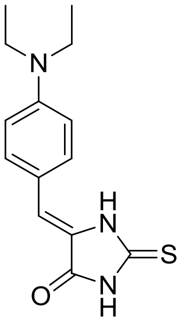	-0.2
16	Amm 75	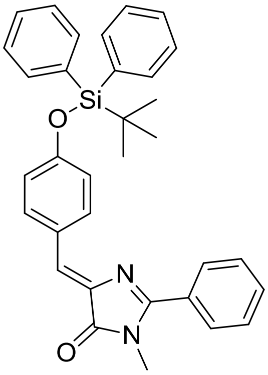	-0.3
17	Boc-AV-GFP	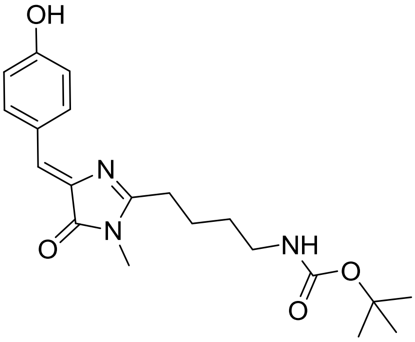	0.2
18	ccA 1	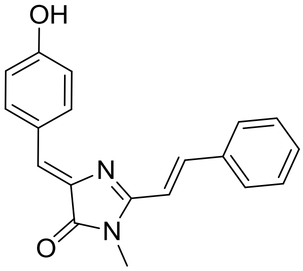	0.6
19	ccA 2	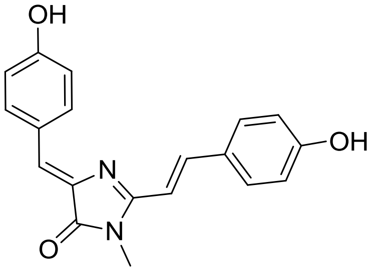	0.4

Back to Contents

20	ccA 4	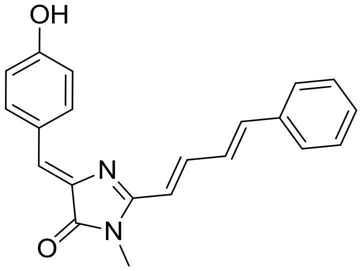	0.8
21	ccA 7	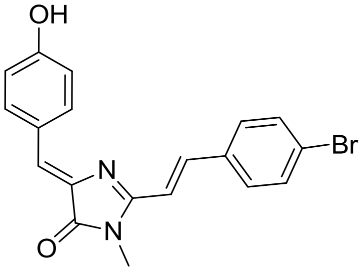	0.8
22	ccA 8	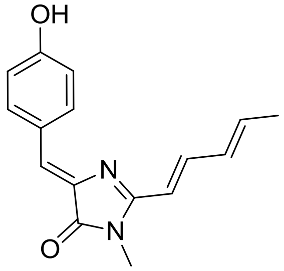	0.2
23	ccA 9	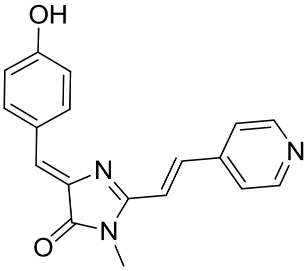	0.3
24	ccA 10	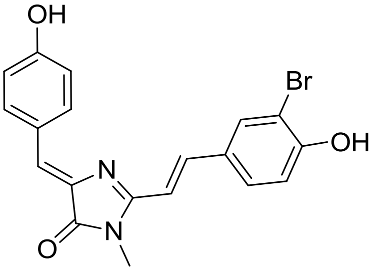	0.7
25	ccA 11	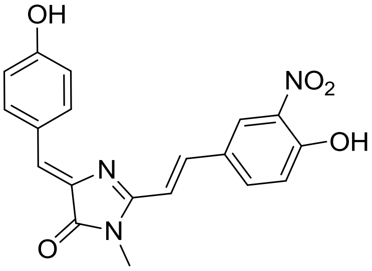	0.1
26	ccA 12	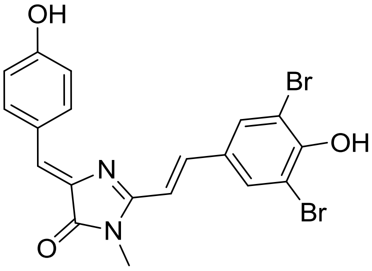	0.2

[Back to Contents](#)

27	ccA 15	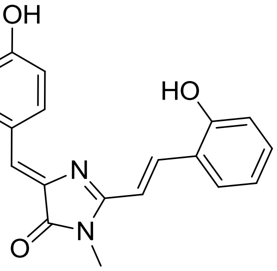	0.5
28	ccA 16	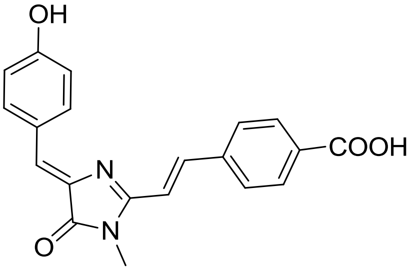	0.8
29	ccA 18	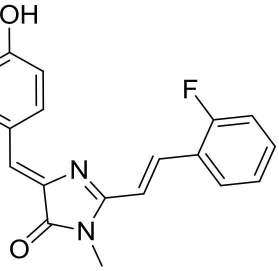	0.1
30	ccA 19	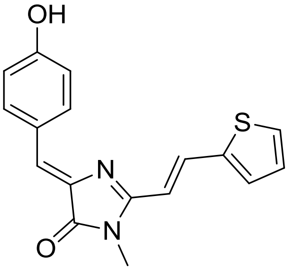	-0.1
31	ccA 20	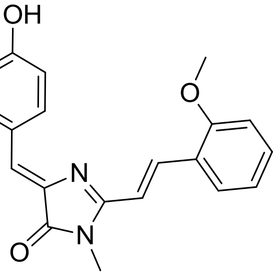	0.4
32	ccA 22	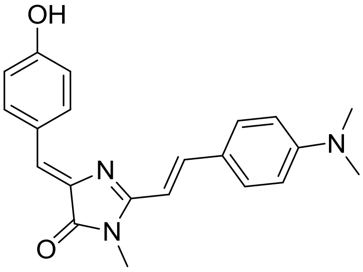	0.3
33	ccD 1	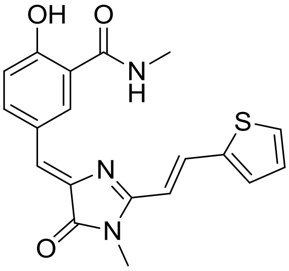	0.7

Back to Contents

34	ccD 2	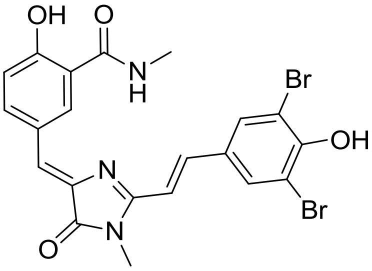	0.2
35	ccE 2	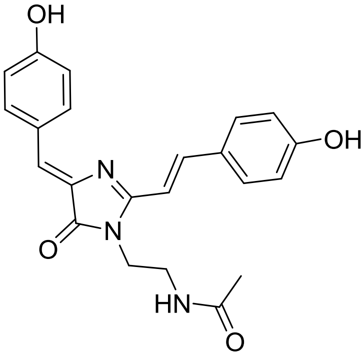	-0.1
36	ccE 4	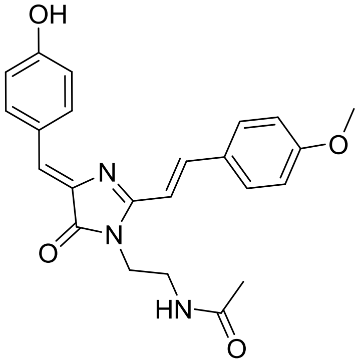	2.8
37	ccE 7	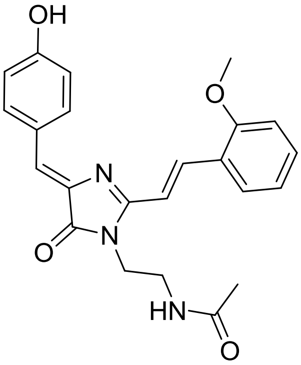	0.3
38	DOM	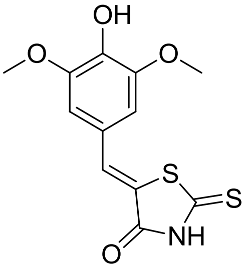	-0.6
39	GA 01	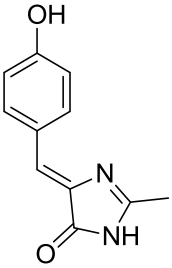	-0.4

[Back to Contents]

40	GA 02	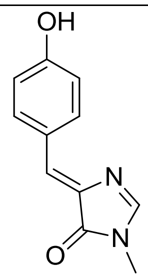	-0.4
41	GA 03	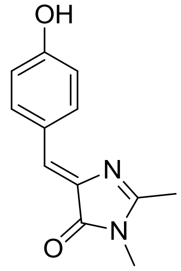	0.5
42	GA 04	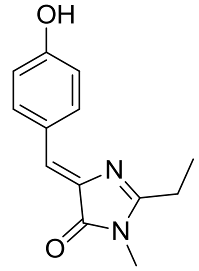	-0.1
43	GA 05	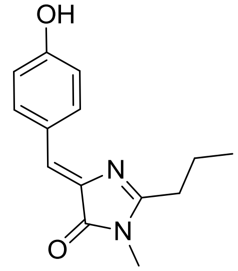	-0.5
44	GA 06	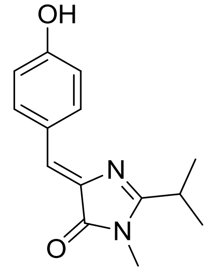	-0.7
45	GA 12	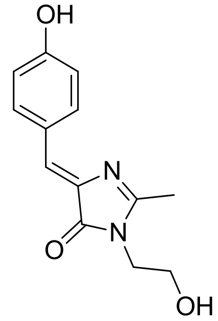	0.3

Back to Contents

46	GA 14	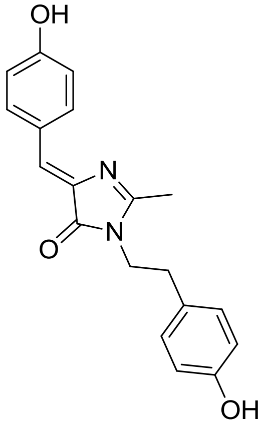	0.1
47	GA 15	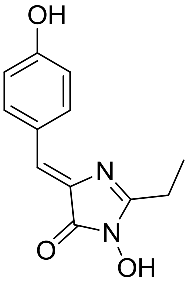	-0.1
48	GA 16	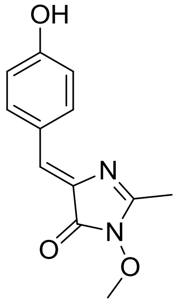	0.3
49	GA 18	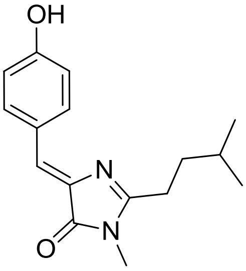	-0.1
50	GC 01	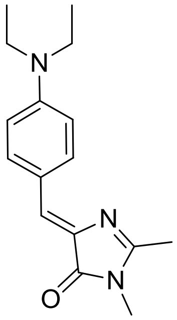	0.4

Back to Contents

51	GC 03	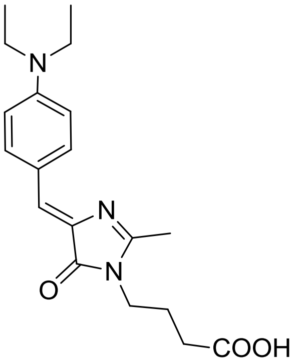	0.4
52	GC 04	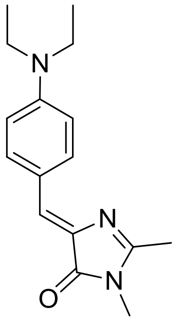	0
53	GC 07	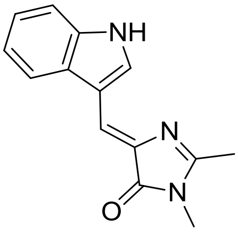	-0.4
54	GFP-2-Bn	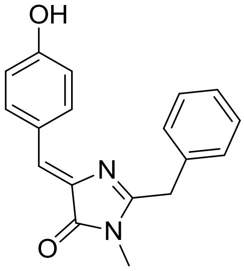	-1.5
55	GR 161	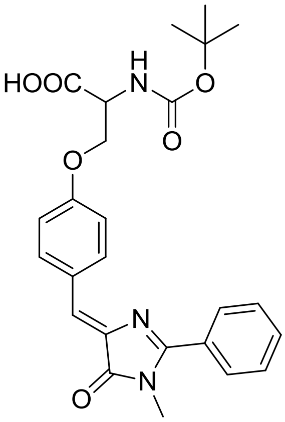	-0.2
56	GR 83	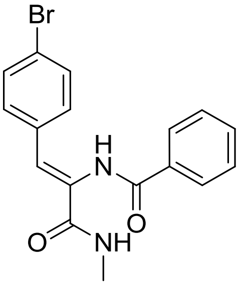	-0.8

Back to Contents

57	HBR	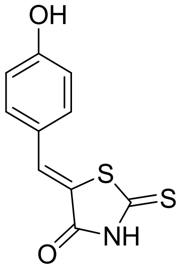	-0.2
58	HMBR	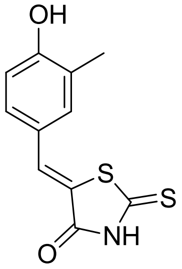	-0.6
59	JL 06.1	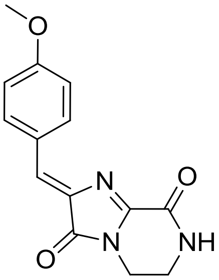	0.1
60	JL 06.2	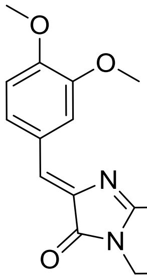	0.4
61	JL 19	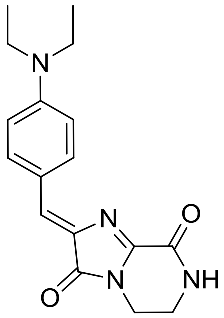	0.1
62	M 165	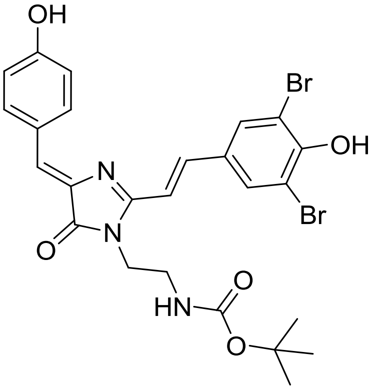	-0.5

[Back to Contents]

63	M 202	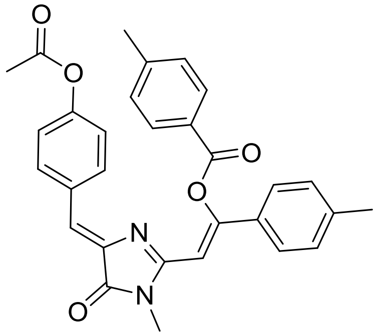	-0.6
64	M 228	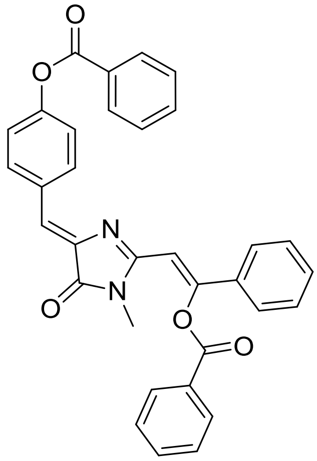	-0.8
65	M 292	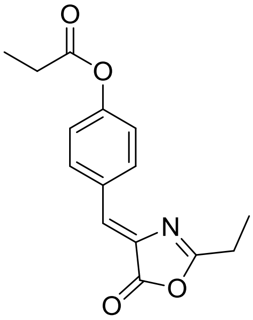	-0.6
66	M 295	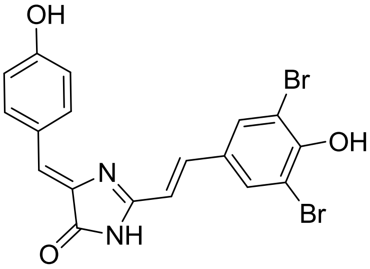	-0.5
67	M 312	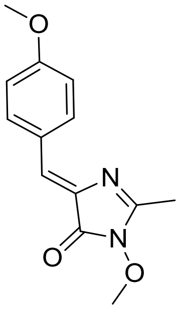	-0.2

Back to Contents

68	M 316	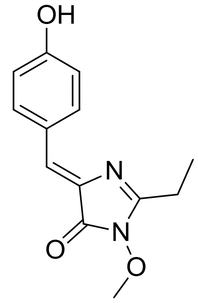	-0.3
69	M 440	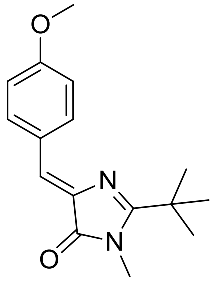	-0.1
70	M 497a2	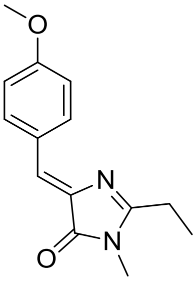	-0.4
71	M 552	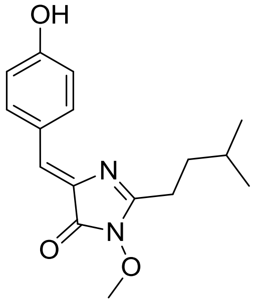	0
72	M 556	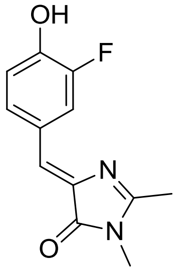	-0.2
73	M 577	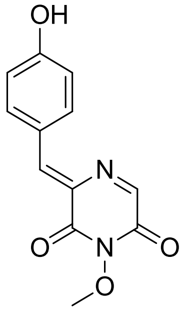	0.1

Back to Contents

74	M 653	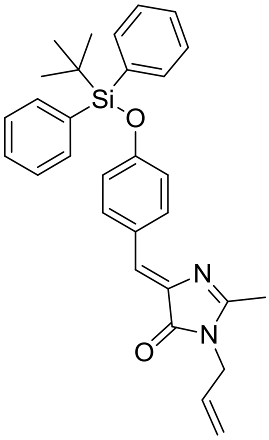	-0.2
75	M 678	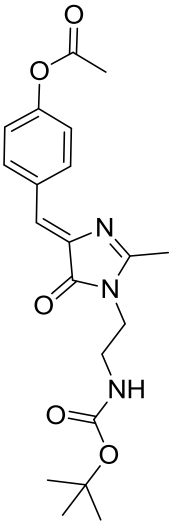	-0.4
76	M 739	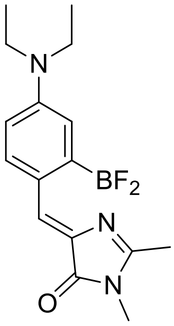	-0.4
77	M 747	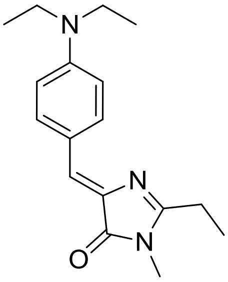	0.6
78	M 750	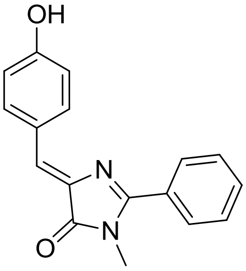	-0.2

[Back to Contents]

79	M 800	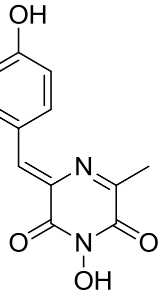	-0.2
80	M 802	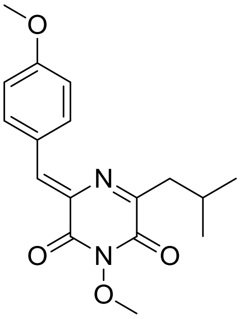	-2.3
81	M 803	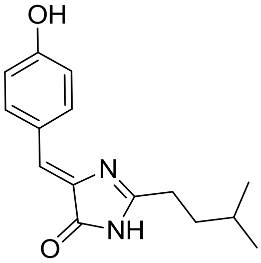	-0.1
82	M 813	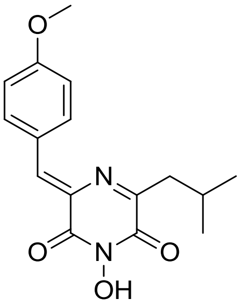	2.9
83	M 820	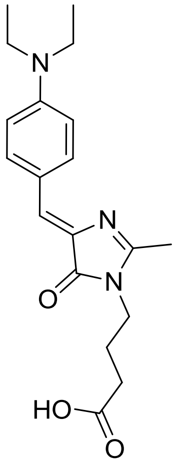	0.1

Back to Contents

84	M 894	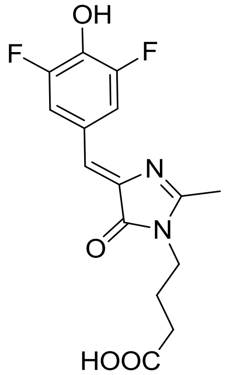	-0.3
85	M 895	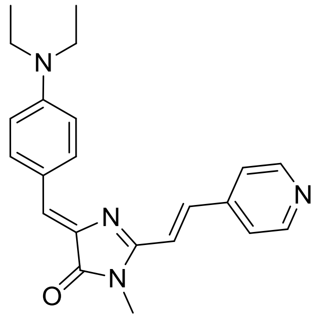	0.8
86	M 903.2	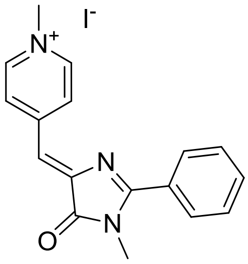	2.8
87	M 920	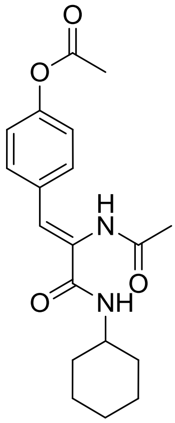	-0.4
88	M 939	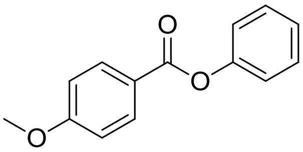	-1.1
89	M 958A	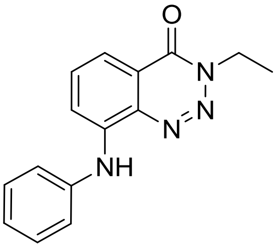	-0.7

Back to Contents

90	M 995a	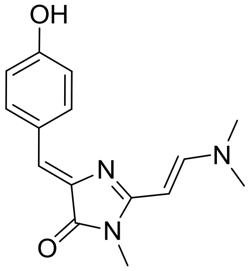	0.1
91	M 996a	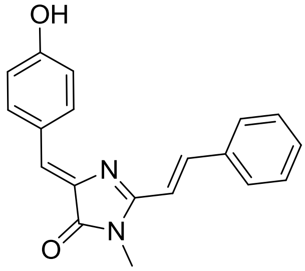	0.1
92	M 1009A	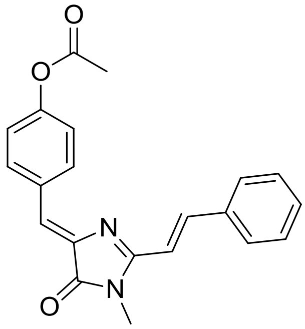	0.5
93	M 1009B	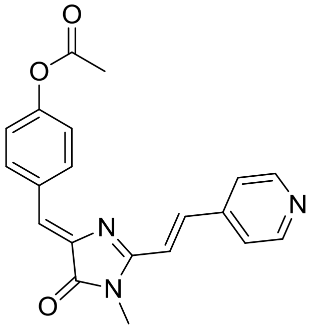	0.5
94	M 1045	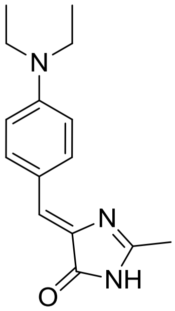	0.1
95	M 1122	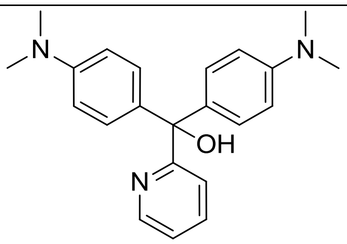	-0.6

Back to Contents

96	M 1136	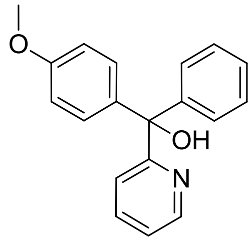	-0.7
97	M 1152	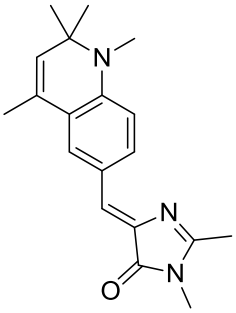	0.6
98	M 1157	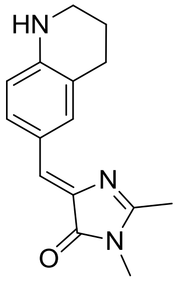	0
99	M 1165	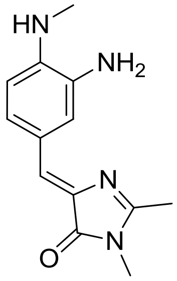	0.9
100	M 1255	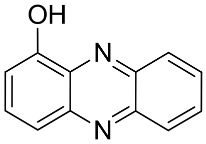	-0.6
101	M 1257	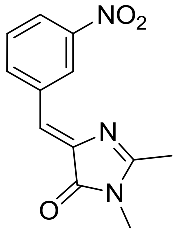	-0.9
102	M 1300	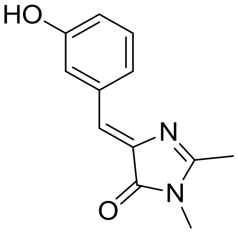	0.2

Back to Contents

103	M 1343	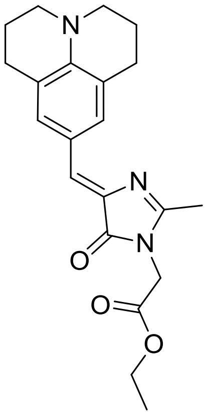	0
104	M 1352	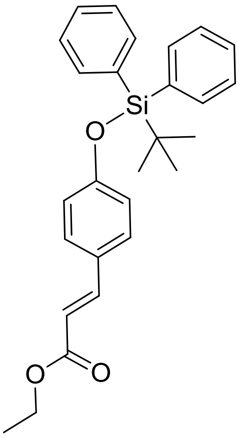	-0.5
105	M 1359	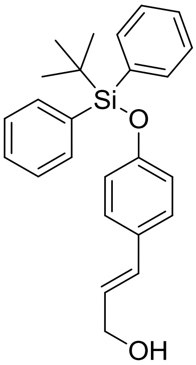	0.1
106	M 1362.2	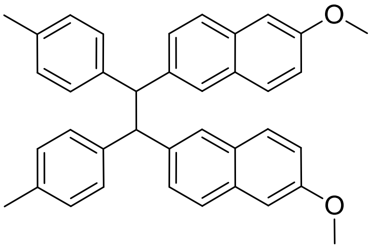	-0.6
107	M 1373.2	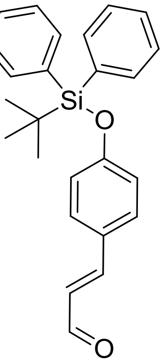	-0.5

Back to Contents

108	M 1378	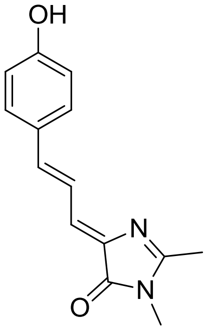	-0.7
109	M 1379.2	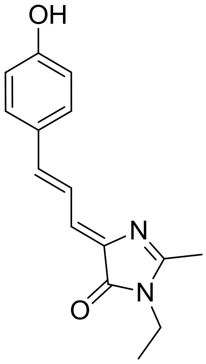	-0.2
110	M 1505	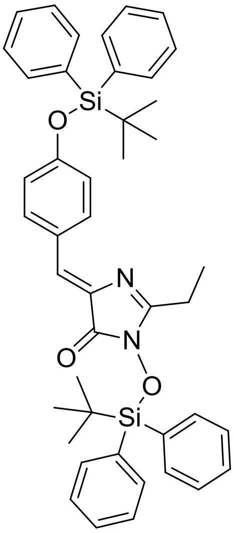	-0.3
111	M 1550	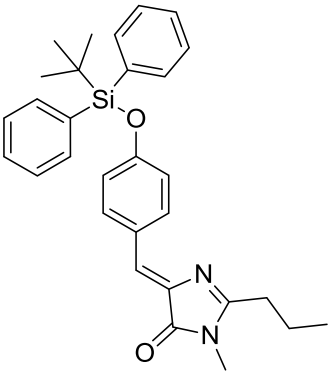	-0.1

[Back to Contents]

112	M 1557	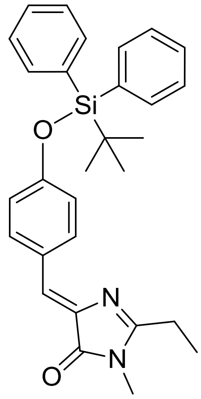	-0.1
113	M 1583.2	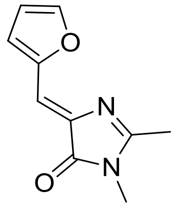	-0.5
114	M 1583.3	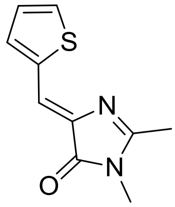	0.1
115	M 1583.4	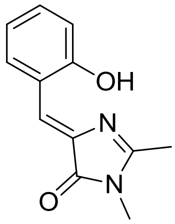	0.2
116	M 1583.5	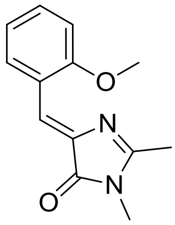	0.1
117	M 1583.6	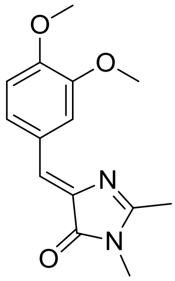	0
118	M 1583.7	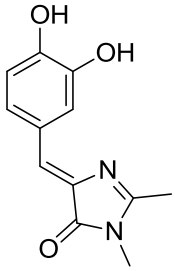	-1.4

[Back to Contents]

119	M 1586	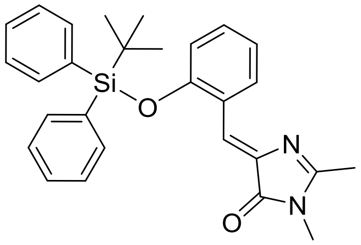	-0.3
120	M 1600	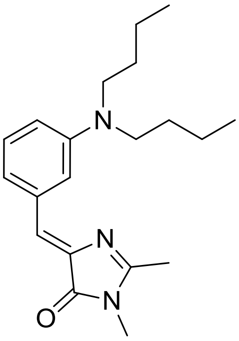	-0.4
121	M 1620	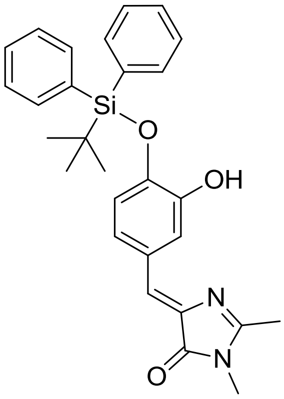	0.4
122	M 1661	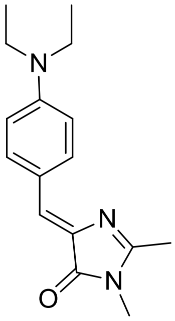	0.4
123	M 1668	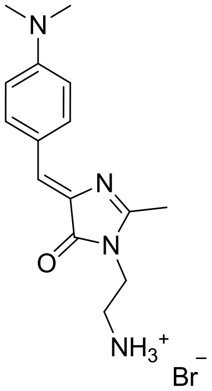	0.2

Back to Contents

124	M 1706	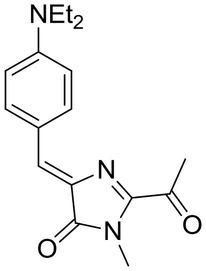	-0.4
125	M 1814	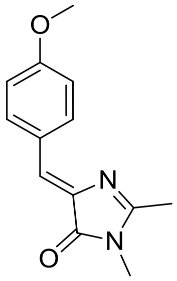	-0.3
126	M 1831.1	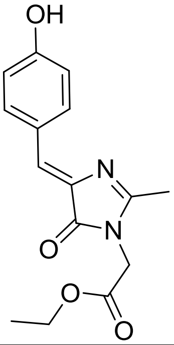	-0.2
127	M 1862	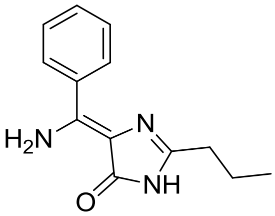	-1.1
128	M 1878.1	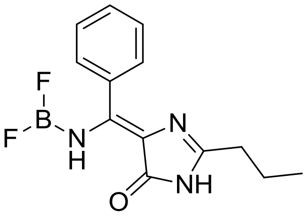	-1.5
129	M 1933	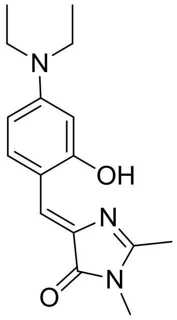	-0.3

Back to Contents

130	M 1944b	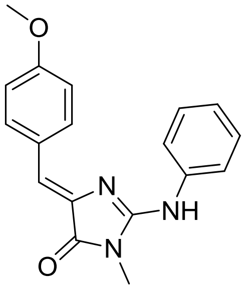	-0.3
131	M 1945.1	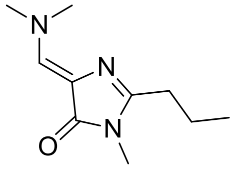	-0.6
132	M 1971a	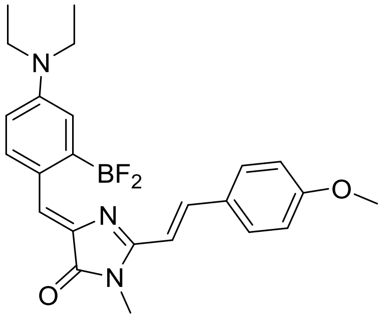	-0.3
133	M 1971c	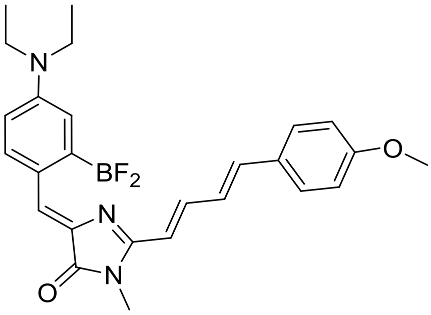	0.6
134	M 1974	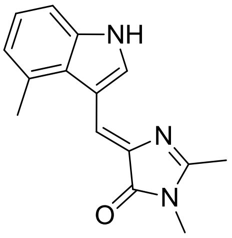	0
135	M 1975	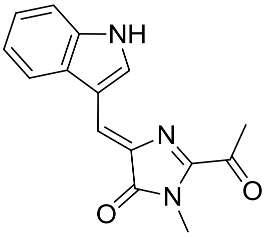	-0.6
136	M 1977	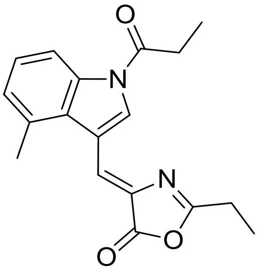	0

Back to Contents

137	M 1978	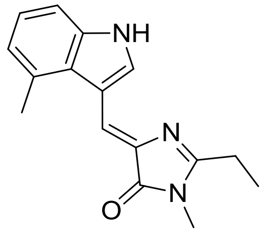	-0.1
138	M 1979	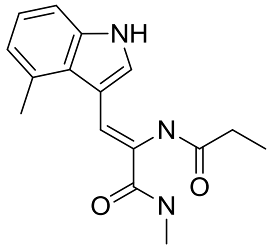	-0.1
139	M 1981	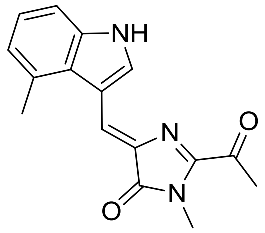	1.7
140	M 1998	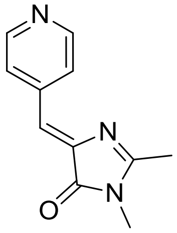	-0.4
141	M 2022a	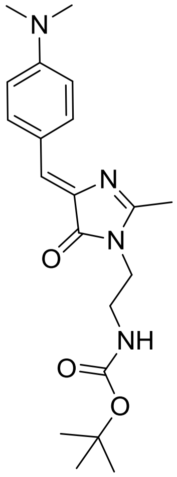	-0.2
142	M 2022b	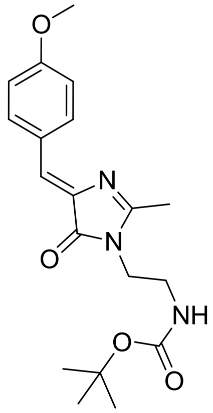	0

[Back to Contents](#)

143	M 2022d	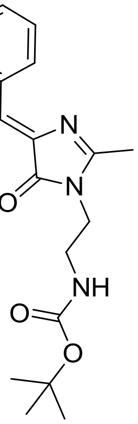	-0.3
144	M 2022e	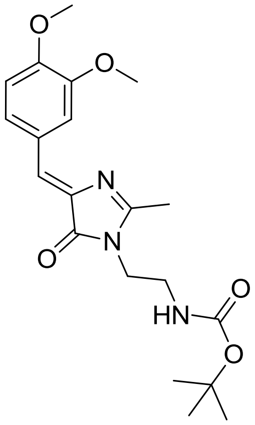	0
145	M 2022f	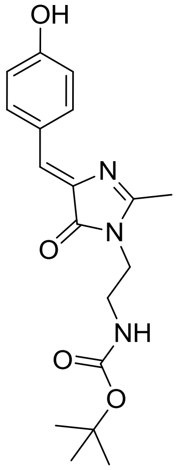	-0.5
146	M 2026c	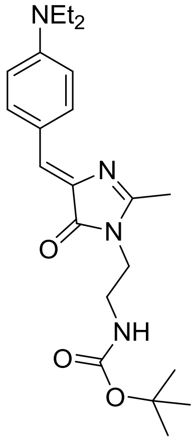	0.2

Back to Contents

147	M 2026f	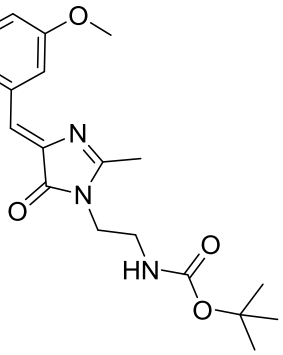	-0.4
148	M 2059	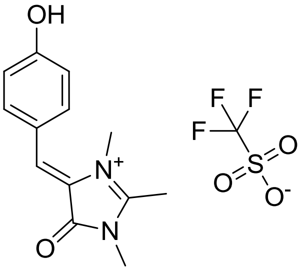	-0.7
149	M 2060	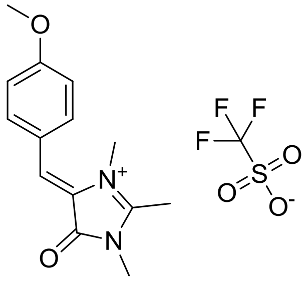	-0.2
150	M 2069	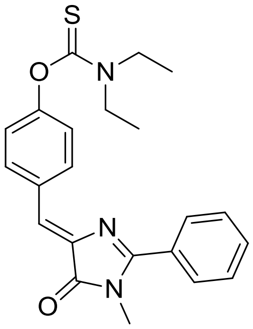	-0.4
151	M 2075	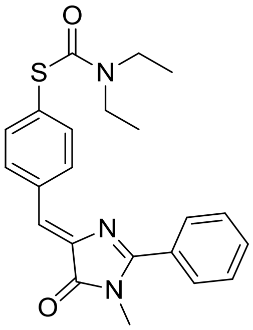	0.3
152	M 2081	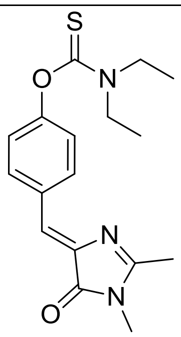	0.1

Back to Contents

153	M 906	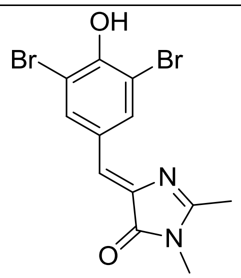	-2.7
154	M 2237	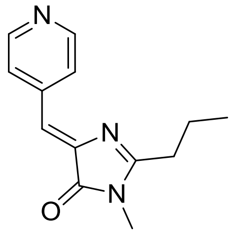	-0.3
155	M 2250.2	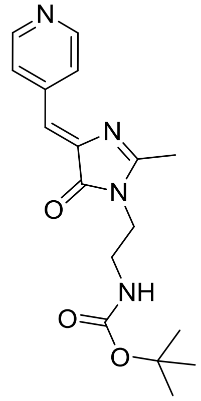	-0.1
156	M 2273.1	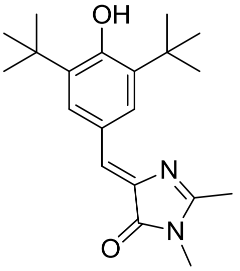	0.2
157	M 2286.1	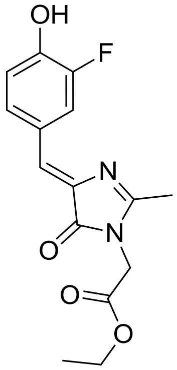	-0.3
158	M 2296	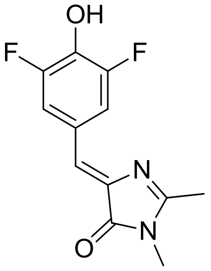	-0.4

Back to Contents

159	M 2301	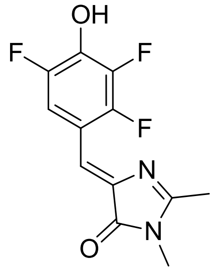	0.2
160	M 2360	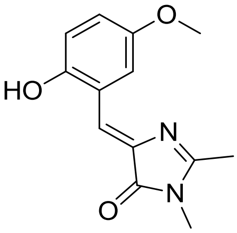	-0.3
161	M 2368b2	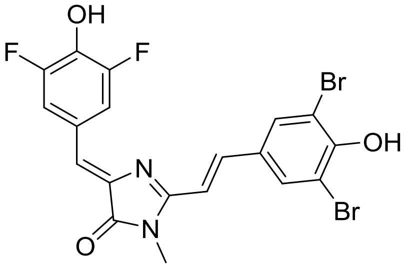	-0.9
162	M 2368c	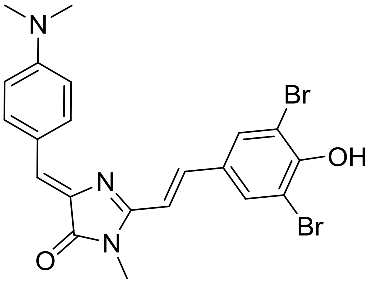	0
163	M 2368d	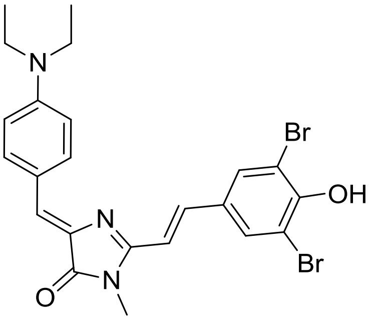	0.1
164	M 2370	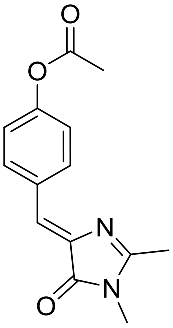	0

Back to Contents

165	M 2371c	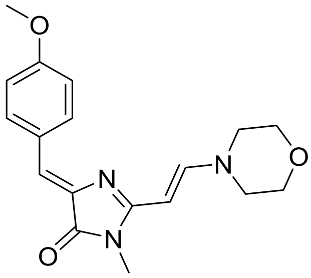	-0.1
166	M 2372a	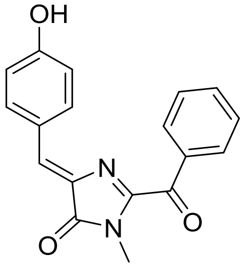	0.1
167	M 2372в	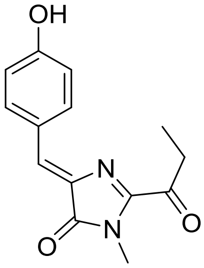	-0.2
168	M 2375	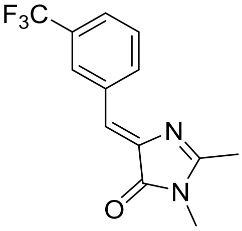	-0.2
169	M 2379	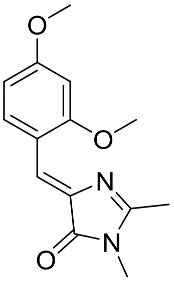	0.1
170	M 2385a	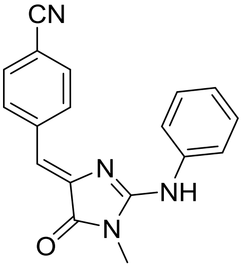	-0.1
171	M 2385b1	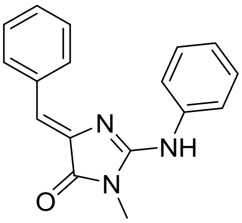	-0.1

Back to Contents

172	M 2482	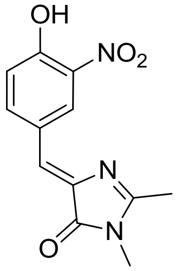	-1.2
173	M 2486	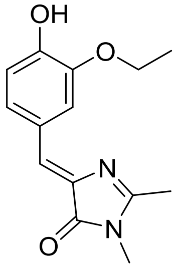	0
174	M 2489.2	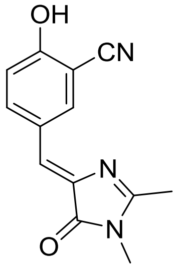	-0.5
175	M 2491	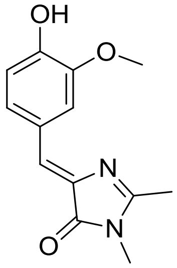	-0.2
176	M 2497	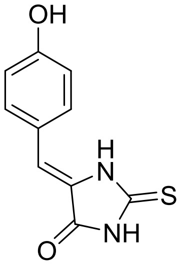	0
177	M 2499a	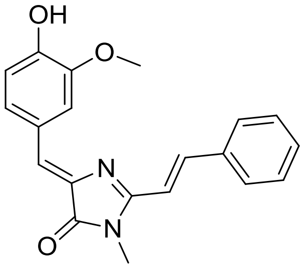	0.1
178	M 2499b	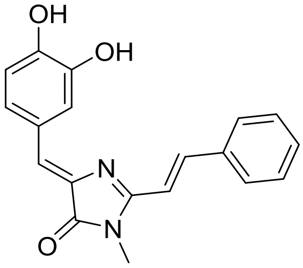	0.3

Back to Contents

179	M 2738c	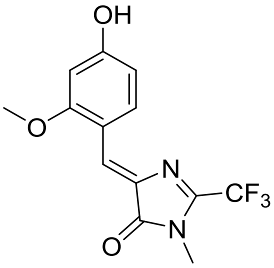	0.4
180	M 2738d	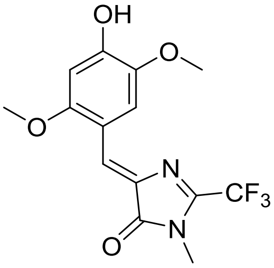	1.4
181	M 2761a	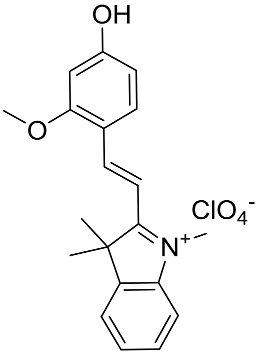	0.5
182	M 2761b	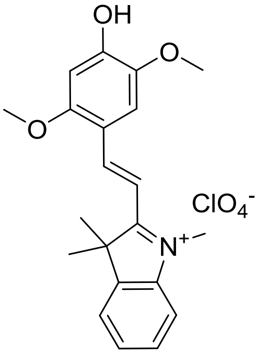	0.8
183	M 2766	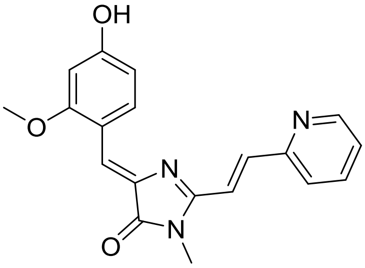	1.1
184	M 2767	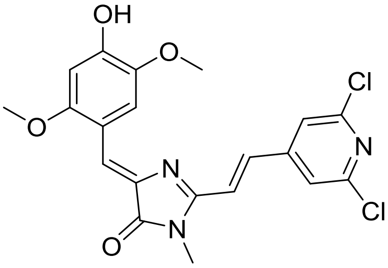	1.4

[Back to Contents](#)

185	MID 145	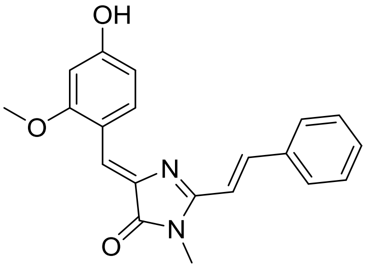	0.9
186	MID 147	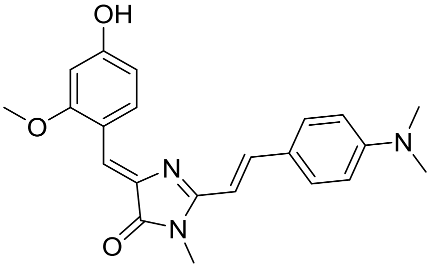	0.4
187	MID 151	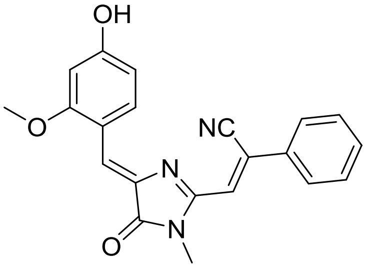	0.2
188	MID 153	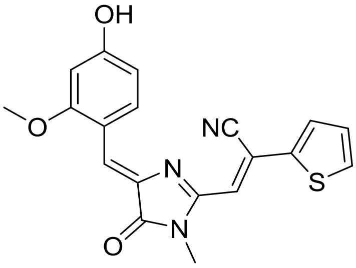	2
189	MID 161	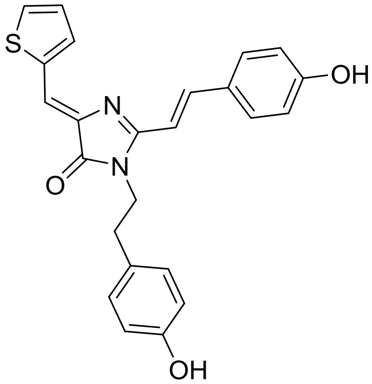	-2.6
190	MID 213	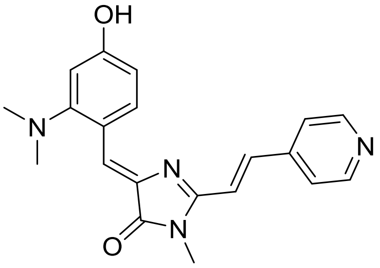	0.9
191	N 49	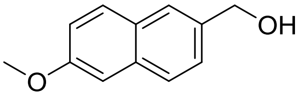	-0.1

[Back to Contents]

192	N 62	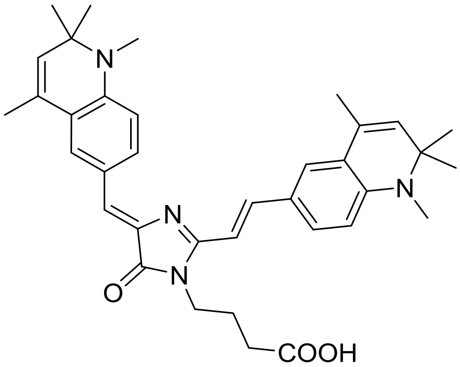	0.6
193	N 141	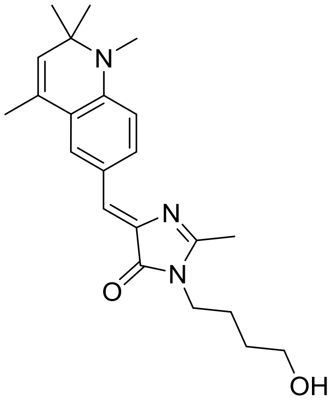	0.2
194	N 152	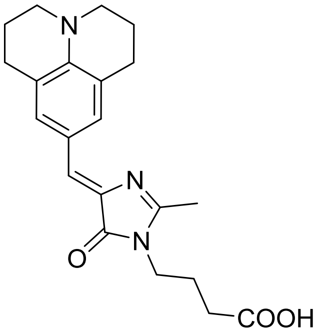	0.3
195	N 154	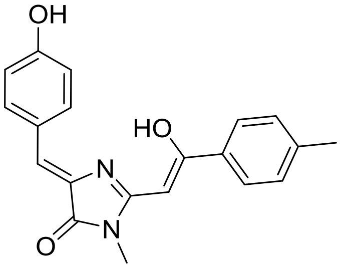	-0.1
196	N 162	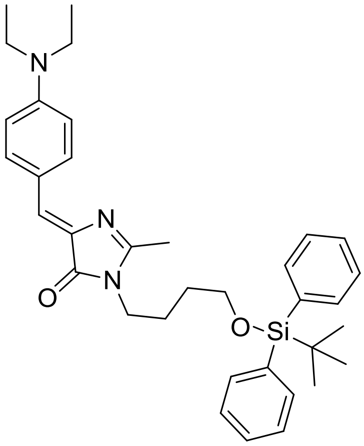	0.2

[Back to Contents](#)

197	N 182	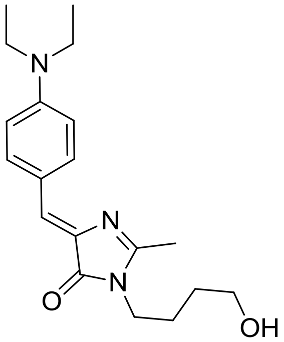	-0.1
198	N 189	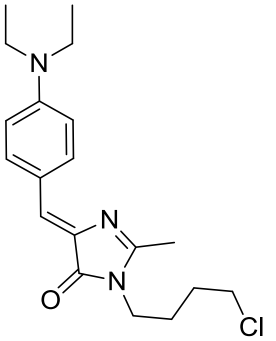	0.2
199	N 357	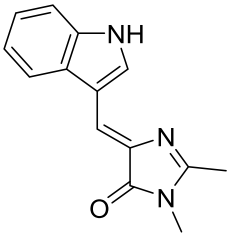	0.1
200	N 367	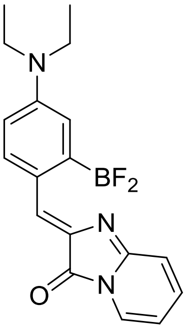	0.3
201	N 537	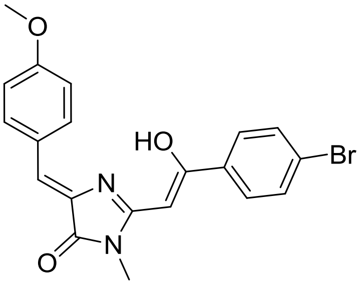	-0.3
202	N 539.1	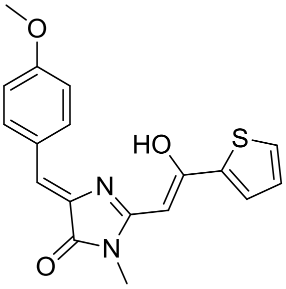	0

Back to Contents

203	N 555.3	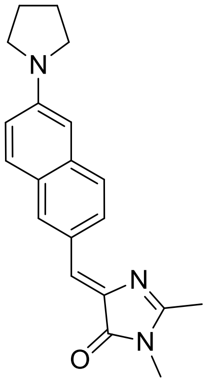	0.7
204	N 576.2	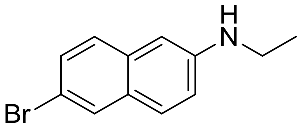	0
205	N 576.3	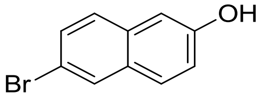	0
206	N 582.1	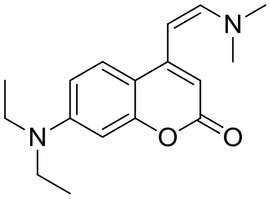	0
207	N 599.2	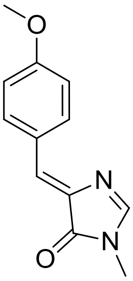	-0.7
208	N 601.23	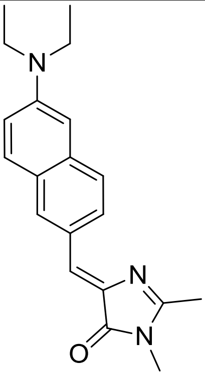	-0.7
209	N 624	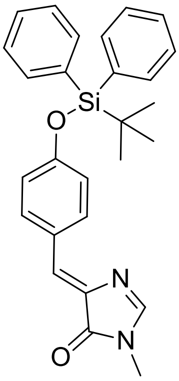	-0.5

Back to Contents

210	N 641	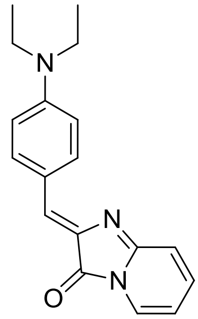	-0.5
211	N 652.3	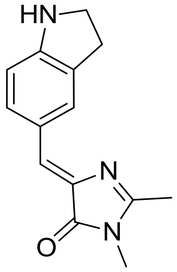	0.3
212	N 665	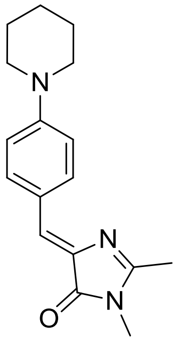	-0.3
213	N 683.1	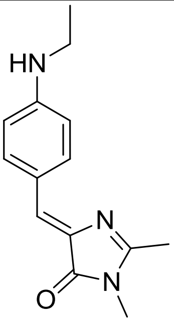	5
214	N 702.1	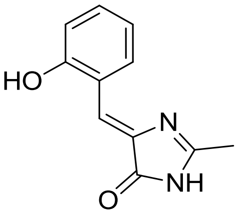	0
215	N 704	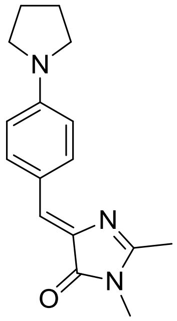	-0.2

Back to Contents

216	N 708.2	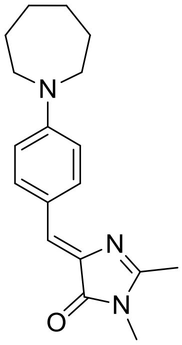	0.2
217	N 710.12	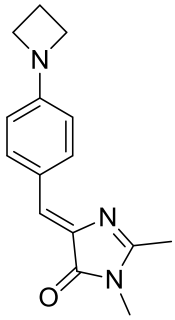	0.1
218	N 768	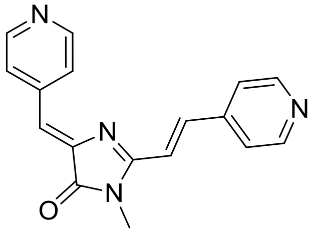	0.9
219	N 781	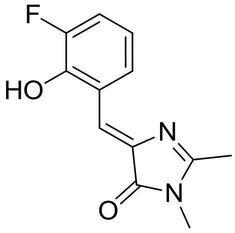	0
220	N 792	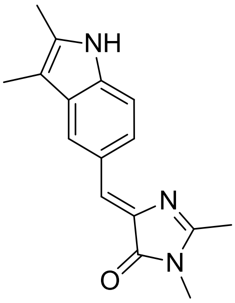	0.2
221	N 813	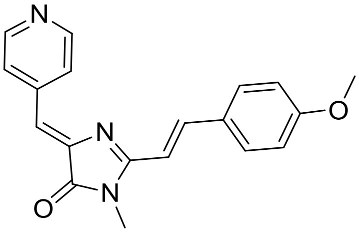	0.6
222	N 815	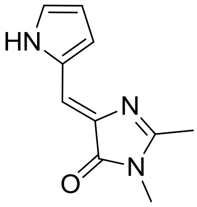	-0.1

Back to Contents

223	N 817	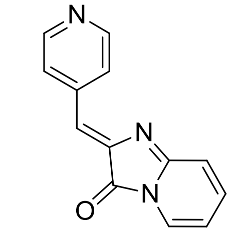	0
224	N 817.2	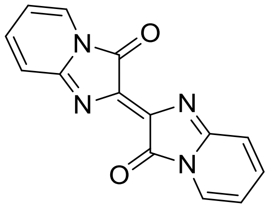	-0.1
225	N 830A	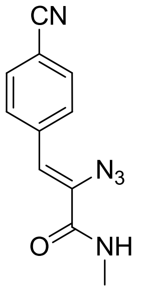	-0.1
226	N 830b	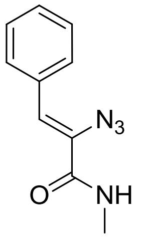	-0.2
227	N 831	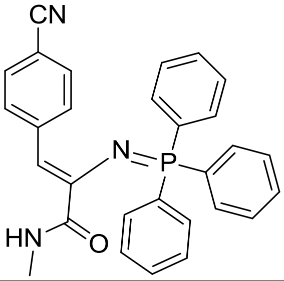	0
228	N 832	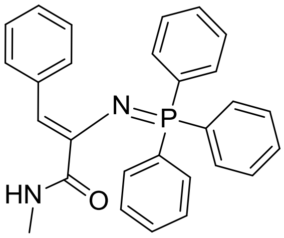	-0.5
229	N 833	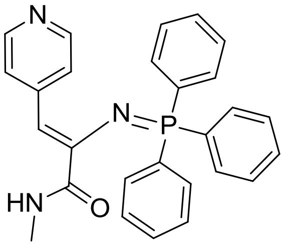	-0.6

Back to Contents

230	N 840	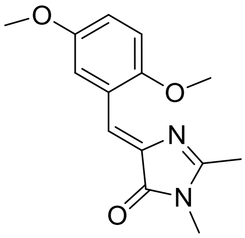	-2.5
231	N 844.1	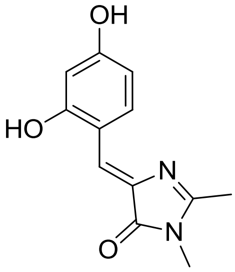	-0.1
232	N 848.1	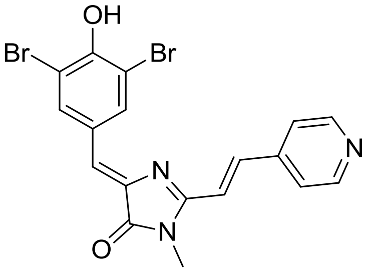	-1.8
233	N 848.2	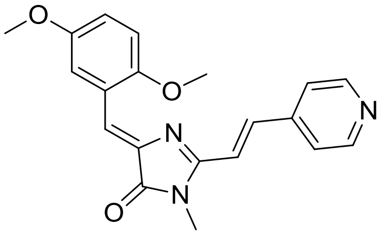	2.6
234	N 848.3	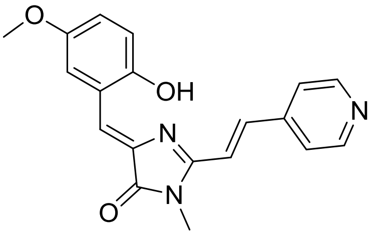	2.9
235	N 851A	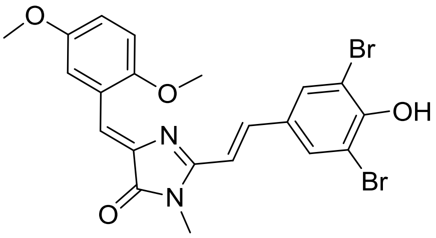	1.4
236	N 851B	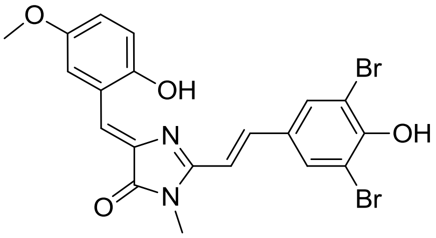	1.2

Back to Contents

237	N 853	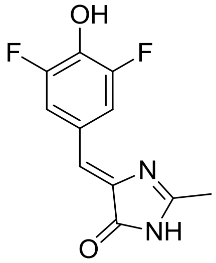	-0.9
238	N 855	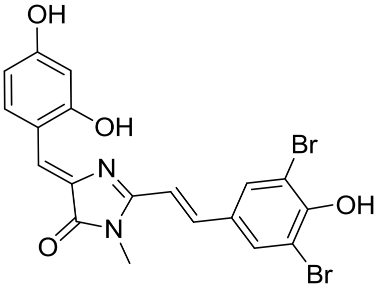	0.4
239	N 860C	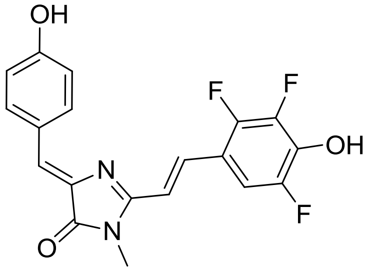	-0.2
240	N 861	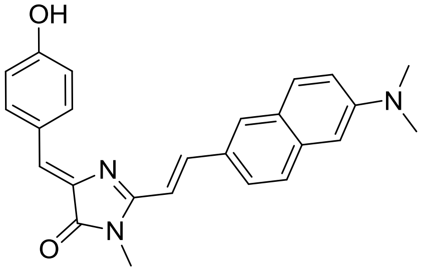	0
241	N 863	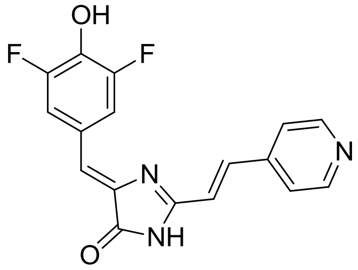	0
242	N 864	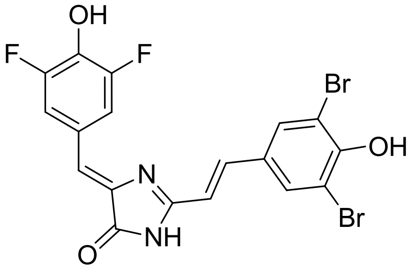	-2.3
243	N 865	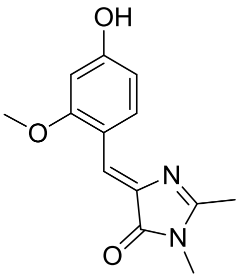	0.8

Back to Contents

244	N 866	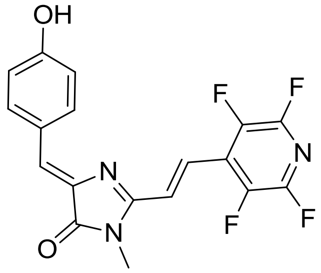	-0.2
245	N 871B	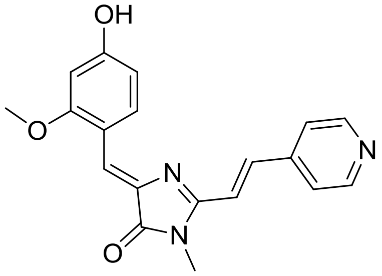	0.4
246	N 873	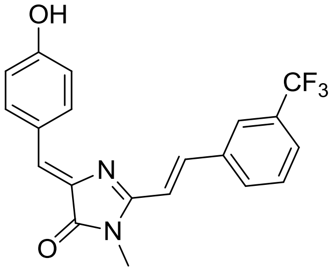	0.4
247	N 886	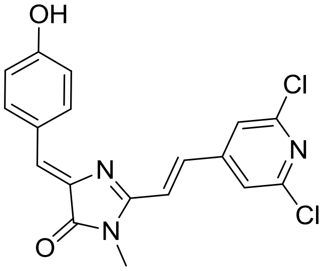	-0.2
248	N 892	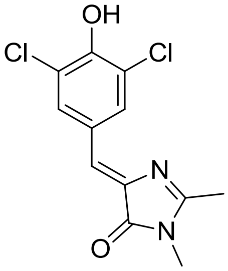	-1
249	N 893	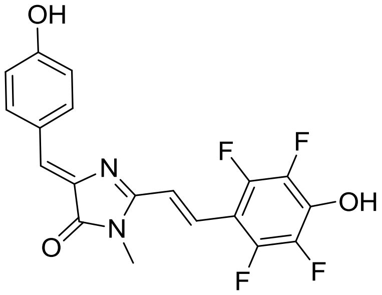	-0.4

[Back to Contents]

250	N 898	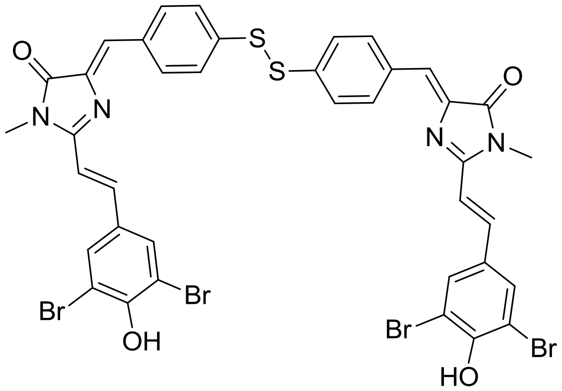	0.1
251	N 901	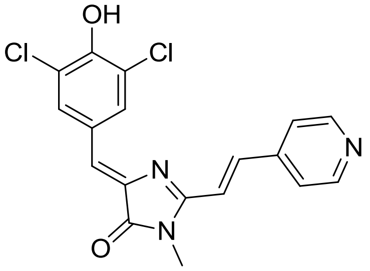	0.4
252	N 904	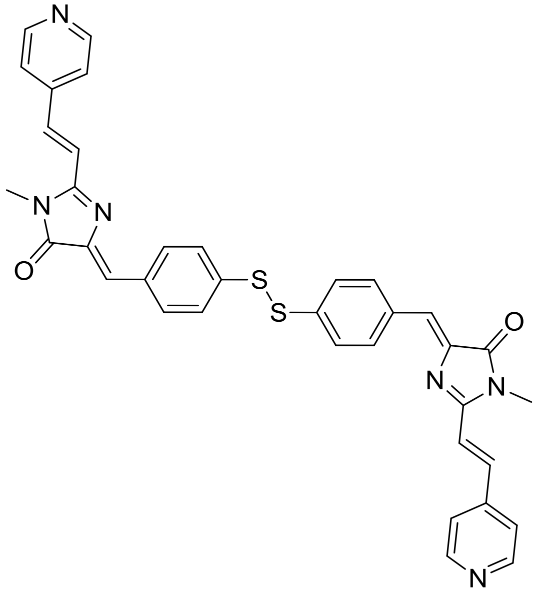	-1.2
253	N 906	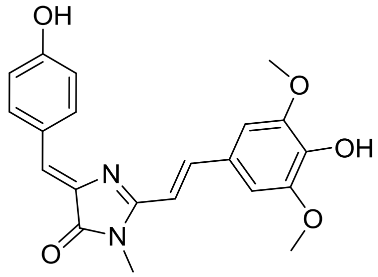	0.1
254	N 908	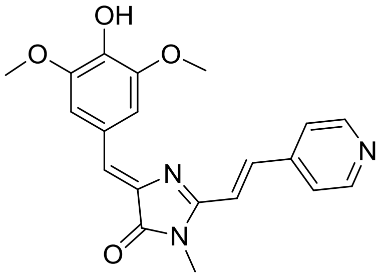	2.4

Back to Contents

255	N 914	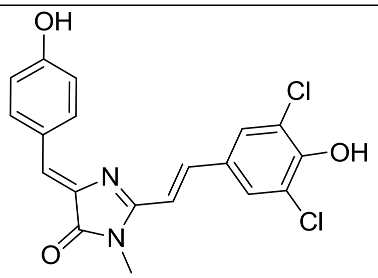	-0.8
256	N 960a	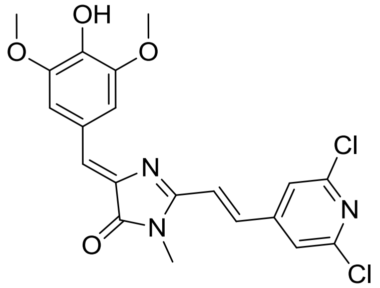	5.3
257	N 960b	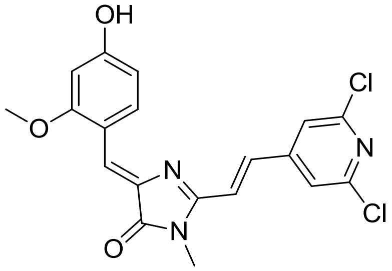	0.7
258	N 967	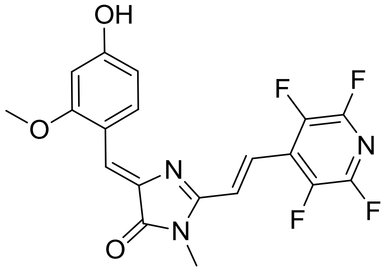	0.8
259	N 971	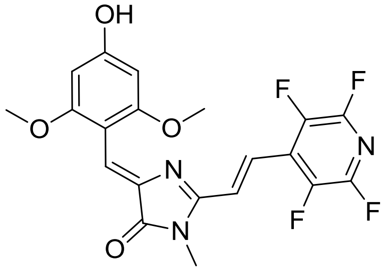	0.1
260	N 973	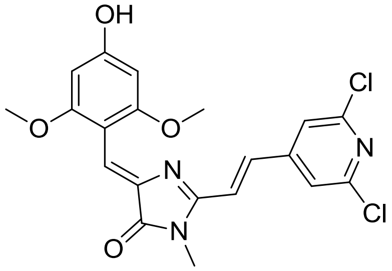	2.3
261	N 976	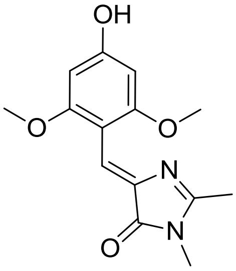	0.5

Back to Contents

262	N 979	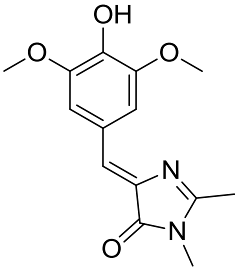	-0.2
263	N 980	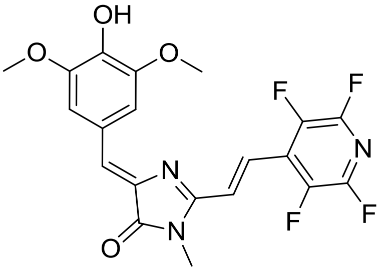	1
264	N 1019	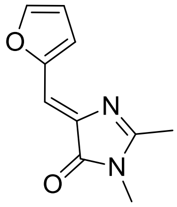	-0.3
265	N 1020	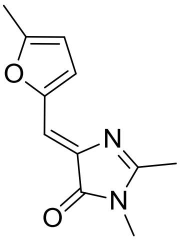	-0.2
266	N 1021	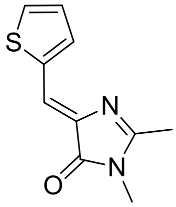	0.3
267	N 1027.1	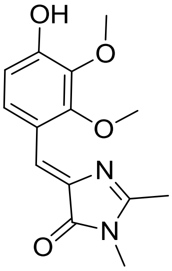	-0.1
268	N 1036	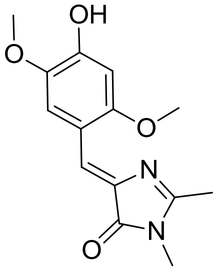	0.5

Back to Contents

269	N 1039	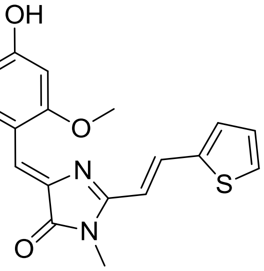	1.1
270	N 1042	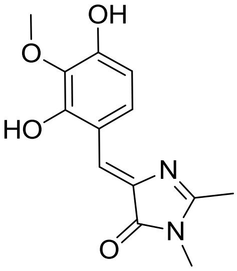	1.4
271	N 1048	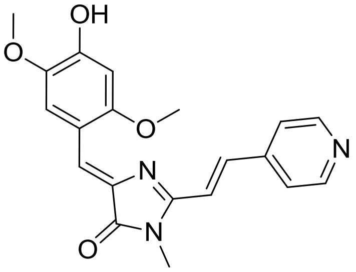	1.5
272	N 1049	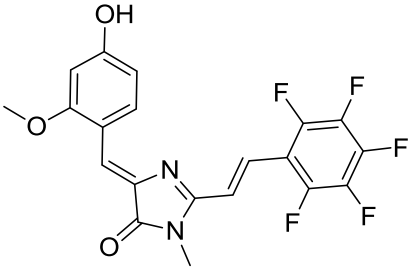	1.8
273	N 1052	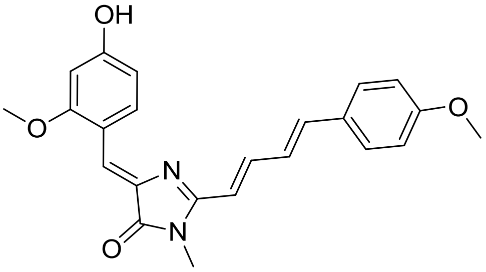	4.6
274	N 1056	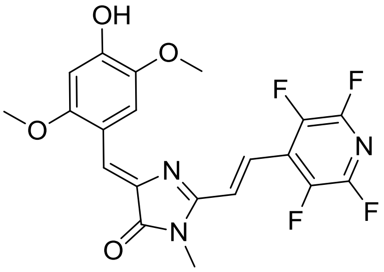	0
275	N 1068.2	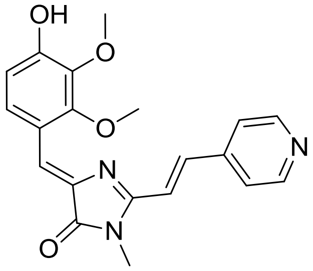	7.1

Back to Contents

276	N 1069	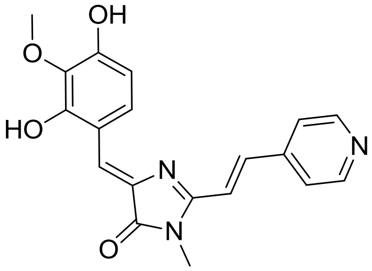	2.7
277	N 111	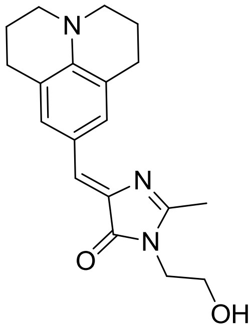	1
278	N 1118	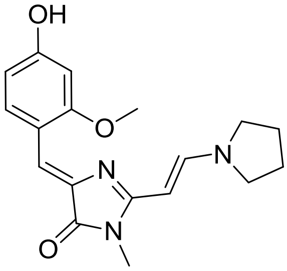	3
279	N 1122	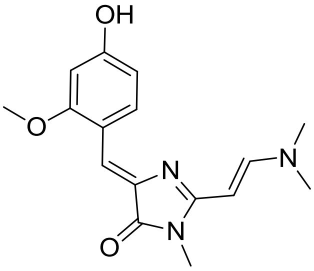	2.3
280	N 1123	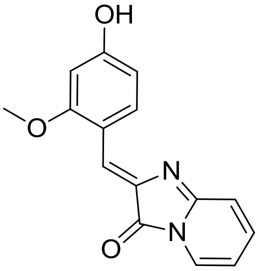	0.3
281	N 1124	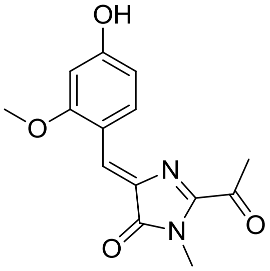	1.3

Back to Contents

282	N 1135	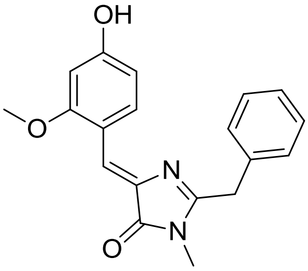	0
283	N 1139	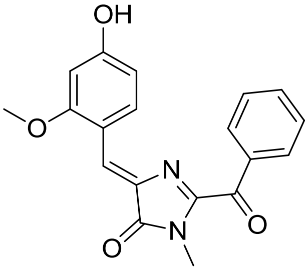	0.6
284	N 1142	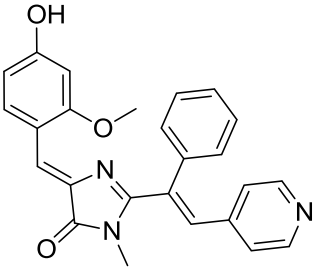	0.1
285	SA 33	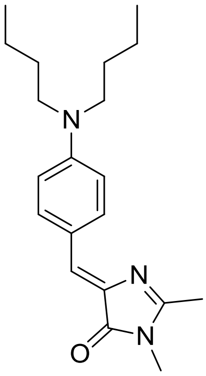	0.2
286	SA 49.1	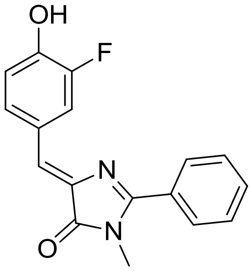	0
287	SA 99	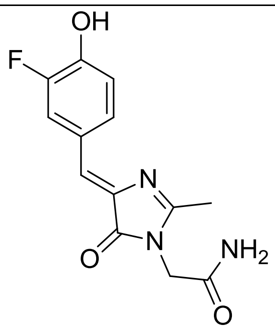	0.1

Back to Contents

288	SA 102	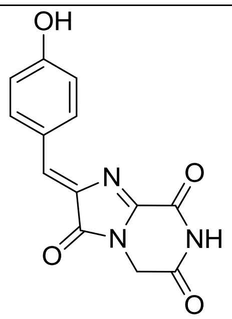	0.2
289	SA 108	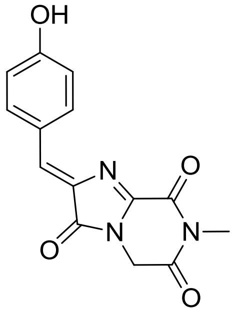	-0.1
290	SA 111	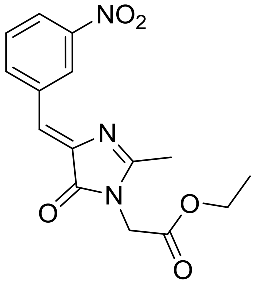	-0.1
291	SA 113	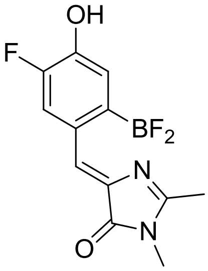	0.1
292	SA 117	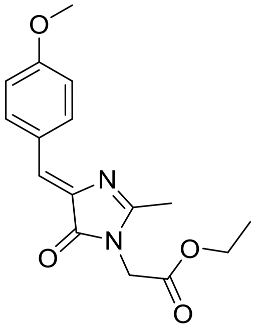	-0.2
293	SA 120	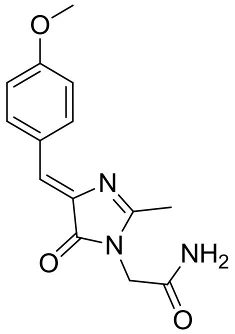	-2.1

Back to Contents

294	SA 128	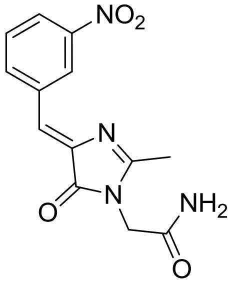	0.1
295	SA 130	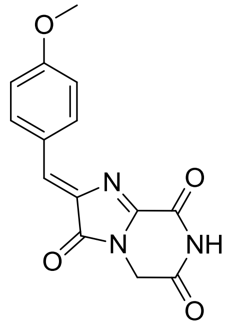	0.4
296	SA 148	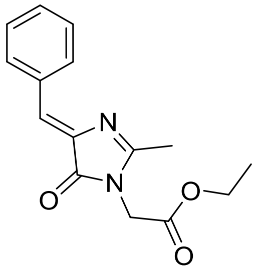	-1.3
297	SA 152	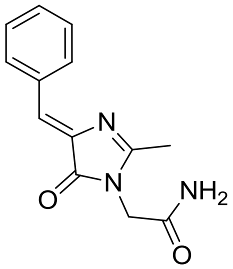	-0.3
298	SA 158	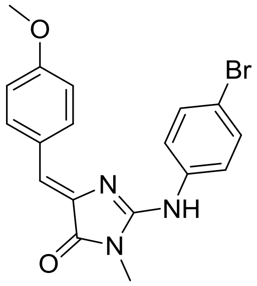	0.2
299	SA 160.1	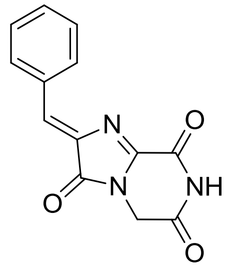	0.1

Back to Contents

300	SA 168	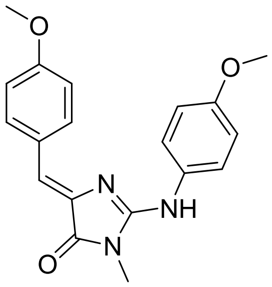	-0.4
301	SA 179	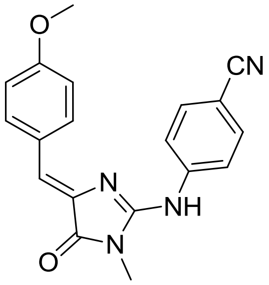	0.7
302	SA 198	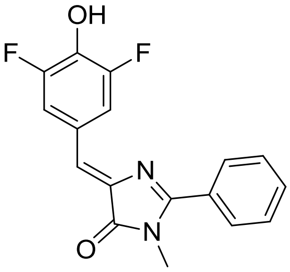	-0.5
303	SA 300A	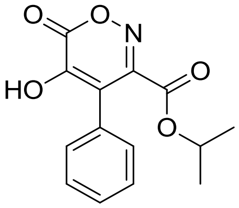	0.1
304	SA 300B	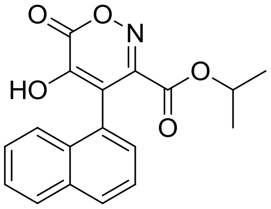	-0.9
305	SA 300D	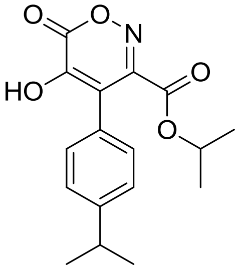	-1.2
306	SA 305A	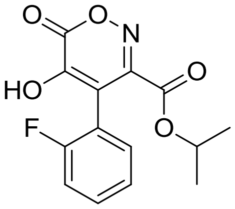	-0.7

Back to Contents

307	SA 305B	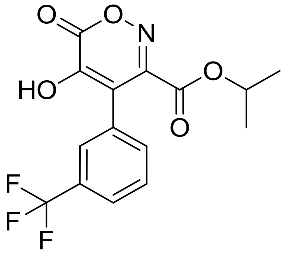	-0.5
308	SA 305C	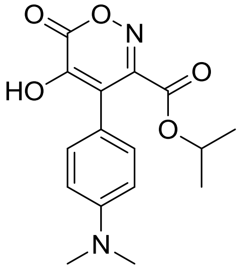	-0.6
309	SA 306B	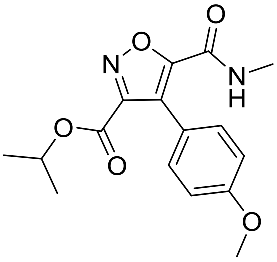	-0.3
310	SA 307A	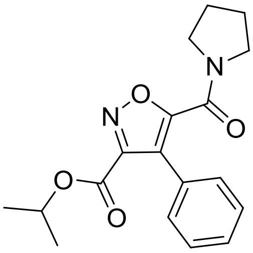	-0.3
311	SA 309	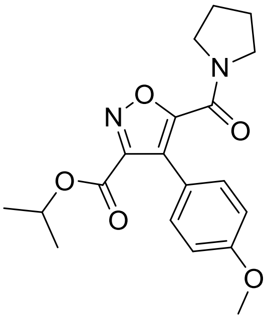	-0.5
312	SA 325A	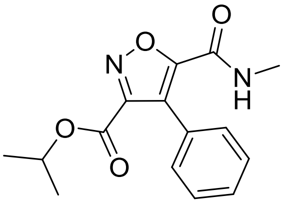	-0.5
313	SA 325E	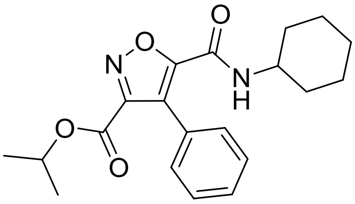	-0.6

[Back to Contents](#)

314	SA 330A	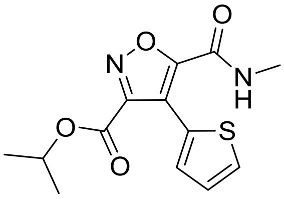	-0.5
315	SA 330E	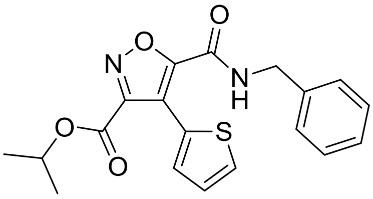	-0.7
316	SA 331A	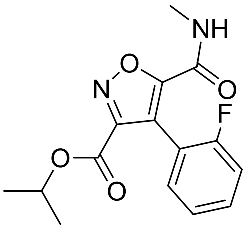	-0.7
317	SA 331D	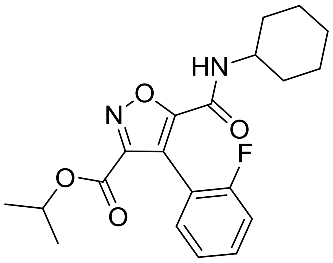	-0.7
318	SA 331E	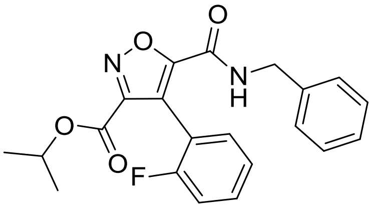	-0.9
319	SA 332.1	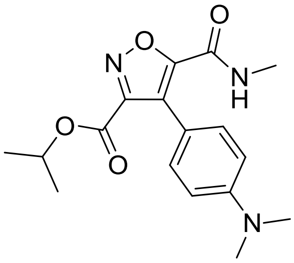	-0.4
320	SA 332.2	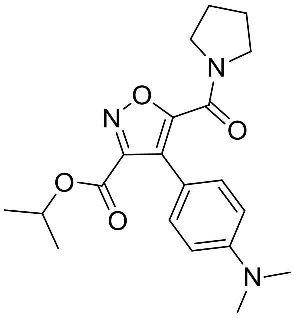	-0.4

Back to Contents

321	SA 332E1	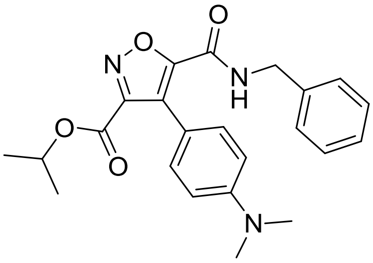	-0.6
322	SA 333	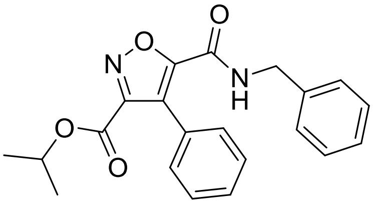	-0.4
323	SA 341A	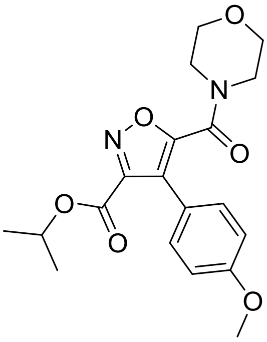	-0.7
324	SA 341B	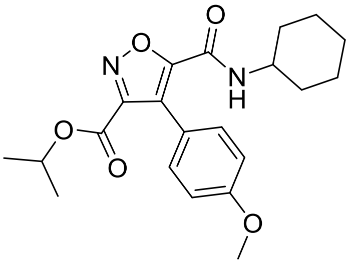	-0.2
325	SA 341C	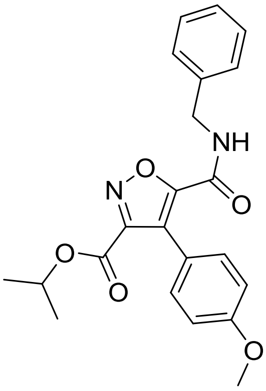	-0.2
326	SA 342A	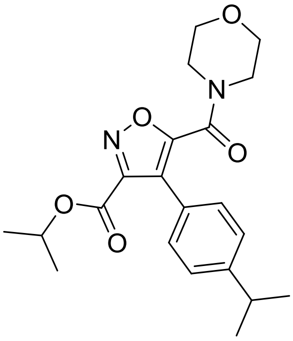	-0.3

Back to Contents

327	SA 342B	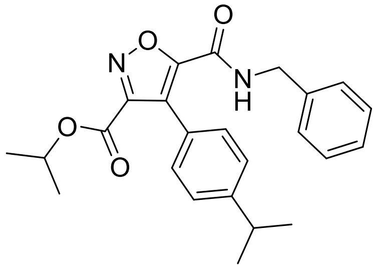	-0.5
328	SA 343A	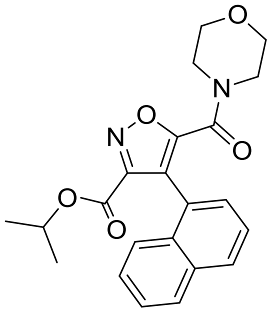	-0.4
329	SA 343B	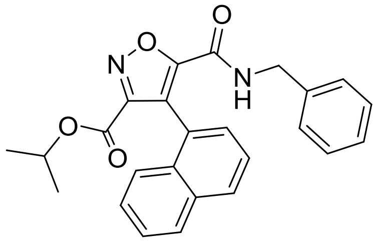	-0.9
330	SA 344A	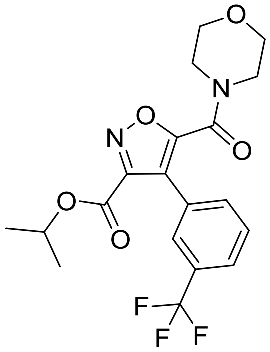	-0.5
331	SA 344B	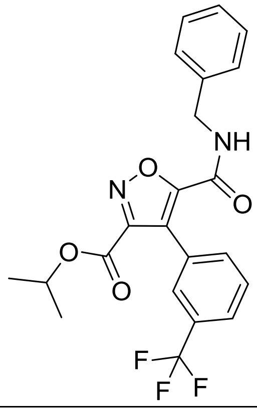	-0.7

Back to Contents

332	SA 345B	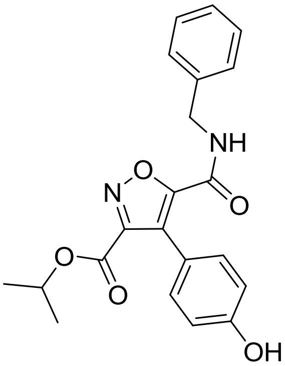	-0.8
333	SA 350C	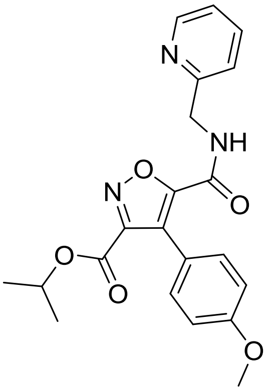	-0.6
334	SA 375	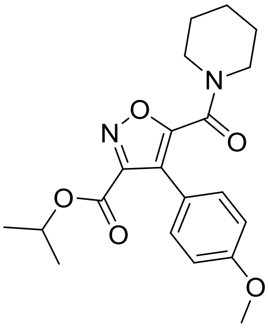	-0.4
335	SA 400	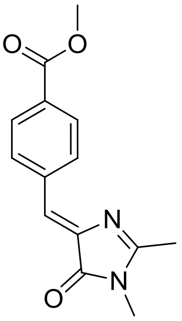	0
336	SA 410	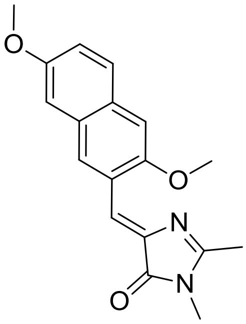	-0.2

Back to Contents

337	SA 412	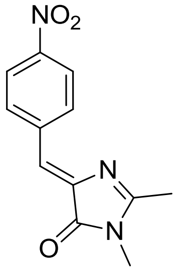	0
338	SA 433	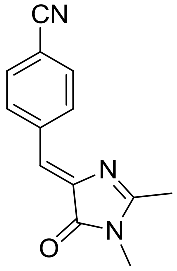	-0.4
339	SA 471	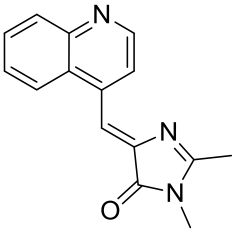	-0.1
340	SA 474.1	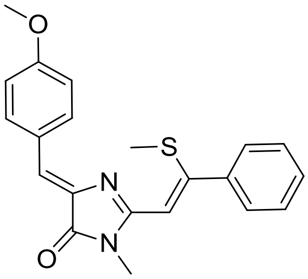	0
341	SA 497	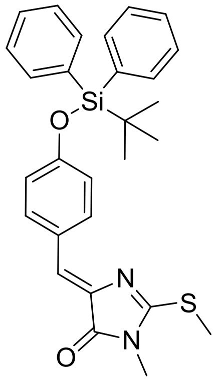	-0.4
342	SA 561A	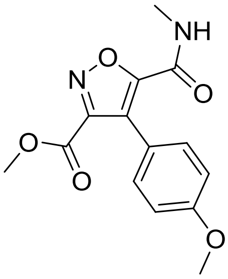	-0.3

[Back to Contents]

343	SA 561B	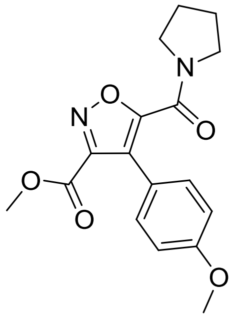	-0.6
344	SA 561C	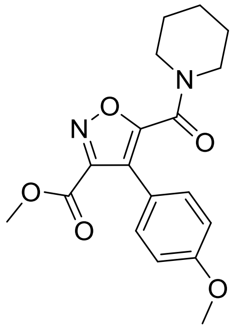	-1
345	SA 561D	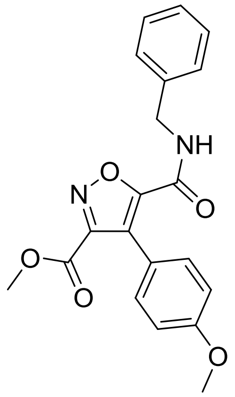	-0.4
346	SA 561E	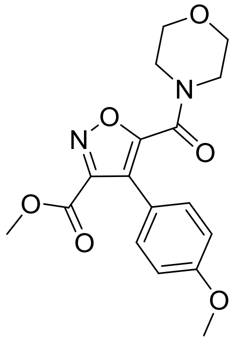	-0.7
347	SA 562B	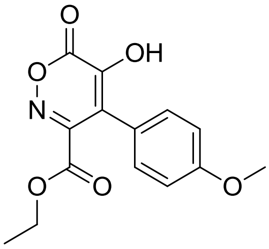	-1
348	SA 581	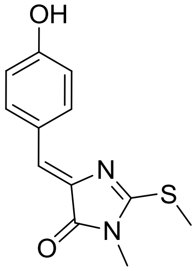	1

Back to Contents

349	SA 599.21	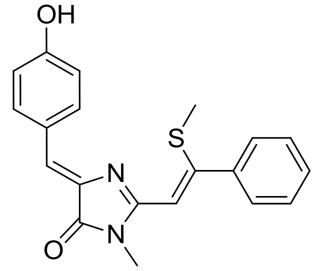	-0.3
350	SAI 32	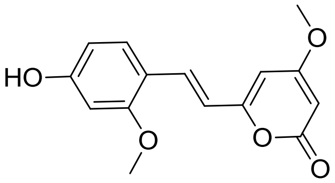	0.4
351	SAI 89	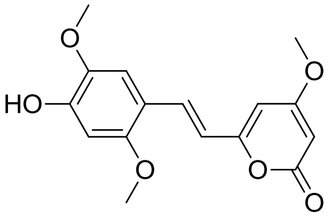	1.1
352	SAI 112	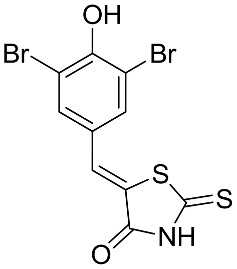	0.3
353	SAI 115	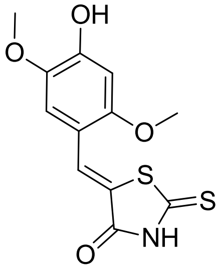	-0.3
354	SAI 117	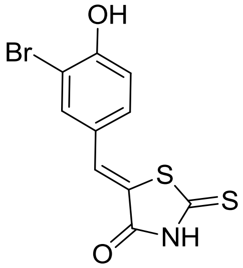	-0.2
355	SAI 118	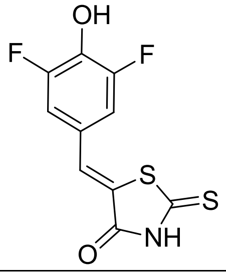	0

Back to Contents

356	SAI 120	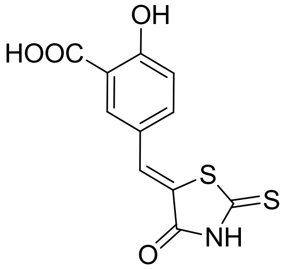	0.6
357	SAI 121	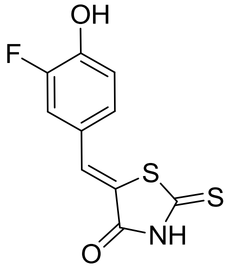	0.9
358	SAI 122	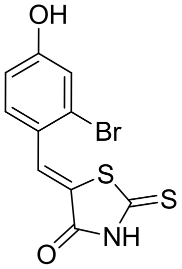	0.1
359	SAI 125	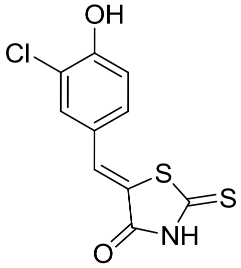	0.6
360	SAI 127	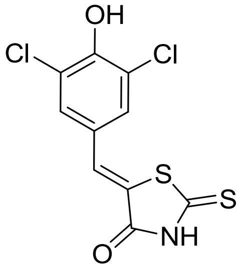	-0.2
361	SH 016.3B	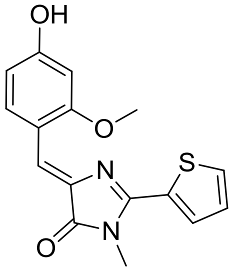	0.9
362	SV 11	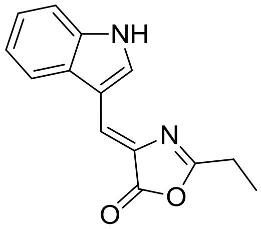	0

Back to Contents

363	SV 13	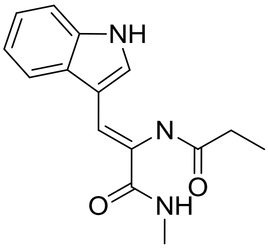	-0.1
364	SV 14	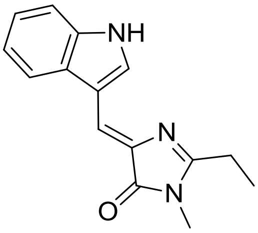	0
365	SV 18	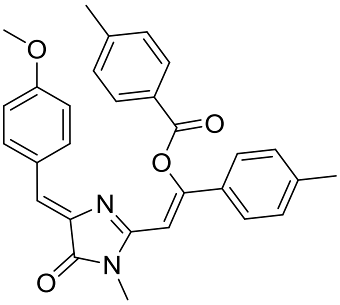	0.4
366	SV 29	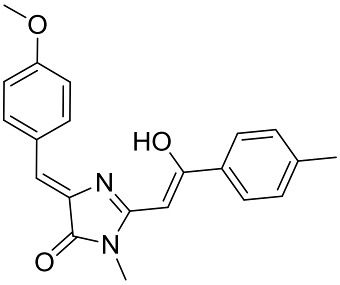	-0.2
367	SV 30	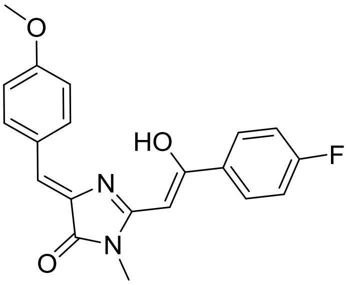	-0.4
368	SV 50.10	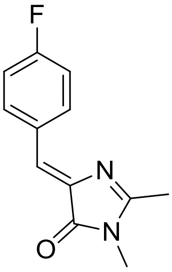	-0.2
369	SV 50.11	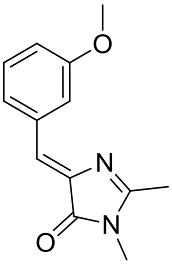	-0.2

Back to Contents

370	SV 50.9	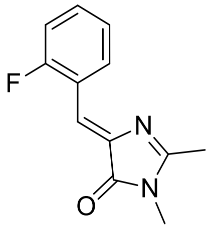	-0.2
371	SV 51.1	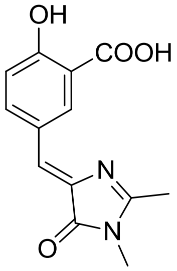	-0.4
372	SV 51.10	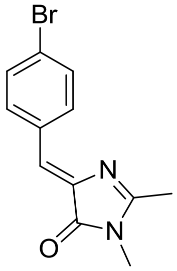	-0.2
373	SV 51.12	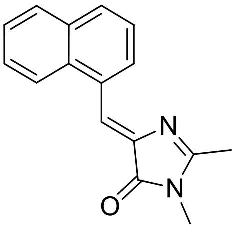	0.4
374	SV 51.5	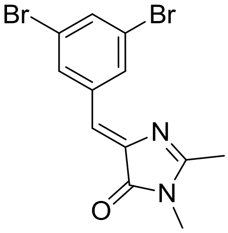	-0.3
375	SV 52.7	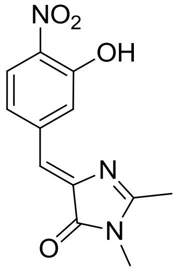	-0.2
376	SV 52.8	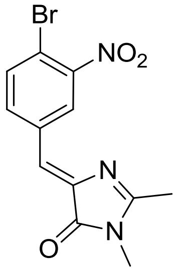	0.6

Back to Contents

377	SV 6	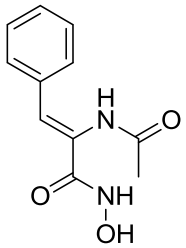	0
378	SV 7	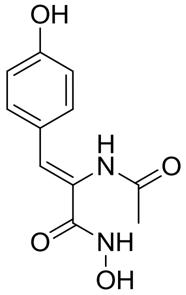	0
379	VZ 1	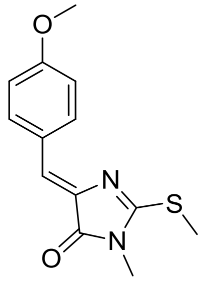	0.4
380	VZ 2	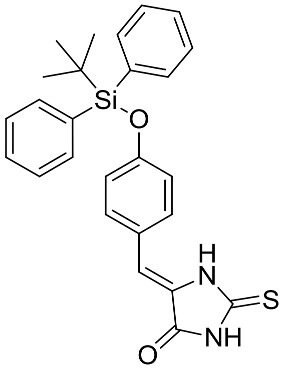	-0.2
381	X 001	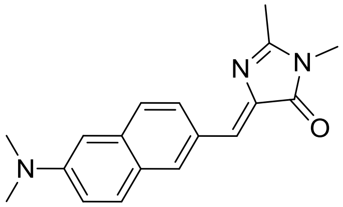	-0.5
382	X 002	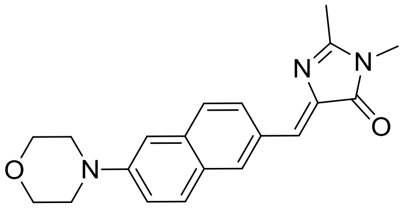	-0.6
383	X 4	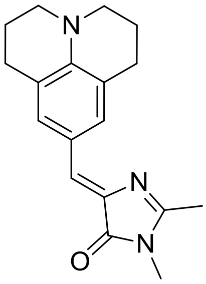	1

Back to Contents

384	X 5	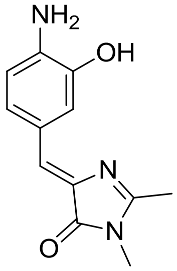	0.9
385	X 6	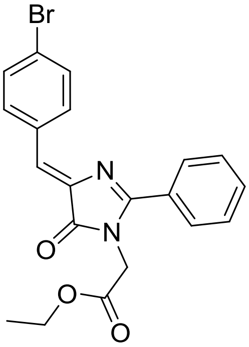	-0.1
386	X 7	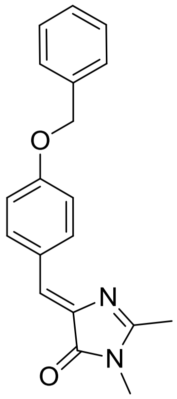	0
387	X 8	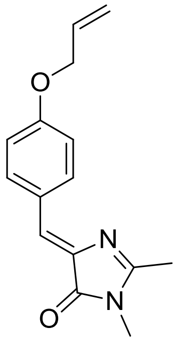	0
388	X 9	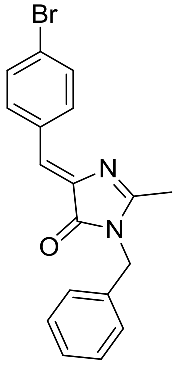	-0.6

Back to Contents

389	X 10	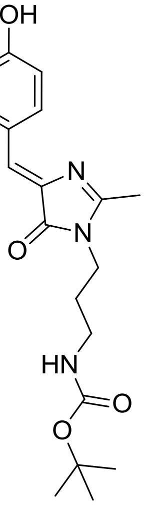	-0.2
390	X 11	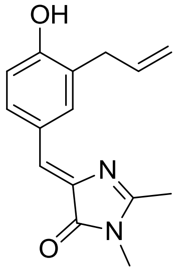	-0.2
391	X 12	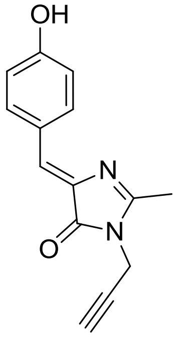	0.2
392	X 13	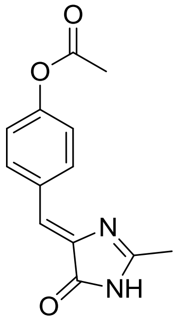	-0.8
393	X 18	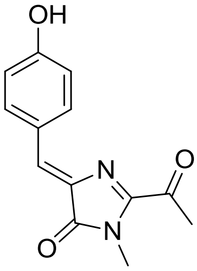	0.6

Back to Contents

394	X 20	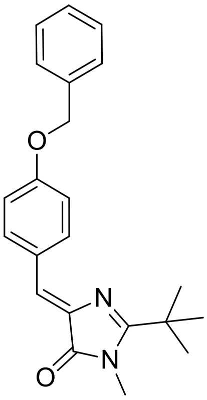	-0.2
395	X 21	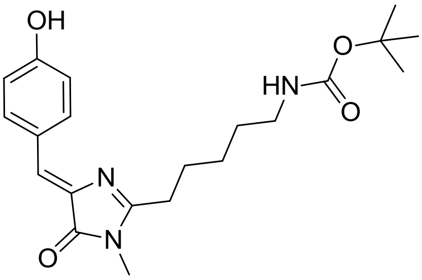	-0.1
396	X 23	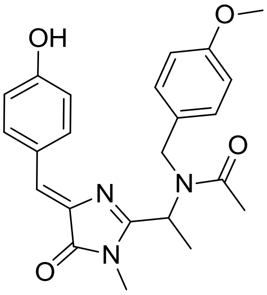	0.5
397	X 24	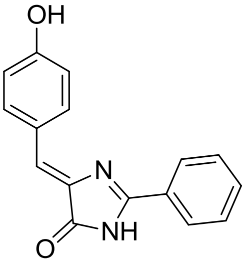	-0.5
398	X 26	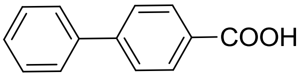	-0.1
399	X 27	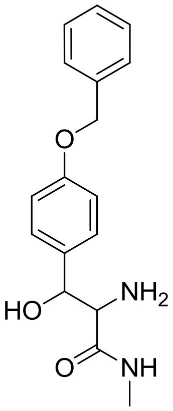	-0.1

Back to Contents

400	X 28	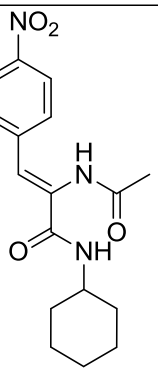	-0.3
401	X 29	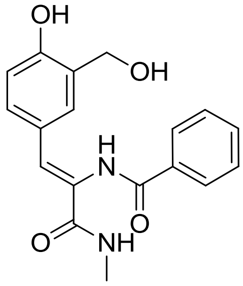	-0.6
402	ZS 62	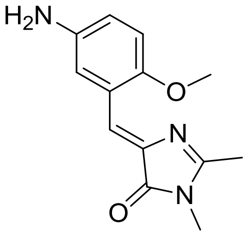	0
403	ZS 69	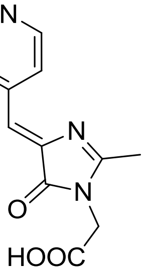	-0.4
404	ZS 99	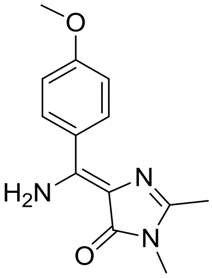	-0.2
405	ZS 109	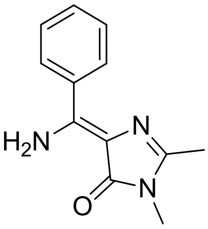	-0.7

Back to Contents

406	ZS 128	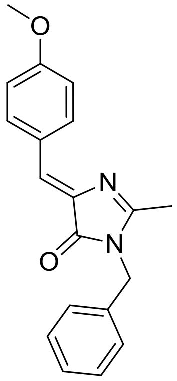	-0.3
407	ZS 129	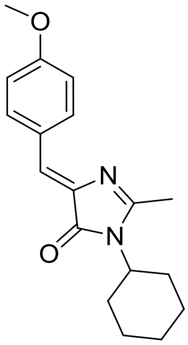	-0.2
408	ZS142	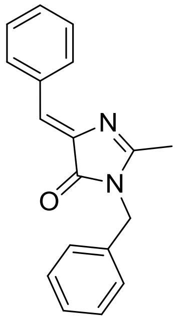	-1.4
409	ZS 143	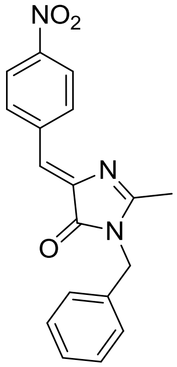	-0.9
410	ZS 146	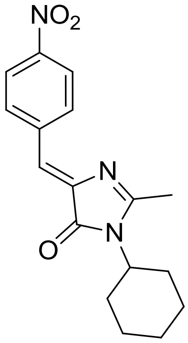	0.2

Back to Contents

411	ZS 167	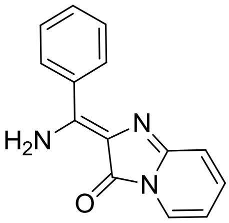	-0.1
412	ZS 174	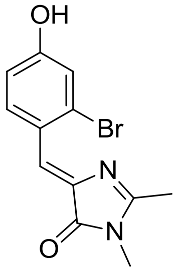	-0.3
413	ZS 178	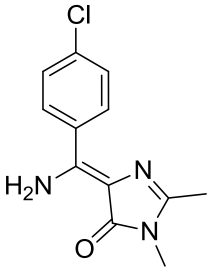	-0.3
414	ZS 182	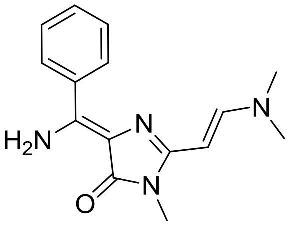	0
415	ZS 190	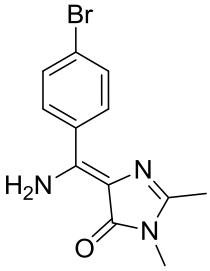	-0.5
416	ZS 199	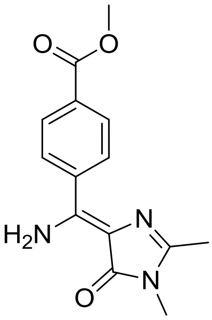	-0.4
417	ZS 228	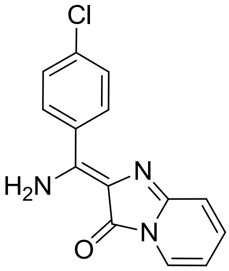	0.3

[Back to Contents](#)

418	ZS 229	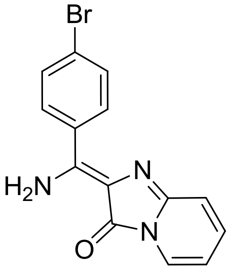	0.3
419	ZS 233	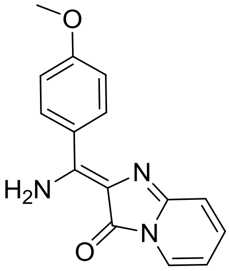	-0.1
420	ZS 234	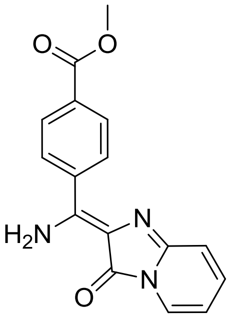	0
421	ZS 235	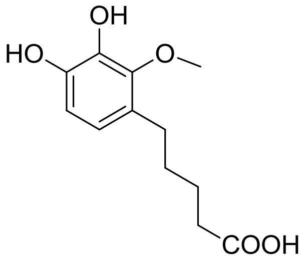	0.1
422	ZS 236	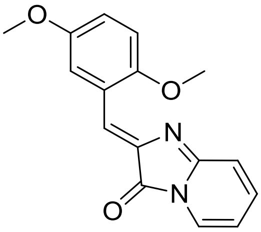	-1.2
423	ZS 256	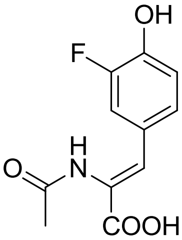	0.2
424	ZS 260.1	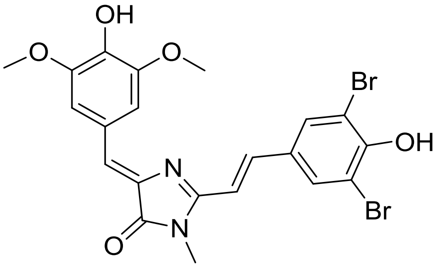	7.8

Back to Contents

425	ZS 285b	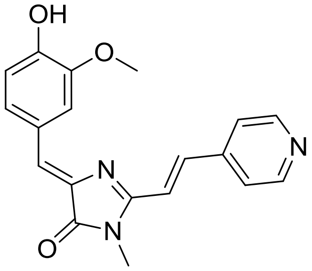	0.5
426	ZS 286	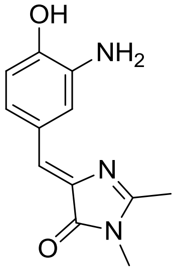	0.3
427	ZS 289a	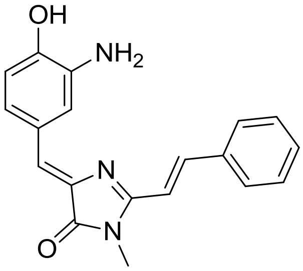	-0.1
428	ZS 291	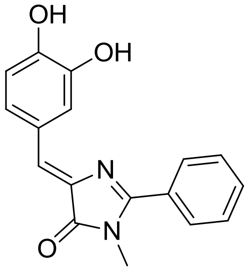	0.4
429	ZS 292	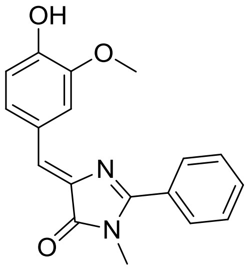	-0.2
430	ZS 295	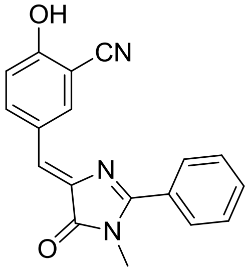	0
431	ZS 297	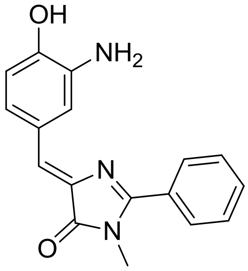	0.1

[Back to Contents]

432	ZS 298	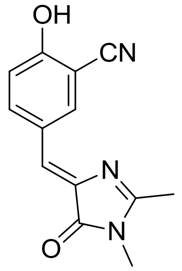	-0.2
433	ZS 300a	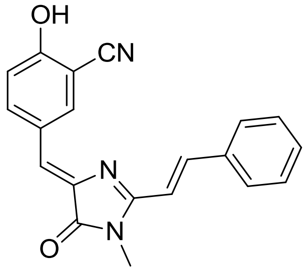	-0.1
434	ZS 309	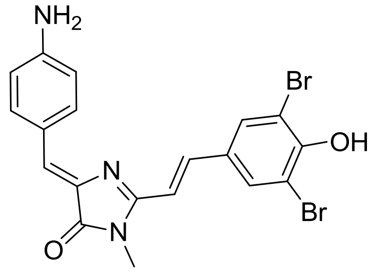	0.3
435	ZS 316	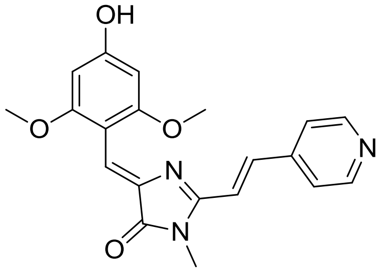	0.4
436	ZS 319.2	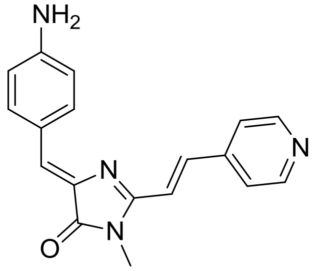	2
437	ZS 321	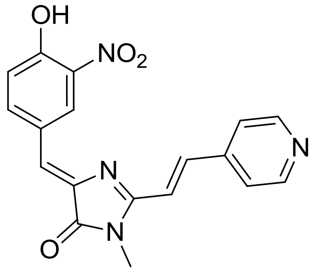	1.3
438	ZS 325	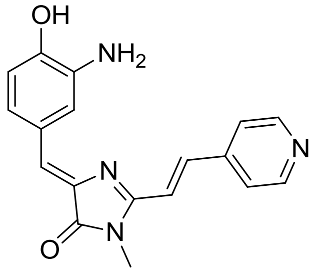	1.6

Back to Contents

439	ZS 331	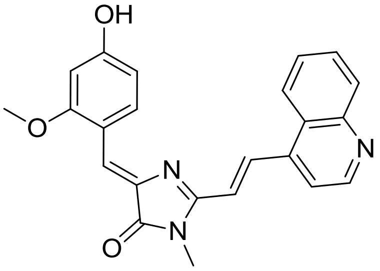	3.3
440	ZS 362	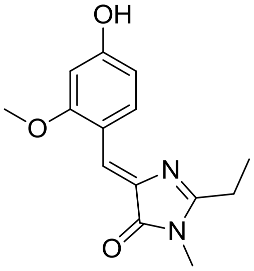	0.2

[a] - $\Delta T_m = T_m^{LTR\text{-}III+ligand} - T_m^{LTR\text{-}III}$

Back to Contents

Table S2. Comparison of stabilization of model G4s and double stranded control sequence by primary screening lead compounds.

ΔT_m [a]	LTR III	Telo	Pu39	Duplex
ZS 260.1	7.8±0.3	0.7±0.2	0.9±0.6	0.1±0.09
N 1068.2	7.1±0.3	1.2±0.2	0±0.4	0.1±0.04
N 960a	5.3±0.2	1±0.7	0.5±0.01	-0.5±0.07
N 683.1	5±0.1	-0.1±0.04	1.4±0.6	0±0.04
N 1052	4.6±0.2	3.9±0.1	2.1±0.2	-0.1±0.04
ZS 320	3.6±0.2	0.6±0.1	0±0.3	-0.1±0.07
ZS 331	3.3±0.2	2.5±0.3	0.8±0.2	-0.2±0.1
AL 267	3±0.2	0.5±0.2	0.2±0.2	0.2±0.04
M 813	2.9±0.2	-0.6±0.9	-1.5±1	0±0.04
MID 155	2.9±0.3	0.1±0.04	0±0.04	-0.1±0.04
N 848.3	2.9±0.1	1.1±0.2	0.7±0.3	0.1±0.09
M 903.2	2.8±0.3	0.4±0.1	-1±0.5	0.1±0.07
N 848.2	2.6±0.2	2.1±0.01	0.1±0.4	0.4±0.04
N 908	2.4±0.2	0.1±0.1	-0.7±0.2	0±0.04

[a] - $\Delta T_m = T_m^{\text{DNA target+ligand}} - T_m^{\text{DNA target}}$

Back to Contents

Table S3. Primary screening of newly synthesized GFP chromophore analogs for the stabilizing properties of FAM/TAMRA-labeled LTR-III G-quadruplex.

No	code	Structure	LTR-III ΔT_m [a]
441	N1193	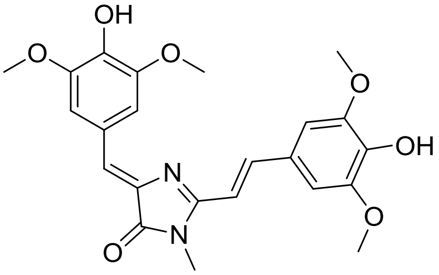	14
442	N1195	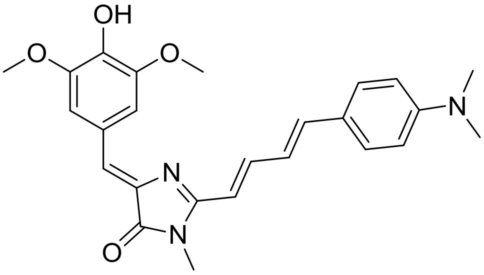	4.2
443	N1196	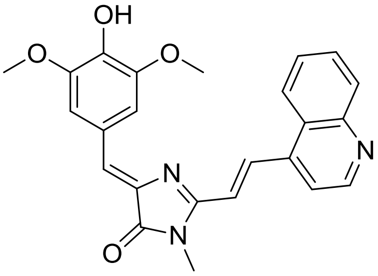	11.7
444	N1197	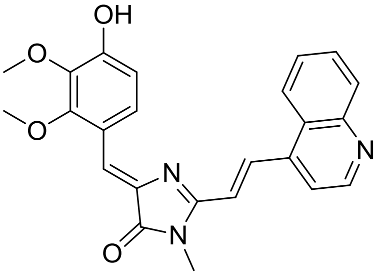	11.3
445	N1198	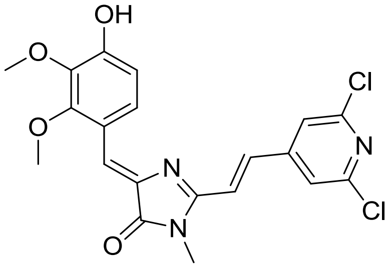	6.5
446	N1199	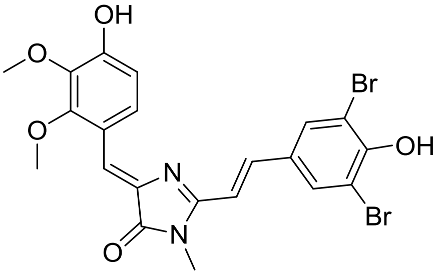	4.3

Back to Contents

447	AR556	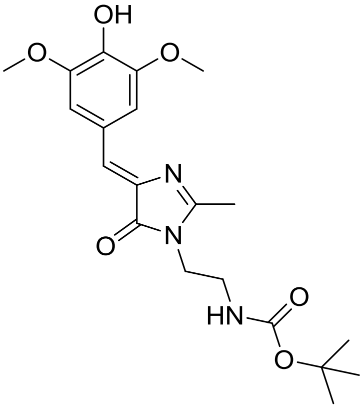	-1.1
448	AR558	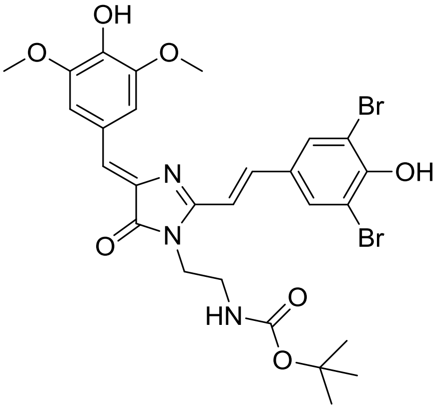	0.7
449	AR559	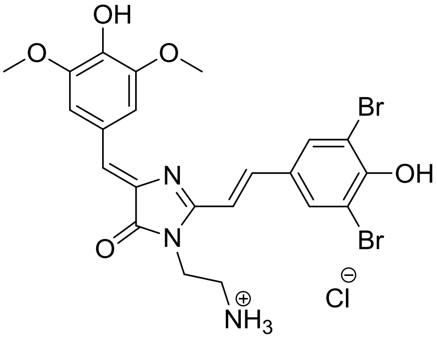	1.2

[a] - $\Delta T_m = T_m^{LTR\text{-}III+ligand} - T_m^{LTR\text{-}III}$

Back to Contents

Supplement S4. Structural motifs of the lead compounds in comparison with the other structurally related compounds.

Figure S4-1. The comparison of the substituents at 5-position of the GFP chromophore core, ΔT_m values are shown under the chemical formulas

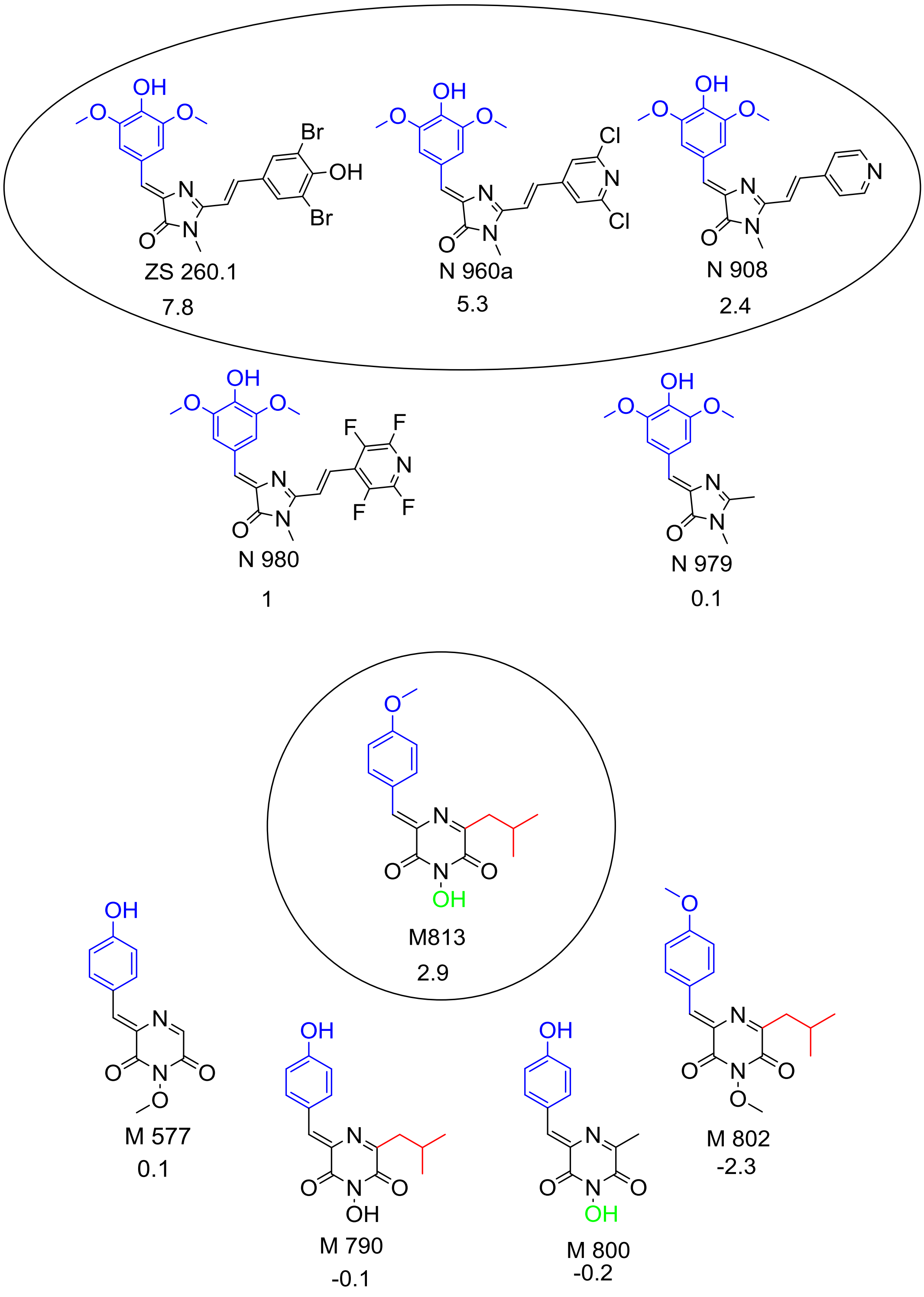

[Back to Contents](#)

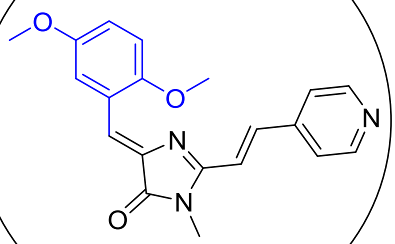

N848.2
2.6

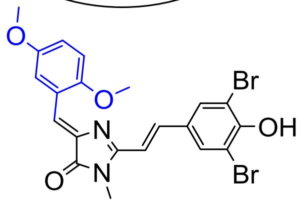

N 851A

1.4

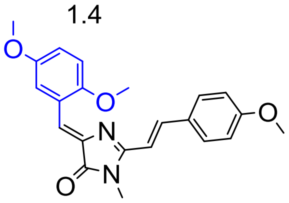

Al 118
-0.1

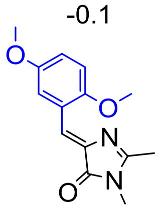

N 840
-2.5

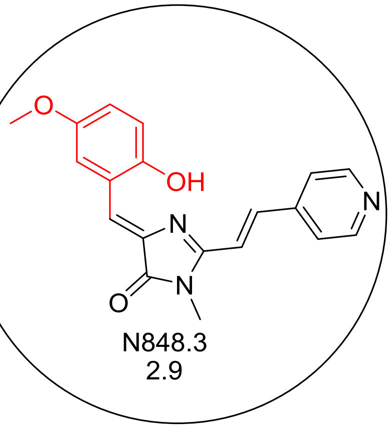

N848.3
2.9

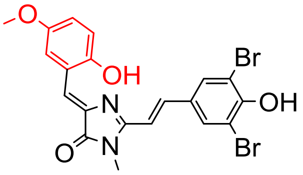

N 851B
1.2

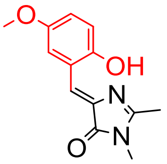

M 2360
-0.3

Back to Contents

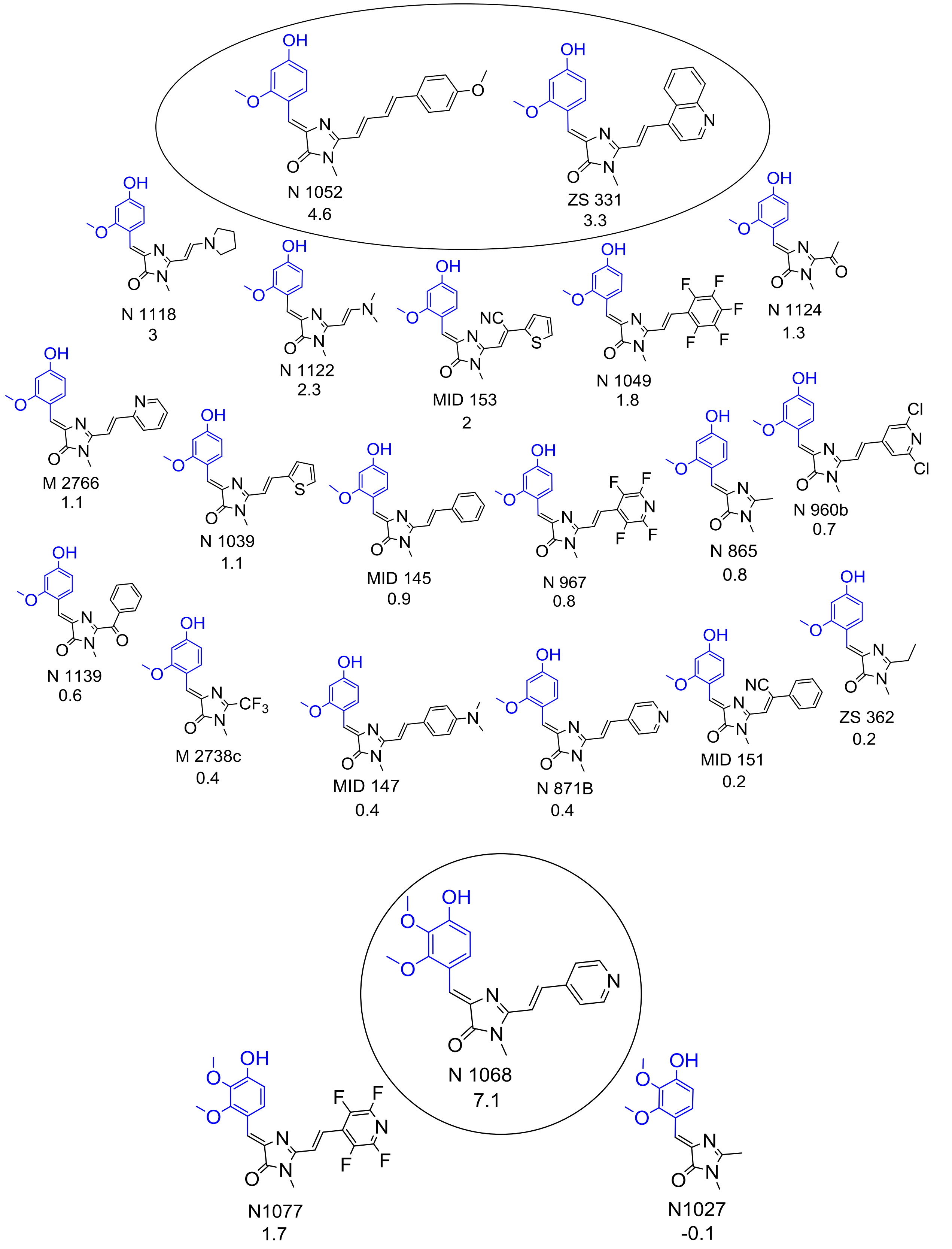

Back to Contents

Figure S4-2 The comparison of the substituents at 2-position of the GFP chromophore core, ΔT_m values are shown under the chemical formulas

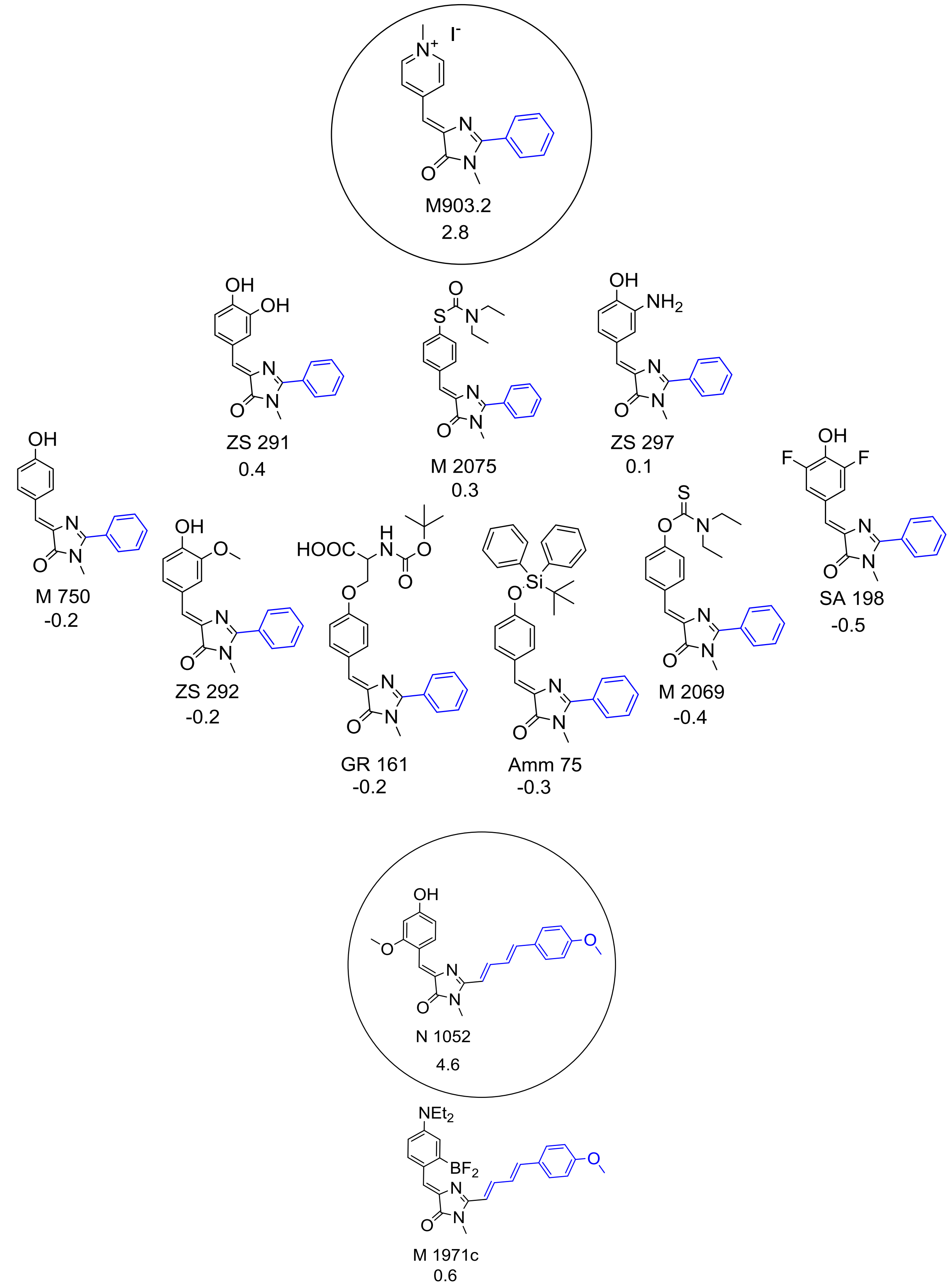

Back to Contents

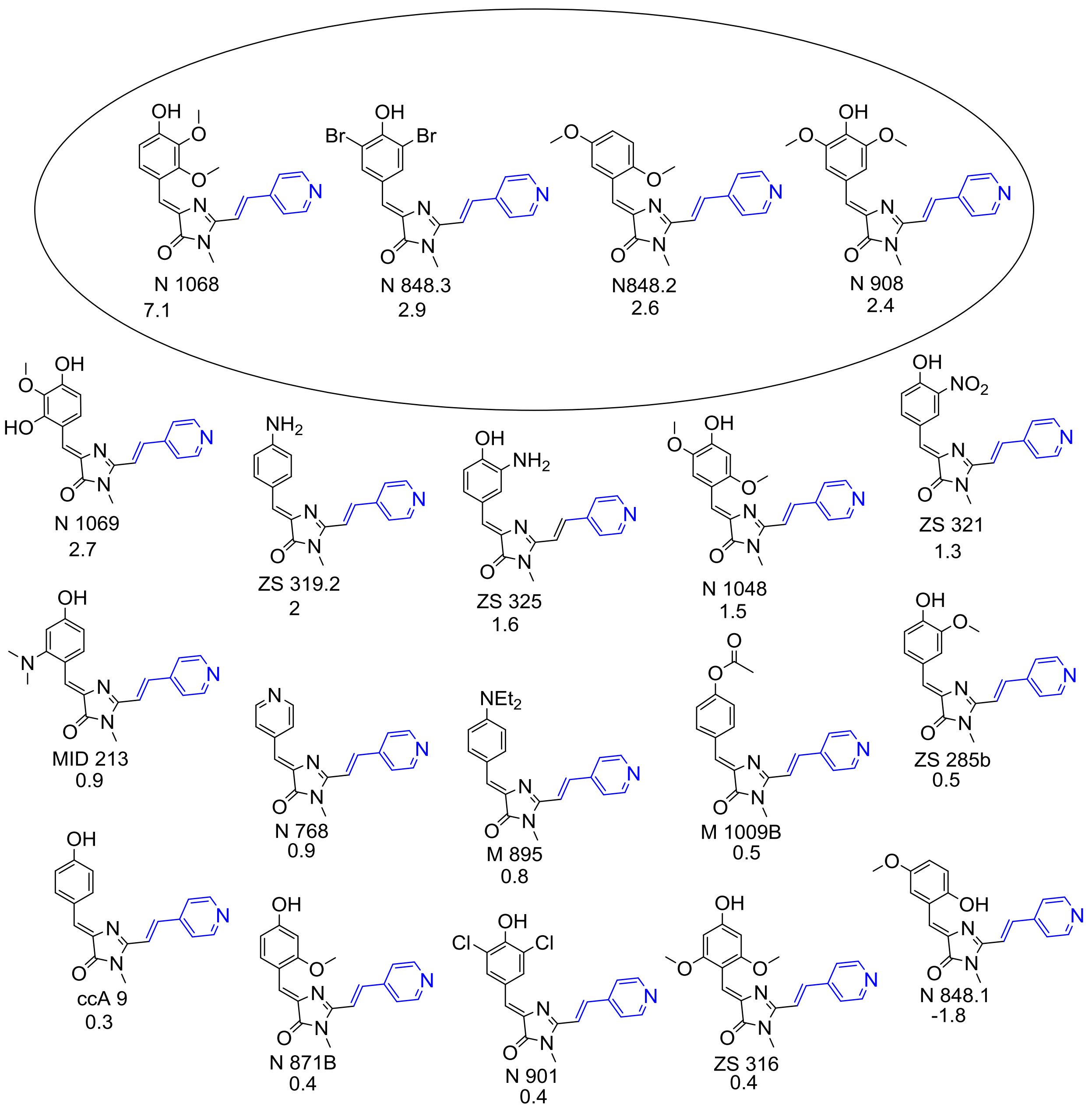

Back to Contents

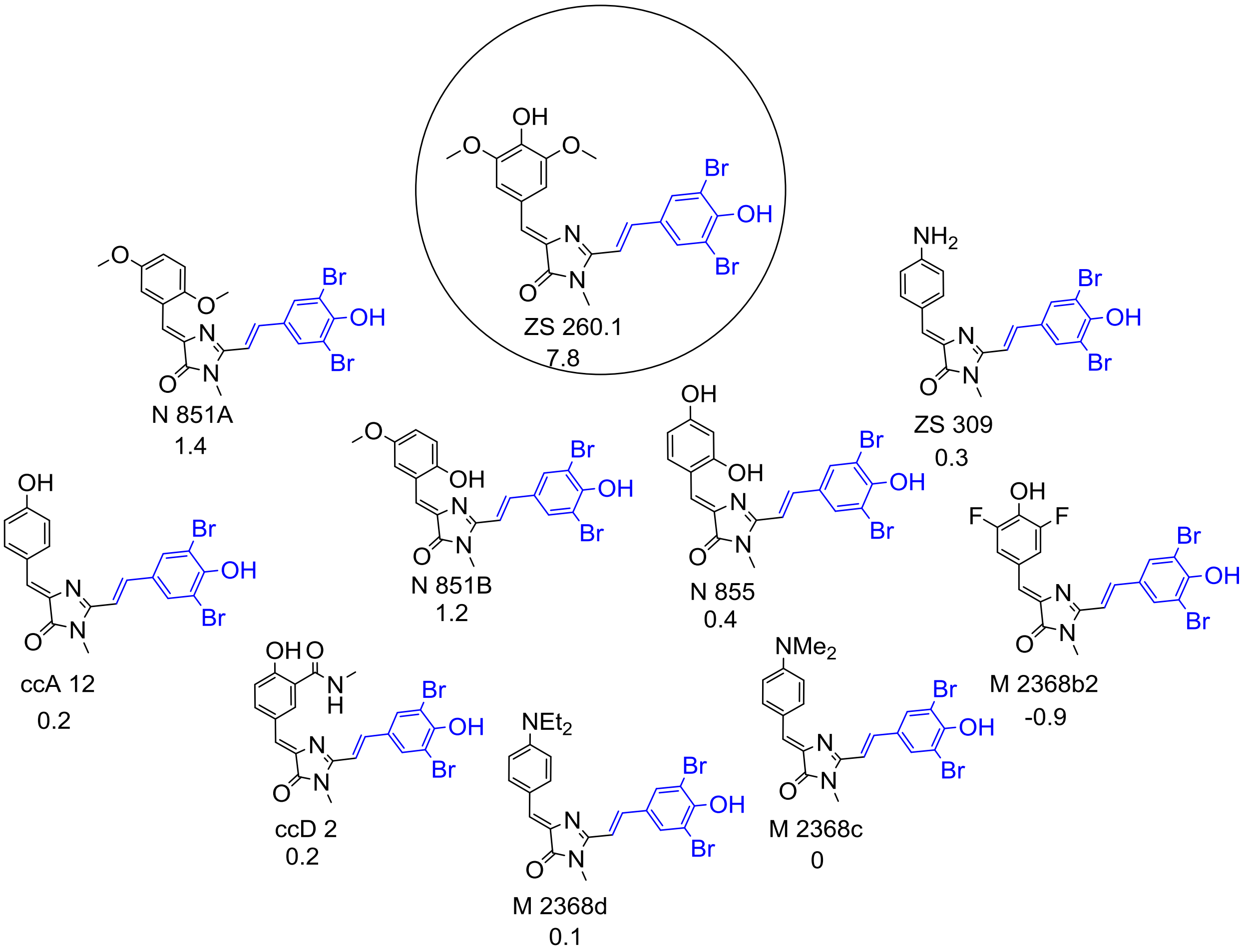

Back to Contents

Table S5. Comparison of stabilization of model G4s and double stranded control sequences by newly synthesized compounds.

ΔT_m [a]	LTR III	Telo	Pu39	Duplex
N1193	14±0.5	9.5±0.1	3.8±0.04	0.6±0.1
N1196	11.7±0.5	8.7±0.2	7.2±0.4	0.5±0.1
N1197	11.3±0.2	4.6±0.2	7±0.7	-0.4±0.2
N1198	6.5±0.3	0.9±0.2	0.1±0.1	0±0.1
N1199	4.3±0.2	0.9±0.1	0±0.2	0±0.1
N1195	4.2±0.1	0.9±0.3	1.8±0.1	0.1±0.1

[a] - $\Delta T_m = T_m^{\text{DNA target+ligand}} - T_m^{\text{DNA target}}$

Back to Contents

Table S6. Titration experiments for N1193, N1196, N1197 at various DNA:ligand molar ratios

Ligand	LTR-III G4/ligand molar ratio		
	1:50	1:20	1:2
N1193	13.1±0.2[a]	9.1±0.6	2.4±0.6
N1196	11.3±0.3	7.9±0.4	2±1
N1197	11.4±0.2	8.5±0.3	2.3±0.5

[a] - $\Delta T_m = T_m^{LTR\text{-}III+ligand} - T_m^{LTR\text{-}III}$

Back to Contents

Supplement S7. Synthetic procedures for the synthesized GFP chromophore analogues

1. General

Commercially available reagents were used without additional purification. E. Merck Kieselgel 60 was used for column chromatography. Thin layer chromatography (TLC) was performed on silica gel 60 F_{254} glass-backed plates (MERCK). Visualization was effected by UV light (254 or 312nm) and staining with $KMnO_4$.

NMR spectra were recorded on a 700 MHz Bruker Avance III NMR at 303 K and 800 MHz Bruker Avance III NMR at 333 K. Chemical shifts are reported relative to residue peaks of DMSO-d_6 (2.51 ppm for ^1H and 39.5 ppm for ^{13}C). Melting points were measured on a SMP 30 apparatus. High-resolution mass spectra (HRMS) spectra were recorded on a LTQ Orbitrap Elite (ThermoScientific) using electrospray ionization (ESI). The measurements were done in a positive ion mode (interface capillary voltage –5000 V) or in a negative ion mode (3500 V); interface temperature was set at 275 °C.

Back to Contents

2. Synthetic procedures

2.1 Preparation of 5-(Z)-benzylidene-2-methyl-3-alkyl/aryl-3,5-dihydro-4H-imidazol-4-ones

General method was reported previously.[1]

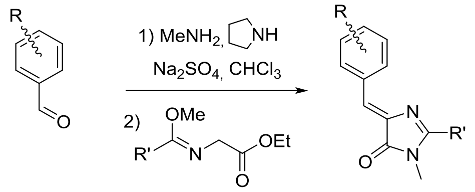

The corresponding aromatic aldehyde (10 mmol) was dissolved in $CHCl_3$ (50 mL) and mixed with methylamine solution (40% aqueous, 2.5 mL), pyrrolidine (7 mg, 0.1 mmol) and anhydrous Na_2SO_4 (10 g). The mixture was stirred for 48 h at room temperature, filtered and dried over the additional Na_2SO_4. The solvent was evaporated and corresponding ethyl((methoxy)amino)acetate (20 mmol) was added to the residue (5-10 mL of methanol was also added if it wouldn't mixed). The mixture was stirred for 24 h at room temperature, solvents were evaporated and the product was purified by column chromatography ($CHCl_3$-EtOH).

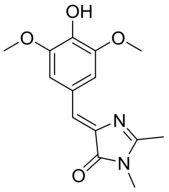

(*Z*)-5-(4-hydroxy-3,5-dimethoxybenzylidene)-2,3-dimethyl-3,5-dihydro-4*H*-imidazol-4-one (N 979)

^{1}H NMR (300 MHz, DMSO-d_6) δ ppm 9.15 (br. s., 1 H), 7.63 (s, 2 H), 6.90 (s, 1 H), 3.79 (s, 6 H), 3.08 (s, 3 H), 2.33 (s, 3 H). Previously characterized.[2]

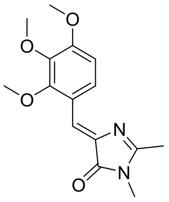

(*Z*)-5-(2,3,4-trimethoxybenzylidene)-2,3-dimethyl-3,5-dihydro-4*H*-imidazol-4-one (N 1043)

Yellow solid (1.86 g, 64%); mp = 192-195 ºC; ^{1}H NMR (700 MHz, DMSO-d_6) δ ppm 8.53 (d, *J*=9.0 Hz, 1 H), 7.13 (s, 1 H), 6.95 (d, *J*=9.0 Hz, 1 H), 3.88-3.85 (m, 6 H), 3.77 (s, 3 H), 3.09 (s, 3 H), 2.34 (s, 3 H); ^{13}C NMR (75 MHz, DMSO-d_6) δ ppm 169.9, 163.5, 155.4, 153.3, 141.4,

---

[1] Baldridge A., Kowalik J., Tolbert L. M. *Synthesisю* **2010**, 14, 2424.
[2] Paige J.S., Wu K.Y., Jaffrey S.R., Science, **2011**, 333 (6042), 642-646.

Back to Contents

137.6, 127.6, 120.4, 118.0, 108.4, 61.7, 60.5, 56.0, 26.3, 15.4; HRMS (ESI) m/z: 291.1337 found (calcd for $C_{15}H_{19}N_2O_4^+$, $[M+H]^+$ 291.1339).

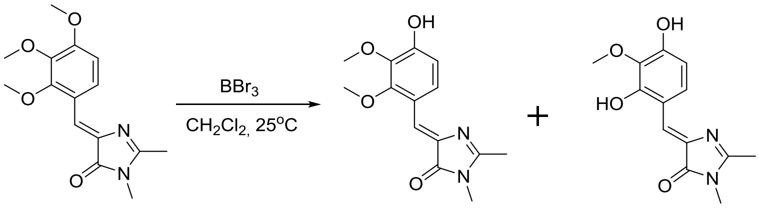

To the solution of (Z)-5-(2,3,4-trimethoxybenzylidene)-2,3-dimethyl-3,5-dihydro-4H-imidazol-4-one (5 mmol) in CH_2Cl_2 (50 mL) borone tribromide in CH_2Cl_2 (1M, 5 mL, 5 mmol) were added. The mixture was stirred for 2 h at room temperature under inert atmosphere. The mixture was diluted with $CHCl_3$ (100 mL), washed with water (2x50 mL) and brine (2x50 mL) and dried over Na_2SO_4. The solvent was evaporated and the mixture of products was separated by column chromatography ($CHCl_3$-EtOH, 49:1).

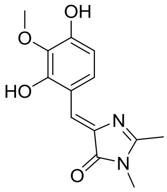

(*Z*)-5-(2,4-dihydroxy-3-methoxybenzylidene)-2,3-dimethyl-3,5-dihydro-4*H*-imidazol-4-one (N 1042)

Orange solid (0.46 g, 35%); mp =231-234 ºC; ^{1}H NMR (300 MHz, DMSO-d_6) δ ppm 12.71 (br. s., 1 H), 8.41 (br. s., 1 H), 7.37 (d, *J*=8.8 Hz, 1 H), 7.12 (s, 1 H), 6.58 (d, *J*=8.8 Hz, 1 H), 3.81 (s, 3 H), 3.12 (s, 3 H), 2.36 (s, 3 H); ^{13}C NMR (75 MHz, DMSO-d_6) δ ppm 167.9, 159.6, 151.3, 146.7, 134.6, 132.3, 125.9, 125.7, 114.4, 104.1, 55.8, 26.4, 15.1; HRMS (ESI) m/z: 263.1024 found (calcd for $C_{13}H_{15}N_2O_4^+$, $[M+H]^+$ 263.1026).

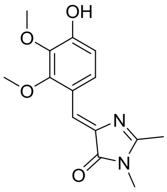

(*Z*)-5-(4-hydroxy-2,3-dimethoxybenzylidene)-2,3-dimethyl-3,5-dihydro-4*H*-imidazol-4-one (N 1027)

Yellow solid (0.41 g, 30%); mp = 193-196 ºC; ^{1}H NMR (300 MHz, DMSO-d_6) δ ppm 12.44 (br. s., 1 H), 7.75 (d, *J*=8.9 Hz, 1 H), 7.15 (s, 1 H), 6.64 (d, *J*=9.0 Hz, 1 H), 3.83 (s, 3 H), 3.67 (s, 3 H), 3.12 (s, 3 H), 2.37 (s, 3 H); ^{13}C NMR (75 MHz, DMSO-d_6) δ ppm 168.1, 160.3, 156.2, 151.9, 136.7, 133.0, 130.4, 124.5, 115.1, 104.1, 59.8, 55.9, 26.4, 15.1; HRMS (ESI) m/z: 277.1178 found (calcd for $C_{14}H_{17}N_2O_4^+$, $[M+H]^+$ 277.1183).

Back to Contents

***tert*-butyl (*Z*)-(2-(4-(4-hydroxy-3,5-dimethoxybenzylidene)-2-methyl-5-oxo-4,5-dihydro-1*H*-imidazol-1-yl)ethyl)carbamate (AR556)**

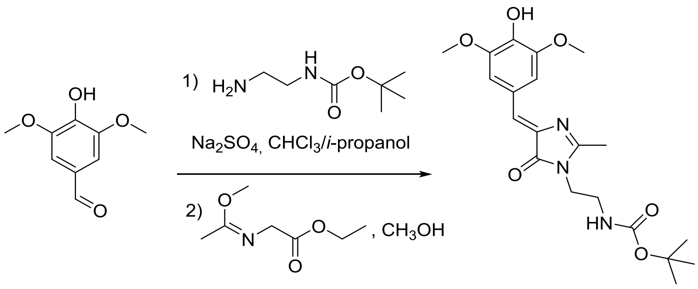

To a solution of 4-hydroxy-3,5-dimethoxybenzaldehyde (1.82 g, 10 mmol) in CH_2Cl_2/*i*-propanol (50 mL, v/v 10:1) *tert*-butyl (2-aminoethyl)carbamate (2.40 g, 15 mmol) and anhydrous Na_2SO_4 (10 g) were added. The mixture was stirred for 48 h at room temperature, filtered and dried over anhydrous Na_2SO_4. The solvent was evaporated under reduced pressure and ethyl 2-((1-methoxyethylidene)amino)acetate (3,18 g, 20 mmol) was added to the residue followed by the addition of methanol (10 mL). The mixture was stirred for 24 h at room temperature, solvents were evaporated and the product was purified by column chromatography on silica gel (0-1% MeOH in CH_2Cl_2). Yellow solid (2.93 g, 72 %); ^{1}H NMR (700 MHz, $CDCl_3$) δ ppm 7.50 (s, 2 H), 6.99 (s, 1 H), 5.92 (s, 1 H), 4.91-4.82 (m, 1H), 3.94 (s, 6 H), 3.75 (t, *J*=6.2 Hz, 2 H), 3.38-3.32 (m, 2 H), 2.38 (s, 3 H), 1.42 (s, 9 H); ^{13}C NMR (176 MHz, $CDCl_3$) δ ppm 170.9, 161.2, 156.0, 147.0, 137.5, 136.7, 128.0, 125.7, 109.4, 79.8, 56.3, 40.3, 39.5, 28.3, 15.6; HRMS (ESI) m/z: 406.1971 found (calcd for $C_{20}H_{28}N_3O_6^+$, $[M+H]^+$ 406.1973).

Preparation of (Z)-3-(4-hydroxyphenethyl)-2-methyl-5-(thiophene-2-ylmethylene)-3,5-dihydro-4H-imidazol-4-one

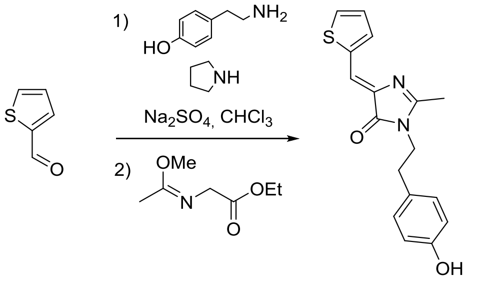

The thiophene-2-carbaldehyde (10 mmol) was dissolved in $CHCl_3$ (50 mL) and mixed with 4-(2-aminoethyl)phenol (10.5 mmol), pyrrolidine (7 mg, 0.1 mmol) and anhydrous Na_2SO_4 (10 g). The mixture was stirred for 72 h at room temperature, filtered and dried over the additional Na_2SO_4. The solvent was evaporated and ethyl((methoxy)amino)acetate (12 mmol) was added to the residue in 5 mL of methanol. The mixture was stirred for 4 days at room temperature, solvents were evaporated and the product was purified by column chromatography ($CHCl_3$-EtOH 50/1).

Back to Contents

Yellow solid (2.22 g, 71%); mp ~200 ºC with decomposition; ^{1}H NMR (700 MHz, DMSO-d_6) δ ppm 9.22 (s, 1 H), 7.84 (d, J=5.0 Hz, 1 H), 7.66 (d, J=3.4 Hz, 1 H), 7.29 (s, 1 H), 7.15 (dd, J=5.1, 3.7 Hz, 1 H), 6.97 (d, J=8.4 Hz, 2 H), 6.67 (d, J=8.4 Hz, 2 H), 3.70 (t, J=7.1 Hz, 2 H), 2.74 (t, J=7.1 Hz, 2 H), 2.05 (s, 3 H); ^{13}C NMR (75 MHz, DMSO-d_6) δ ppm 169.0, 162.3, 156.0, 137.3, 136.1, 134.7, 134.2, 129.8, 128.2, 127.6, 119.0, 115.3, 42.1, 33.5, 15.1; HRMS (ESI) m/z: 313.1003 found (calcd for $C_{17}H_{17}N_2O_2S^+$, $[M+H]^+$ 313.1005).

Back to Contents

2.2 Preparation of 5-(Z)-benzylidene-2-(E)-arylvinyl-3-methyl-3,5-dihydro-4H-imidazol-4-ones

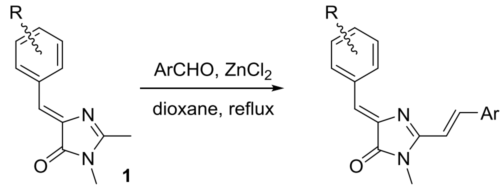

To the solution of 5-(Z)-benzylidene-2,3-dimethyl-3,5-dihydro-4H-imidazol-4-one (1 mmol) in dioxane (5 mL) anhydrous zinc chloride (30 mg, 0.22 mmol) and corresponding aldehyde (1.2 mmol) were added. The mixture was refluxed for 1-6 h and the solvent was evaporated. The mixture was dissolved in EtOAc (50 mL) and washed by EDTA solution (0.5%, 10 mL), water (3x10 mL) and brine (1x10 mL). The mixture was dried over Na_2SO_4. The solvent was evaporated and the product was purified by column chromatography ($CHCl_3$-EtOH, typically 19:1).

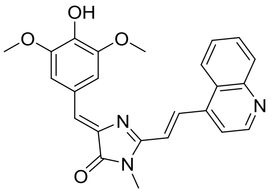

5-((*Z*)-4-hydroxy-3,5-dimethoxybenzylidene)-3-methyl-2-((*E*)-2-(quinolin-4-yl)vinyl)-3,5-dihydro-4*H*-imidazol-4-one (N 1196)

Dark red solid (95 mg, 23%); mp = 273-276ºC; ^{1}H NMR (700 MHz, DMSO-d_6) δ ppm 9.30 (br. s., 1 H), 9.01 (d, *J*=4.6 Hz, 1 H), 8.77 (d, *J*=15.6 Hz, 1 H), 8.28 (d, *J*=8.4 Hz, 1 H), 8.09 - 8.13 (m, 2 H), 7.81 - 7.89 (m, 3 H), 7.74 (t, *J*=7.4 Hz, 1 H), 7.60 (d, *J*=15.6 Hz, 1 H), 7.10 (s, 1 H), 3.92 (s, 6 H), 3.34 (s, 3 H); ^{13}C NMR (201 MHz, DMSO-d_6) δ ppm 169.7, 158.1, 150.2, 148.3, 147.8, 139.3, 139.0, 137.0, 132.4, 129.8, 129.6, 127.6, 127.2, 125.4, 124.9, 122.7, 120.2, 118.0, 110.4, 55.7, 26.3; HRMS (ESI) m/z: 414.1463 found (calcd for $C_{24}H_{20}N_3O_4^-$, [M-H]$^-$ 414.1459).

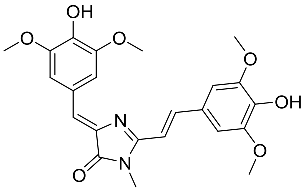

5-((*Z*)-4-hydroxy-3,5-dimethoxybenzylidene)-2-((*E*)-4-hydroxy-3,5-dimethoxystyryl)-3-methyl-3,5-dihydro-4*H*-imidazol-4-one (N 1193)

Red solid (62 mg, 39%); mp = 235-238 ºC; ^{1}H NMR (700 MHz, DMSO-d_6) δ ppm 9.13 (s, 1 H), 9.01 (s, 1 H), 7.89 (d, *J*=15.6 Hz, 1 H), 7.74 (s, 2 H), 7.10 (s, 2 H), 7.06 (d, *J*=15.6 Hz, 1 H), 6.92 (s, 1 H), 3.87 (s, 6 H), 3.85 (s, 6 H), 3.29 (s, 3 H); ^{13}C NMR (176 MHz, DMSO-d_6) δ ppm

Back to Contents

170.0, 159.1, 148.1, 147.9, 140.6, 138.5, 138.5, 137.4, 125.7, 125.2, 125.1, 110.9, 110.1, 106.5, 56.2, 56.0, 26.4; HRMS (ESI) m/z: 439.1516 found (calcd for $C_{23}H_{23}N_2O_7^-$, $[M-H]^-$ 439.1511).

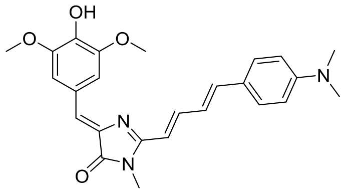

2-((1*E*,3*E*)-4-(4-(dimethylamino)phenyl)buta-1,3-dien-1-yl)-5-((*Z*)-4-hydroxy-3,5-dimethoxybenzylidene)-3-methyl-3,5-dihydro-4*H*-imidazol-4-one (N 1195)

Dark red solid (52 mg, 12%); mp ~200ºC with decomposition; ^{1}H NMR (700 MHz, DMSO-d_6) δ ppm 9.12 (br. s., 1 H), 7.69 - 7.74 (m, 3 H), 7.45 (d, *J*=8.8 Hz, 2 H), 6.97 - 7.06 (m, 2 H), 6.89 (s, 1 H), 6.74 (d, *J*=9.0 Hz, 2 H), 6.55 (d, *J*=14.7 Hz, 1 H), 3.86 (s, 6 H), 3.19 (s, 3 H), 2.98 (s, 6 H); ^{13}C NMR (201 MHz, DMSO-d_6) δ ppm 169.7, 158.8, 150.8, 147.8, 141.3, 140.5, 138.4, 137.6, 128.6, 125.2, 124.6, 123.8, 123.0, 113.7, 112.0, 110.0, 56.0, 40.0, 26.1; HRMS (ESI) m/z: 434.2066 found (calcd for $C_{25}H_{28}N_3O_4^+$, $[M+H]^+$ 434.2074).

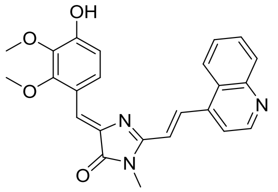

5-((*Z*)-4-hydroxy-2,3-dimethoxybenzylidene)-3-methyl-2-((*E*)-2-(quinolin-4-yl)vinyl)-3,5-dihydro-4*H*-imidazol-4-one (N 1197)

Red solid (104 mg, 69%); mp = 216-219 ºC; ^{1}H NMR (700 MHz, DMSO-d_6) δ ppm 11.75 (br. s., 1 H), 9.01 (d, *J*=4.6 Hz, 1 H), 8.59 (d, *J*=15.8 Hz, 1 H), 8.36 (d, *J*=8.4 Hz, 1 H), 8.16 (d, *J*=8.8 Hz, 1 H), 8.12 (d, *J*=8.4 Hz, 1 H), 8.08 (d, *J*=4.4 Hz, 1 H), 7.86 (t, *J*=7.5 Hz, 1 H), 7.6 (t, *J*=7.6 Hz, 1 H), 7.56 (d, *J*=15.6 Hz, 1 H), 7.39 (s, 1 H), 6.74 (d, *J*=9.0 Hz, 1 H), 3.88 (s, 3 H), 3.74 (s, 3 H), 3.36 (s, 3 H); ^{13}C NMR (176 MHz, DMSO-d_6) δ ppm 168.5, 156.3, 156.2, 152.0, 150.3, 148.3, 139.5, 136.5, 134.4, 133.0, 130.2, 129.8, 129.7, 127.4, 125.3, 123.2, 120.2, 118.4, 115.7, 104.6, 59.9, 55.9, 26.7; HRMS (ESI) m/z: 416.1601 found (calcd for $C_{24}H_{22}N_3O_4^+$, $[M+H]^+$ 416.1605).

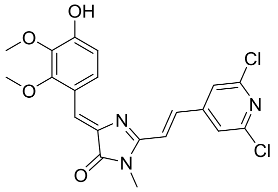

2-((*E*)-2-(2,6-dichloropyridin-4-yl)vinyl)-5-((*Z*)-4-hydroxy-2,3-dimethoxybenzylidene)-3-methyl-3,5-dihydro-4*H*-imidazol-4-one (N 1198)

Red solid (94 mg, 60%); mp ~260 ºC with decomposition; ^{1}H NMR (700 MHz, DMSO-d_6) δ ppm 8.18 (d, *J*=8.8 Hz, 1 H), 8.10 (s, 2 H), 7.80 (d, *J*=16.0 Hz, 1 H), 7.67 (d, *J*=16.0 Hz, 1 H),

Back to Contents

7.39 (s, 1 H), 6.68 (d, *J*=9.0 Hz, 1 H), 3.87 (s, 3 H), 3.72 (s, 3 H), 3.31 (s, 3 H); ^{13}C NMR (176 MHz, DMSO-d_6) δ ppm 168.6, 156.3, 155.9, 152.0, 149.9, 148.8, 136.3, 134.7, 133.8, 130.0, 121.7, 121.3, 115.7, 104.5, 60.0, 55.9, 26.7; HRMS (ESI) m/z: 432.0530 found (calcd for $C_{20}H_{16}Cl_2N_3O_4^-$, [M-H]$^-$ 432.0523).

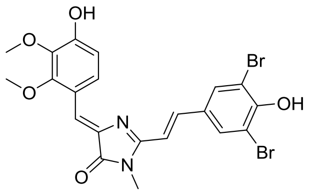

2-((*E*)-3,5-dibromo-4-hydroxystyryl)-5-((*Z*)-4-hydroxy-2,3-dimethoxybenzylidene)-3-methyl-3,5-dihydro-4*H*-imidazol-4-one (N 1199)

Red solid (101 mg, 52%); mp ~250 ºC with decomposition; ^{1}H NMR (700 MHz, DMSO-d_6) δ ppm 8.14 (s, 2 H), 8.00 (d, *J*=9.3 Hz, 1 H), 7.73 (d, *J*=15.4 Hz, 1 H), 7.21 - 7.26 (m, 2 H), 6.67 (d, *J*=9.2 Hz, 1 H), 3.86 (s, 3 H), 3.72 (s, 3 H), 3.30 (s, 3 H); ^{13}C NMR (176 MHz, DMSO-d_6) δ ppm 168.5, 156.5, 156.1, 152.2, 151.8, 137.2, 137.2, 136.5, 134.3, 132.2, 130.2, 129.7, 115.8, 113.3, 112.2, 104.4, 59.9, 55.8, 26.6; HRMS (ESI) m/z: 534.9524 found (calcd for $C_{21}H_{17}Br_2N_2O_5^-$, [M-H]$^-$ 534.9510).

***tert*-butyl (2-(2-((*E*)-3,5-dibromo-4-hydroxystyryl)-4-((*Z*)-4-hydroxy-3,5-dimethoxybenzylidene)-5-oxo-4,5-dihydro-1*H*-imidazol-1-yl)ethyl)carbamate (AR558)**

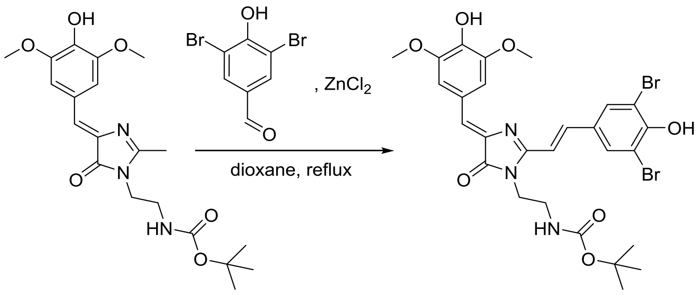

To the solution of AR556 (405 mg, 1 mmol) in dioxane (5 mL) anhydrous zinc chloride (30 mg, 0.22 mmol) and 3,5-dibromo-4-hydroxybenzaldehyde (340 mg, 1.2 mmol) were added. The mixture was refluxed for 4 h and the solvent was evaporated. The mixture was dissolved in EtOAc (50 mL), washed by EDTA solution (0.5%, 10 mL), water (3x10 mL) and brine (1x10 mL), followed by drying over Na_2SO_4. The solvent was evaporated and the product was purified by column chromatography on silica gel (0-2% EtOH in CH_2Cl_2). Orange solid (148 mg, 22 %); ^{1}H NMR (700 MHz, DMSO-d_6) δ ppm 10.42 (br s, 1 H), 9.16 (s, 1 H), 8.05 (s, 2 H), 7.79 (d, *J*=15.7 Hz, 1 H), 7.71 (s, 2 H), 7.14 (d, *J*=15.7 Hz, 1 H), 6.97 (t, *J*=5.7 Hz, 1 H), 6.94 (s, 1 H), 3.86 (s, 6 H), 3.78 (t, *J*=5.5 Hz, 2 H), 3.16-3.10 (m, 2 H), 1.29 (s, 9 H); ^{13}C NMR (176 MHz, $CDCl_3$) δ ppm 169.6, 158.0, 155.6, 152.2, 147.7, 138.4, 137.0, 136.2, 131.8, 129.6, 125.8, 124.9,

Back to Contents

113.4, 112.2, 109.9, 77.7, 55.9, 2×C overlap with DMSO, 28.0; HRMS (ESI) m/z: 666.0442 found (calcd for $C_{27}H_{30}Br_2N_3O_7^+$, $[M+H]^+$ 666.0445).

Preparation of (*Z*)-3-(4-hydroxyphenethyl)-2-((*E*)-4-hydroxystyryl)-5-(thiophen-2-ylmethylene)-3,5-dihydro-4H-imidazol-4-one (MID 161)

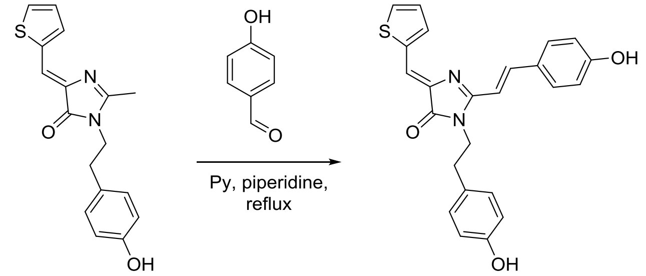

To the solution of (*Z*)-3-(4-hydroxyphenethyl)-2-methyl-5-(thiophene-2-ylmethylene)-3,5-dihydro-4*H*-imidazol-4-one (1 mmol) in pyridine (5 mL) piperidine (0.02 mL) and corresponding aldehyde (5 mmol) were added. The mixture was refluxed for 15 h and the solvent was evaporated. The mixture was dissolved in EtOAc (35 mL) and washed with phosphate buffer (pH = 7, 3x15 mL), brine (3x25 mL) and dried over Na_2SO_4. The solvent was evaporated and product was purified by column chromatography (Hexane-EtOAc 3/1).

Orange solid (200 mg, 48%); mp ~200 ºC with decomposition; ^{1}H NMR (700 MHz, DMSO-d_6) δ ppm 10.04 (br. s., 1 H), 9.13 (s, 1 H), 7.87 (d, *J*=5.0 Hz, 1 H), 7.81 (d, *J*=15.6 Hz, 1 H), 7.69 (d, *J*=2.9 Hz, 1 H), 7.59 (d, *J*=8.2 Hz, 2 H), 7.28 (s, 1 H), 7.16 - 7.18 (m, 1 H), 7.01 (d, *J*=7.8 Hz, 2 H), 6.85 (d, *J*=7.6 Hz, 2 H), 6.69 (d, *J*=15.4 Hz, 1 H), 6.64 (d, *J*=8.2 Hz, 2 H), 3.93 (t, *J*=6.9 Hz, 2 H), 2.76 (t, *J*=6.8 Hz, 2 H); ^{13}C NMR (75 MHz, DMSO-d_6) δ ppm 169.1, 159.7, 159.2, 155.9, 140.3, 138.2, 137.2, 134.3, 134.2, 130.4, 129.9, 128.1, 127.7, 126.3, 118.0, 115.9, 115.2, 109.9, 41.2, 34.2; HRMS (ESI) m/z: 417.1263 found (calcd for $C_{24}H_{21}N_2O_3S^+$, $[M+H]^+$ 417.1267).

2-(2-((*E*)-3,5-dibromo-4-hydroxystyryl)-4-((*Z*)-4-hydroxy-3,5-dimethoxybenzylidene)-5-oxo-4,5-dihydro-1*H*-imidazol-1-yl)ethan-1-aminium chloride (AR559)

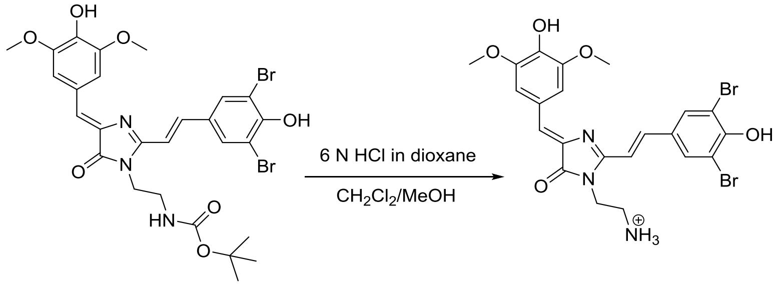

To a solution of AR558 (120 mg, 0.18 mmol) in CH_2Cl_2/methanol (10 mL, v/v 10:1) 6N HCl in dioxane (0.5 mL) was added and the mixture was stirred for 48 h at room temperature. The

Back to Contents

precipitate formed was filtered, washed with cold CH_2Cl_2 (2 x 5 mL) and dried in high vacuum. Red solid (85 mg, 83%); ^{1}H NMR (700 MHz, DMSO-d_6) δ ppm 10.47 (br s, 1 H), 9.22 (s, 1 H), 8.12 (s, 2 H), 8.09-7.99 (m, 3 H), 7.83 (d, J=15.4 Hz, 1 H), 7.74 (s, 2 H), 7.21 (d, J=15.4 Hz, 1 H), 7.00 (s, 1 H), 4.05 (t, J=5.4 Hz, 2 H), 3.87 (s, 6 H), 3.12-3.03 (m, 2 H); ^{13}C NMR (176 MHz, $CDCl_3$) δ ppm 170.1, 157.5, 152.0, 147.8, 138.7, 137.0, 136.7, 132.2, 129.9, 126.4, 124.9, 113.4, 112.1, 110.0, 55.9, 40.1, 38.0; HRMS (ESI) m/z: 565.9918 found (calcd for $C_{22}H_{22}Br_2N_3O_5^+$, $[M-Cl]^+$ 565.9921).

Back to Contents

3. Copies of ^{1}H and ^{13}C NMR spectra

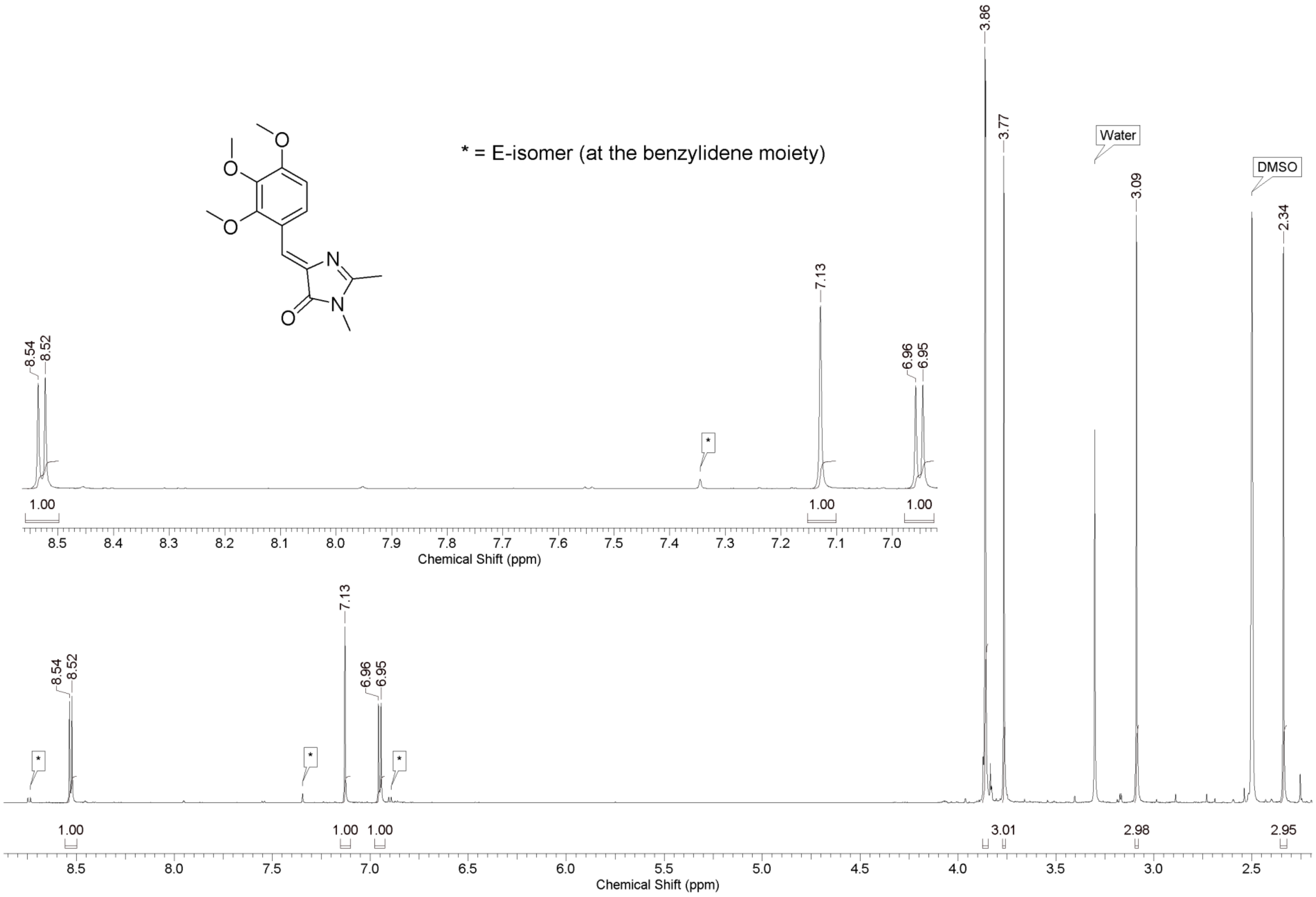

[Back to Contents]

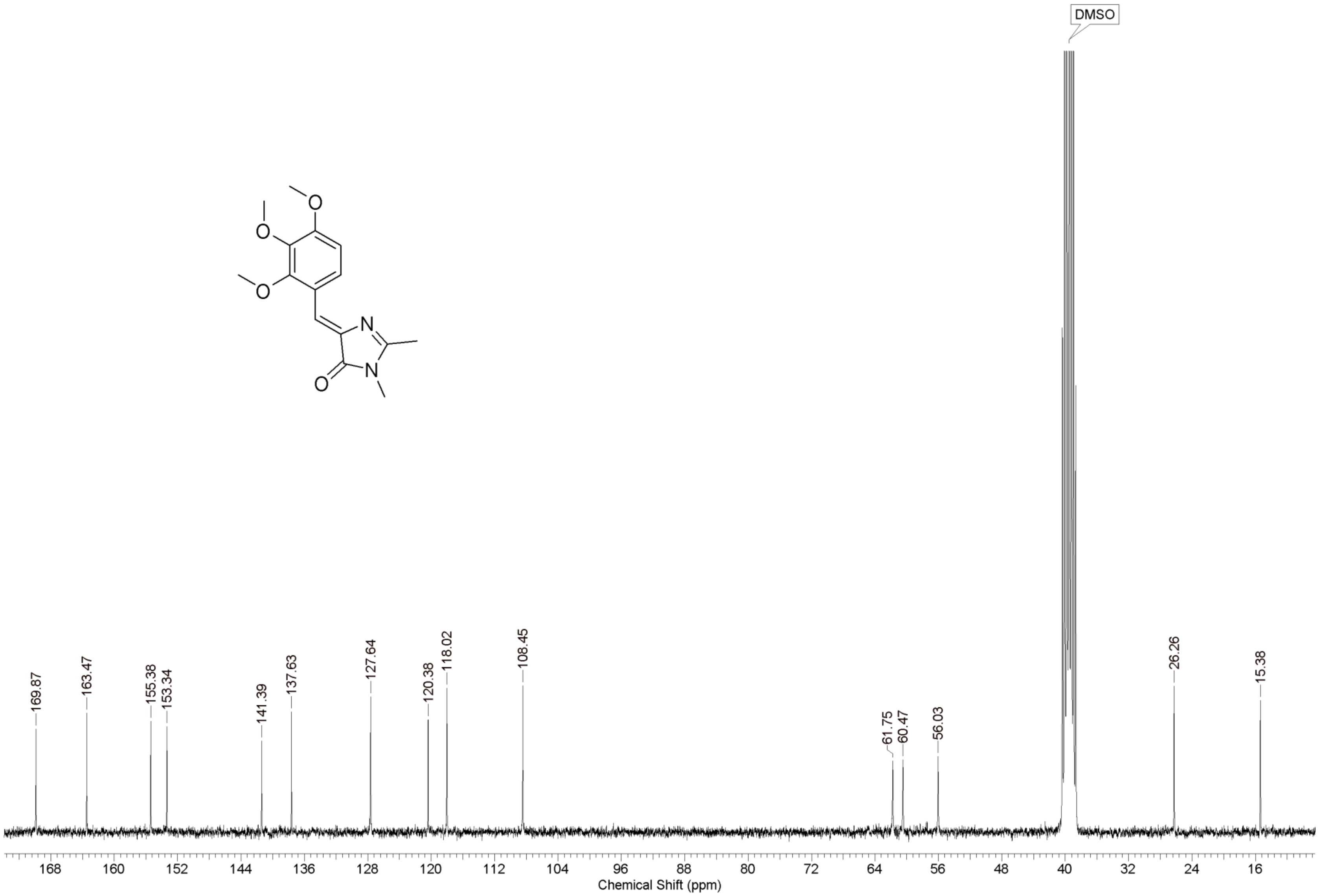

Back to Contents

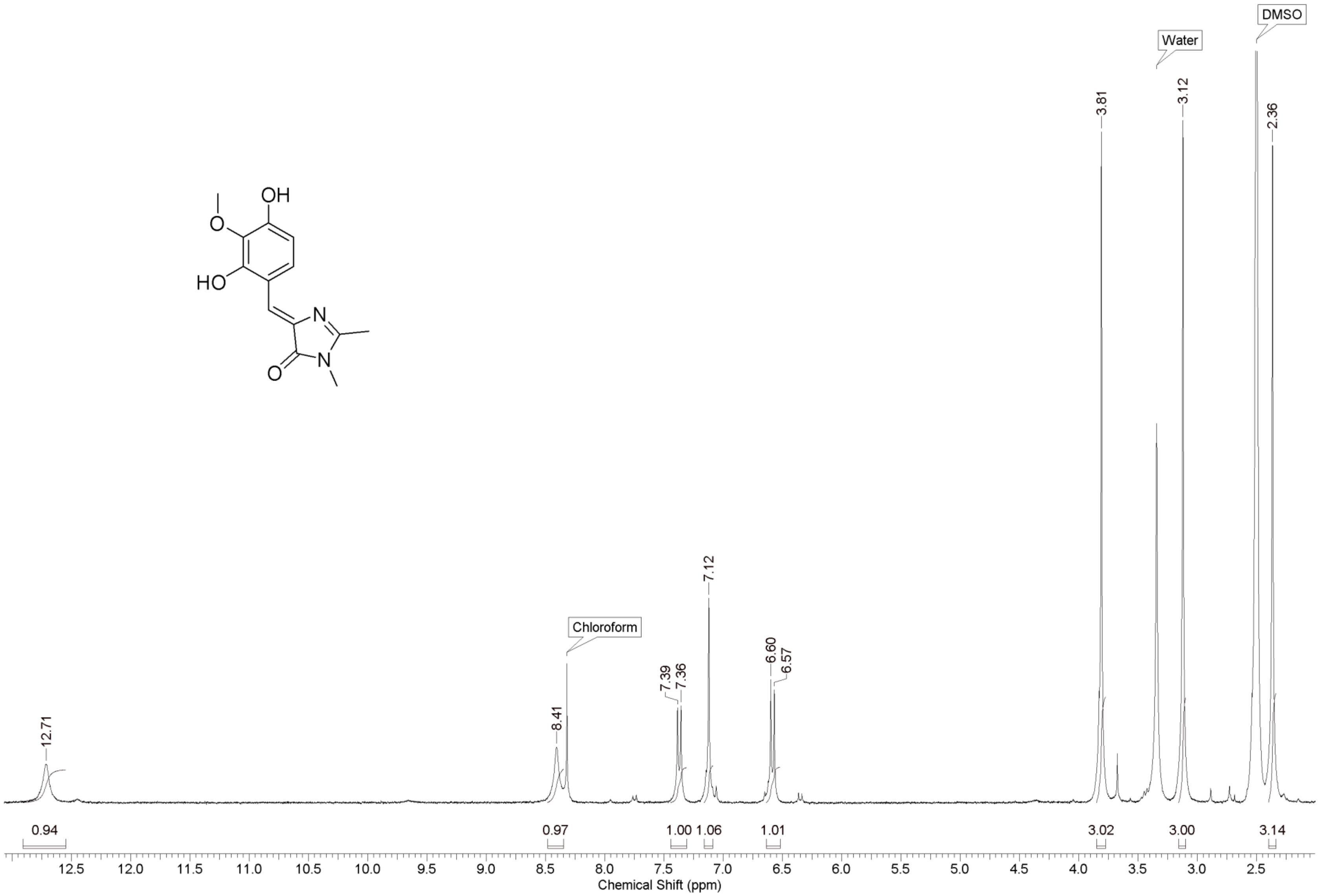

[Back to Contents]

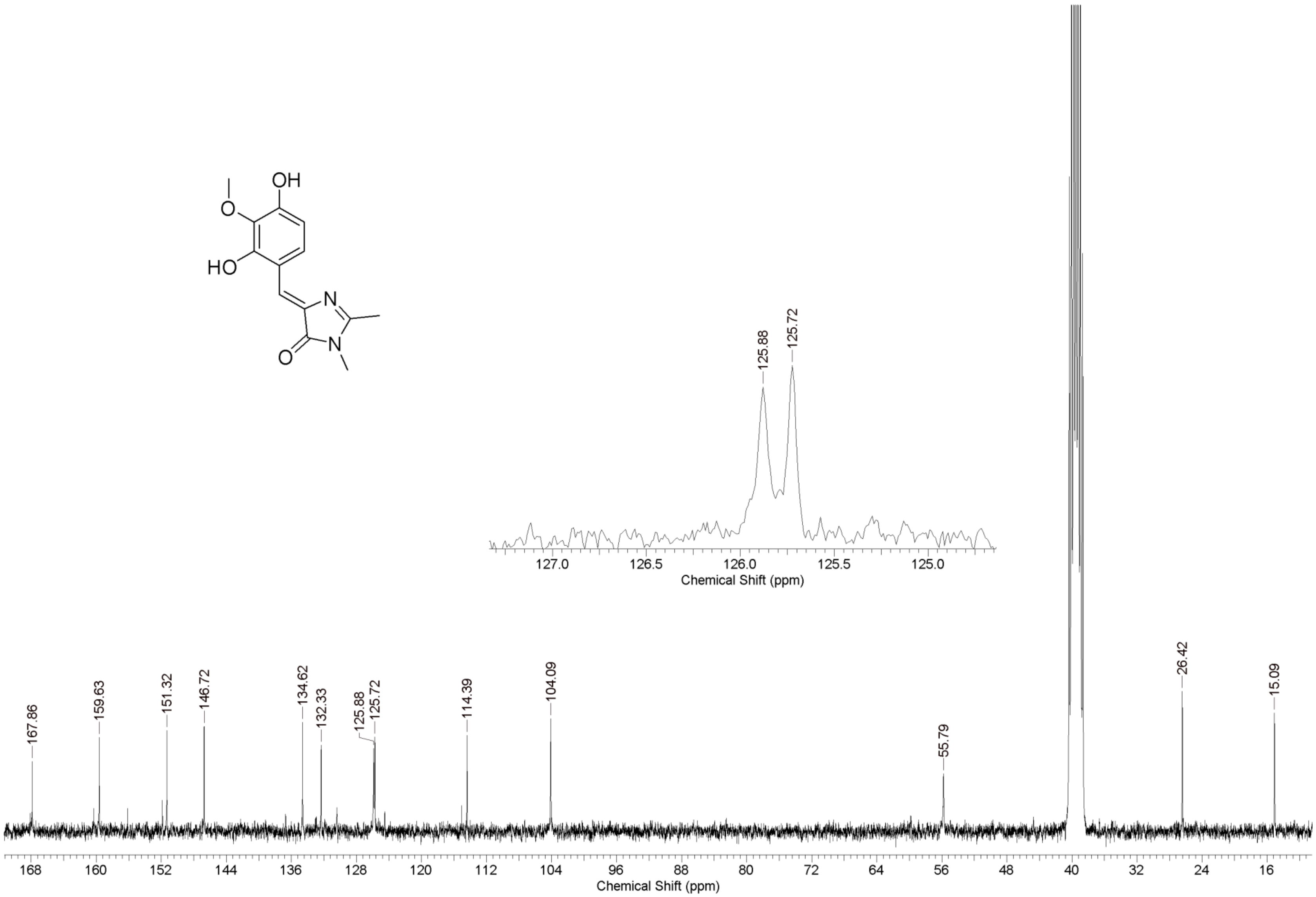

Back to Contents

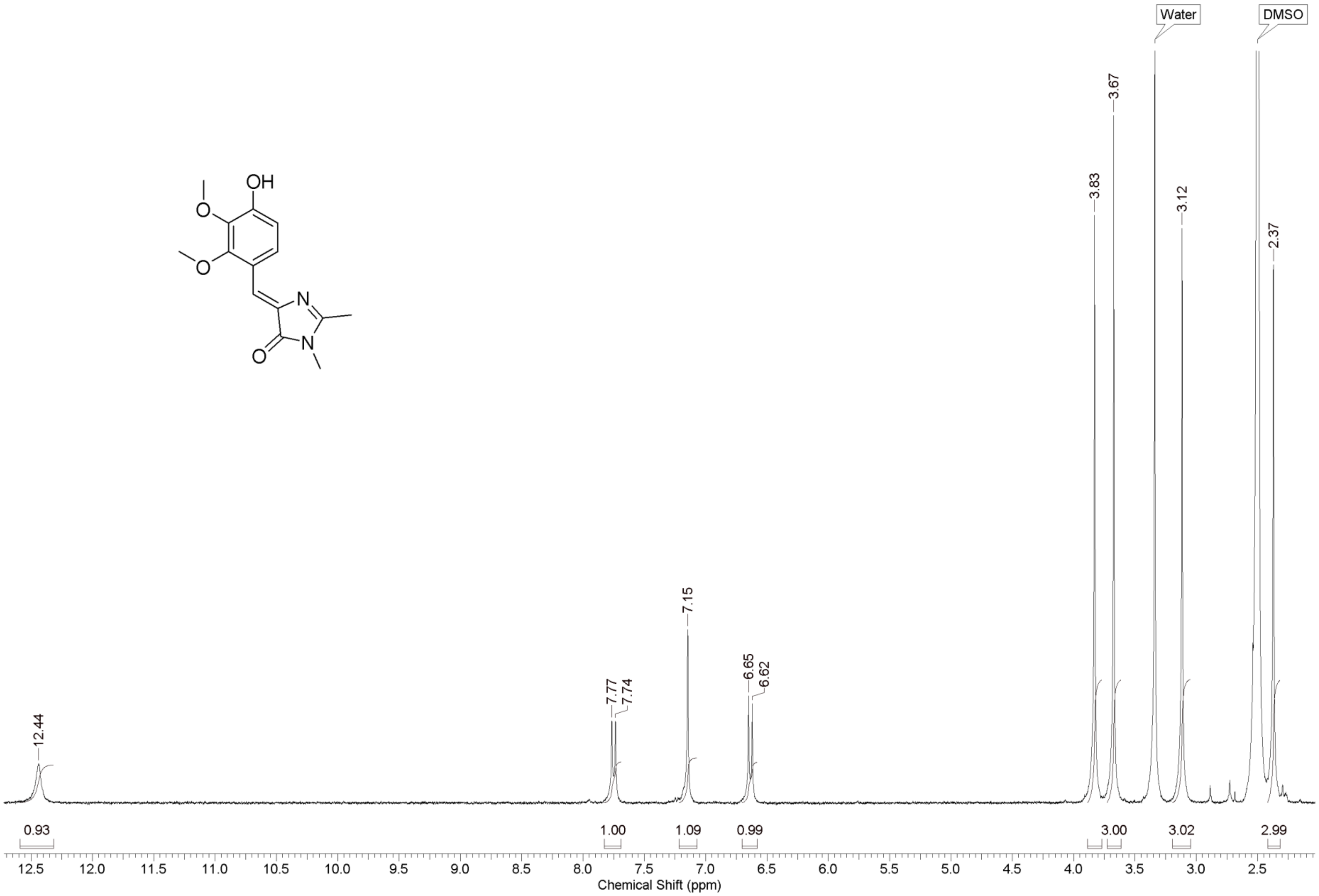

Back to Contents

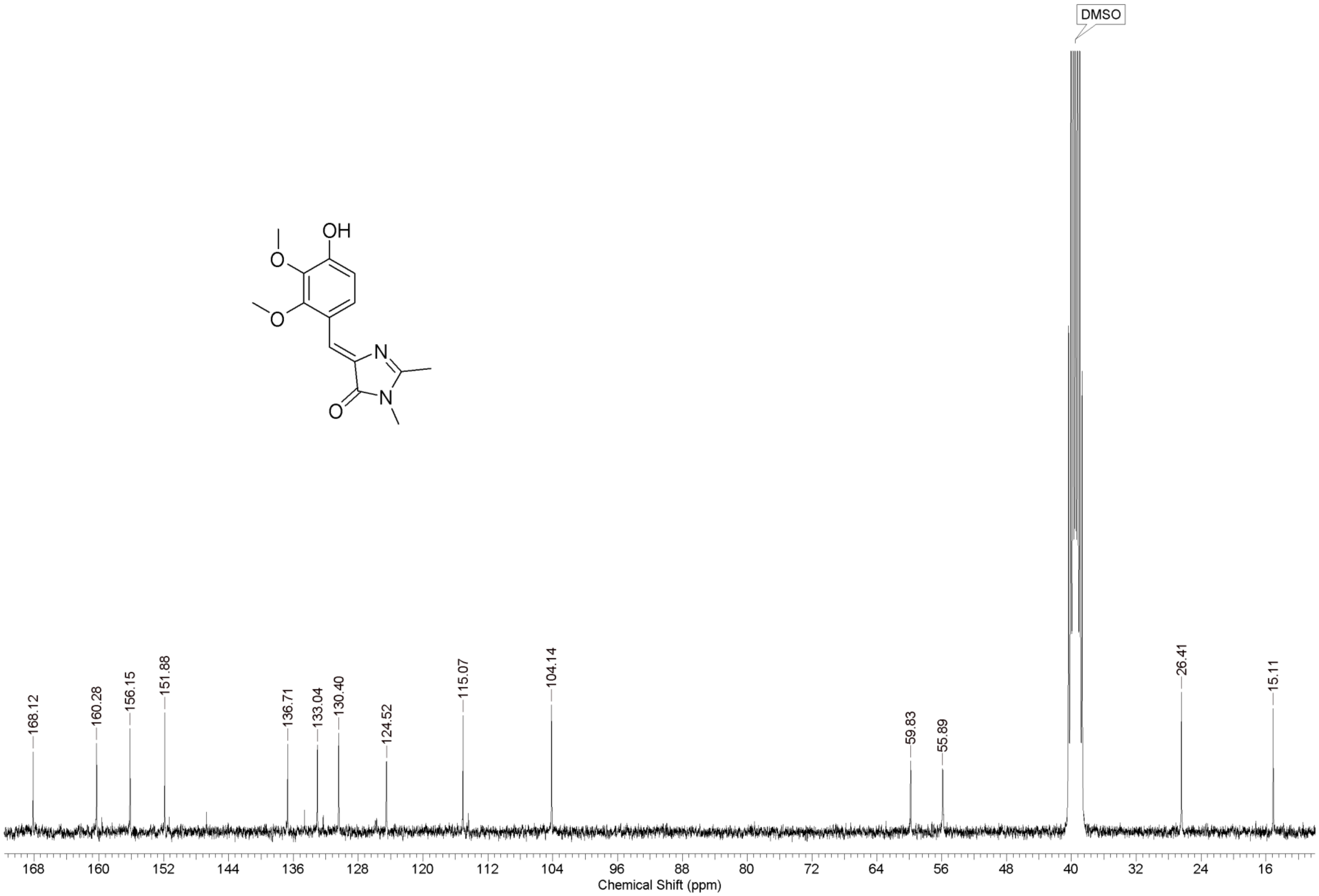

[Back to Contents]

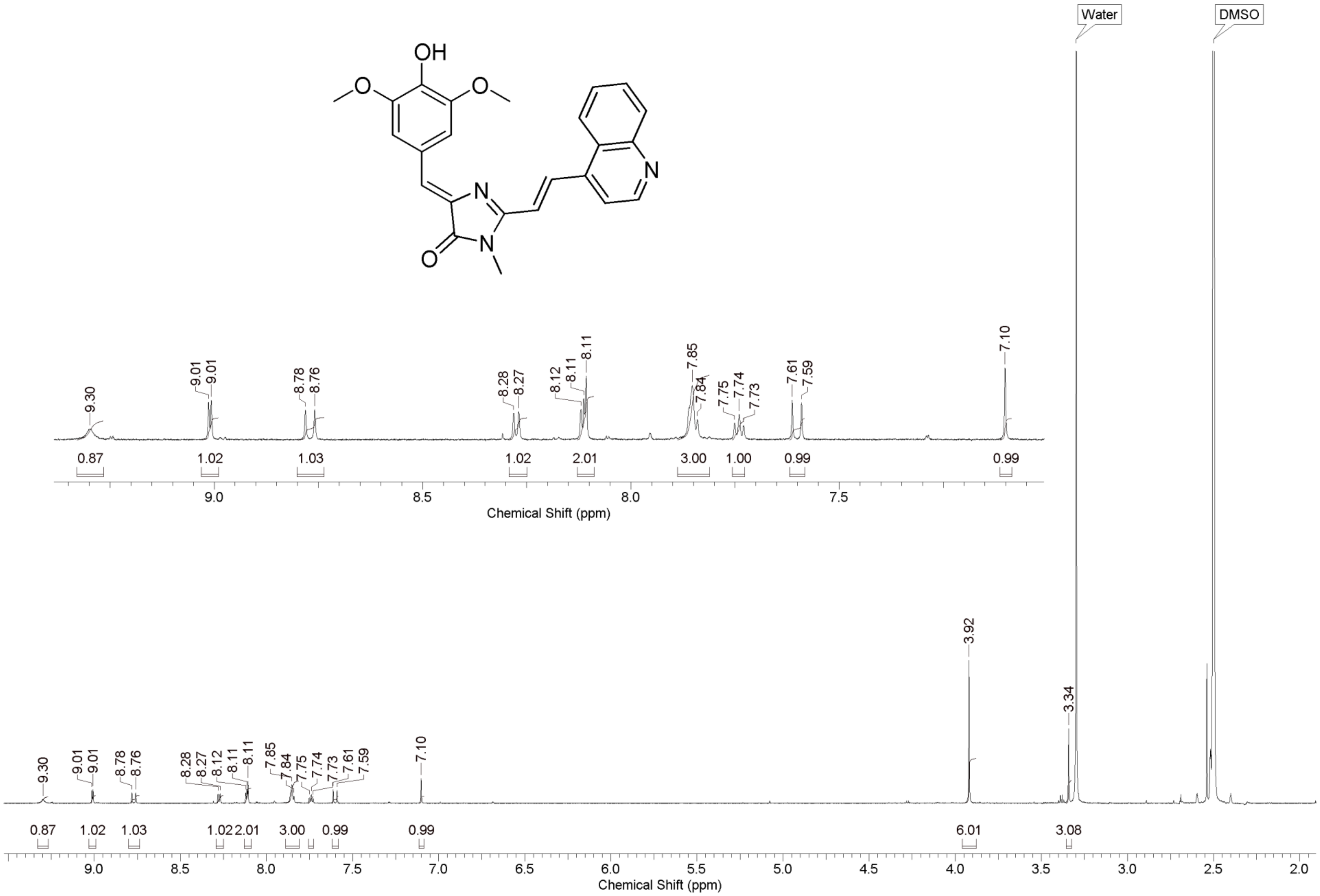

Back to Contents

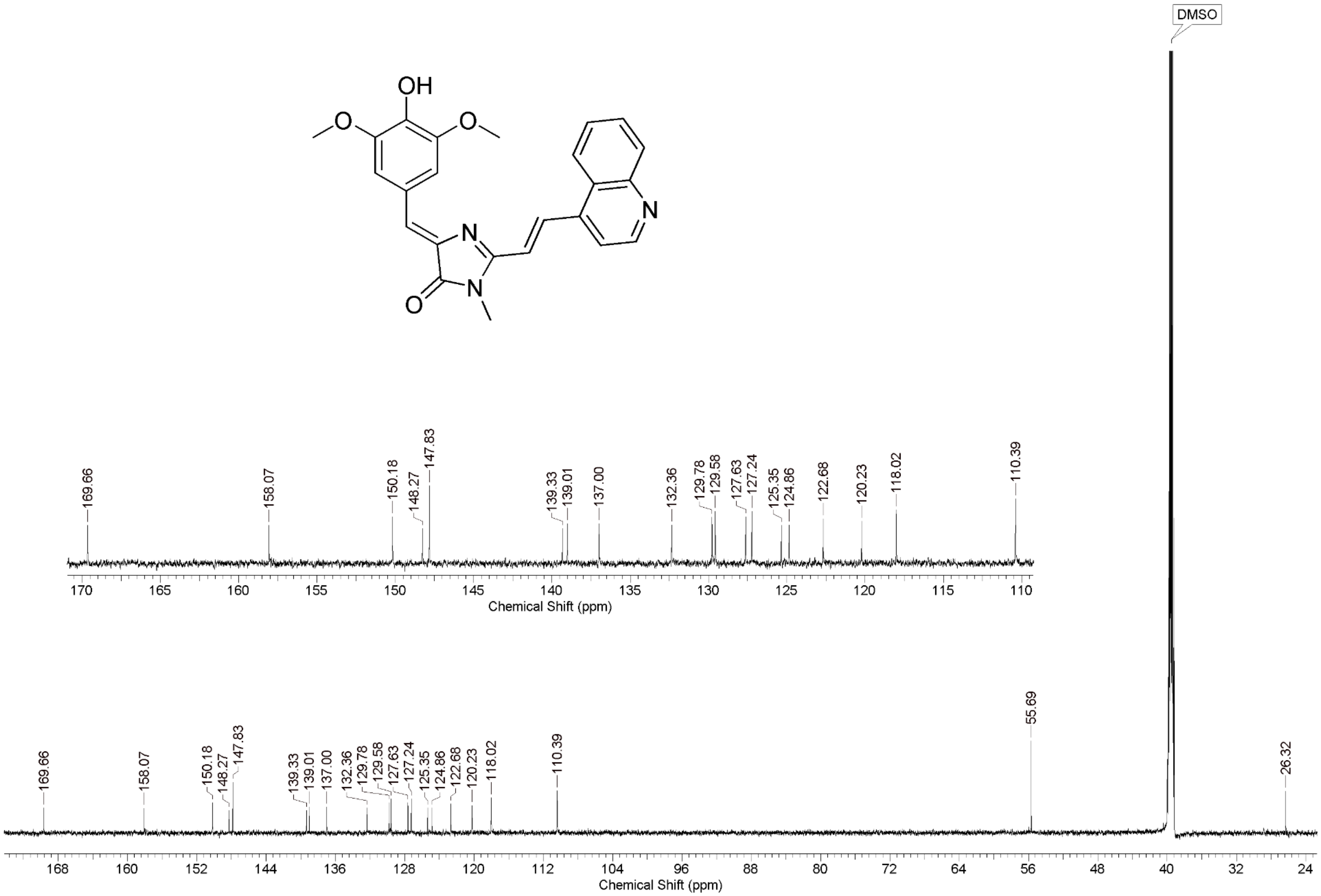

Back to Contents

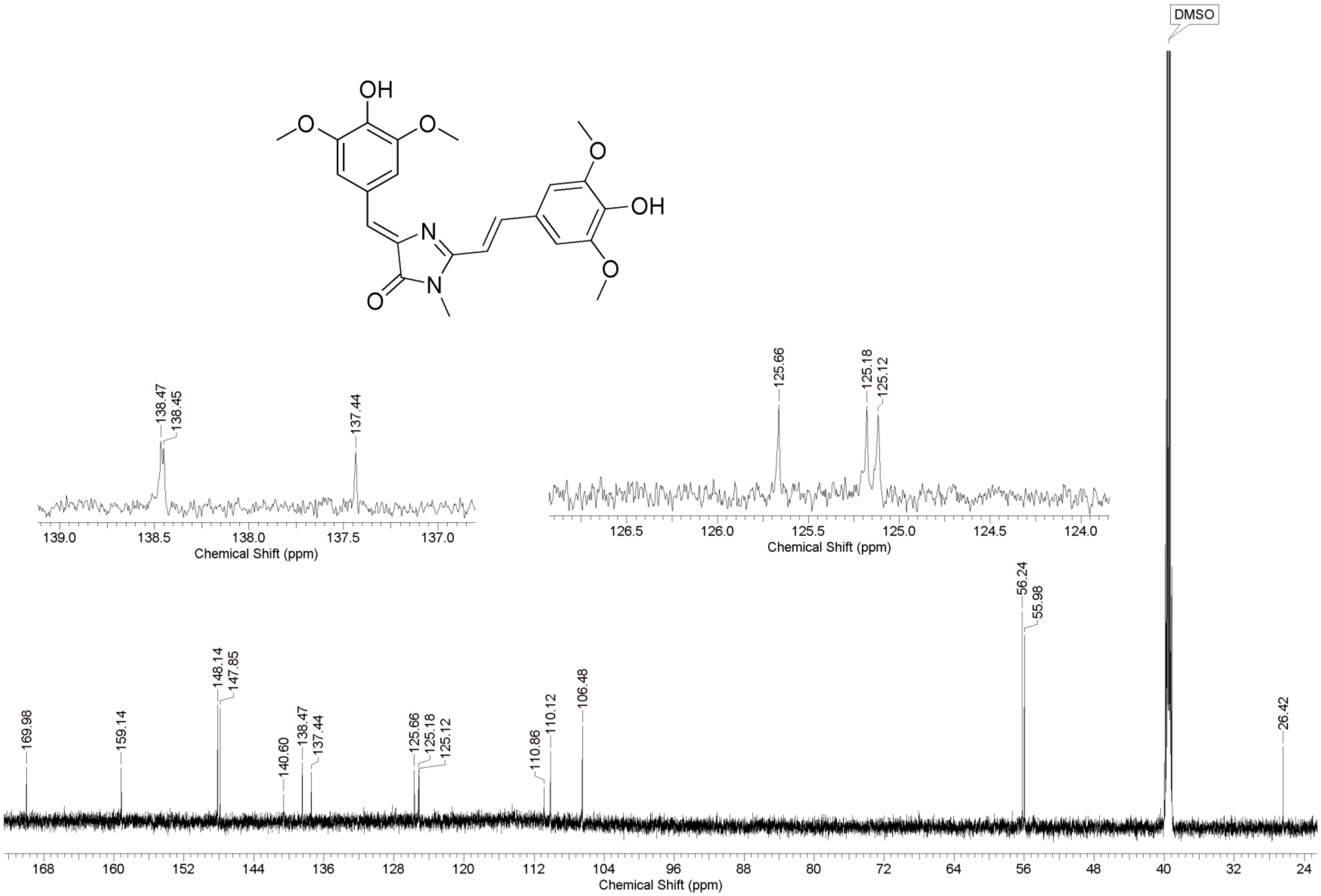

[Back to Contents]

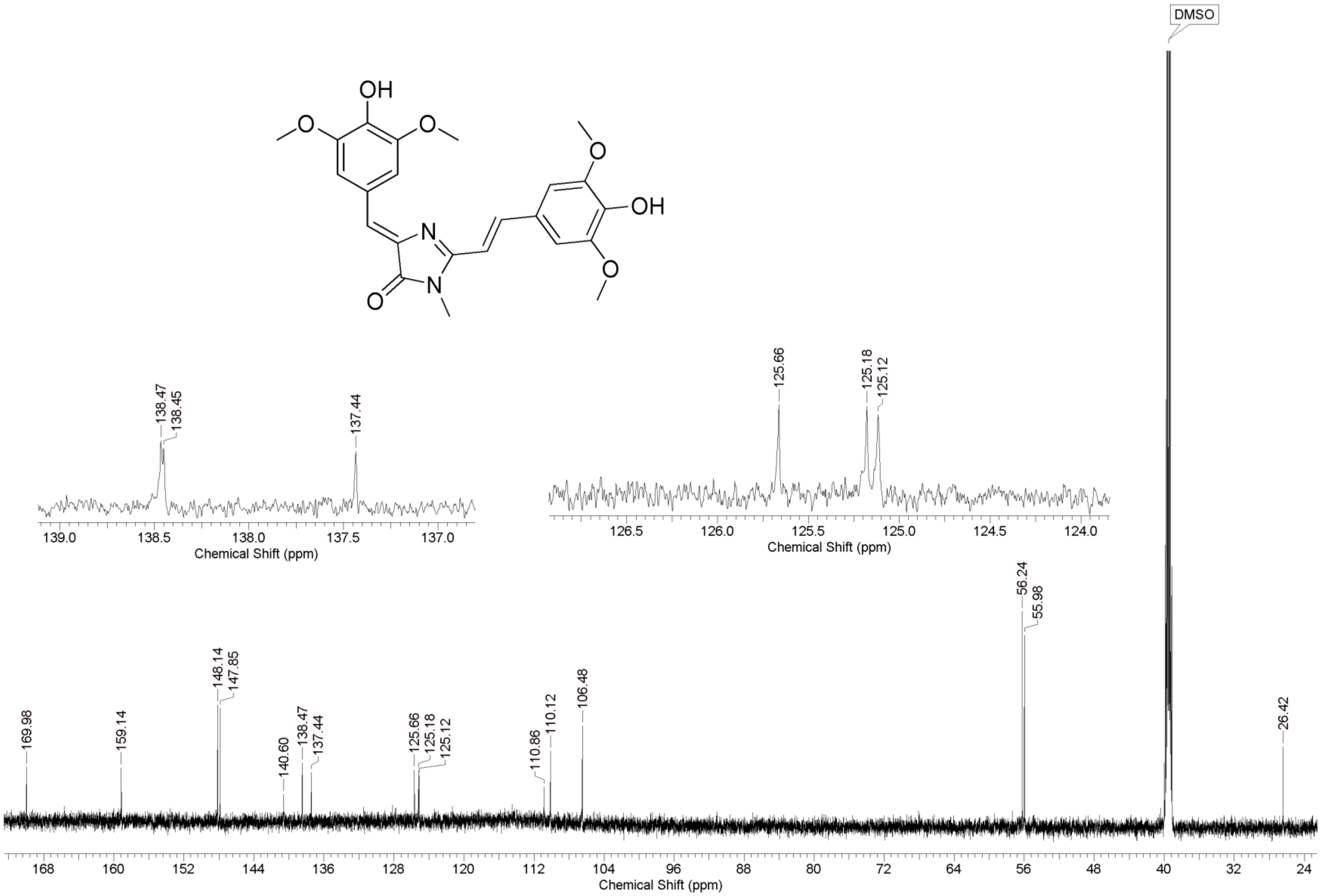

Back to Contents

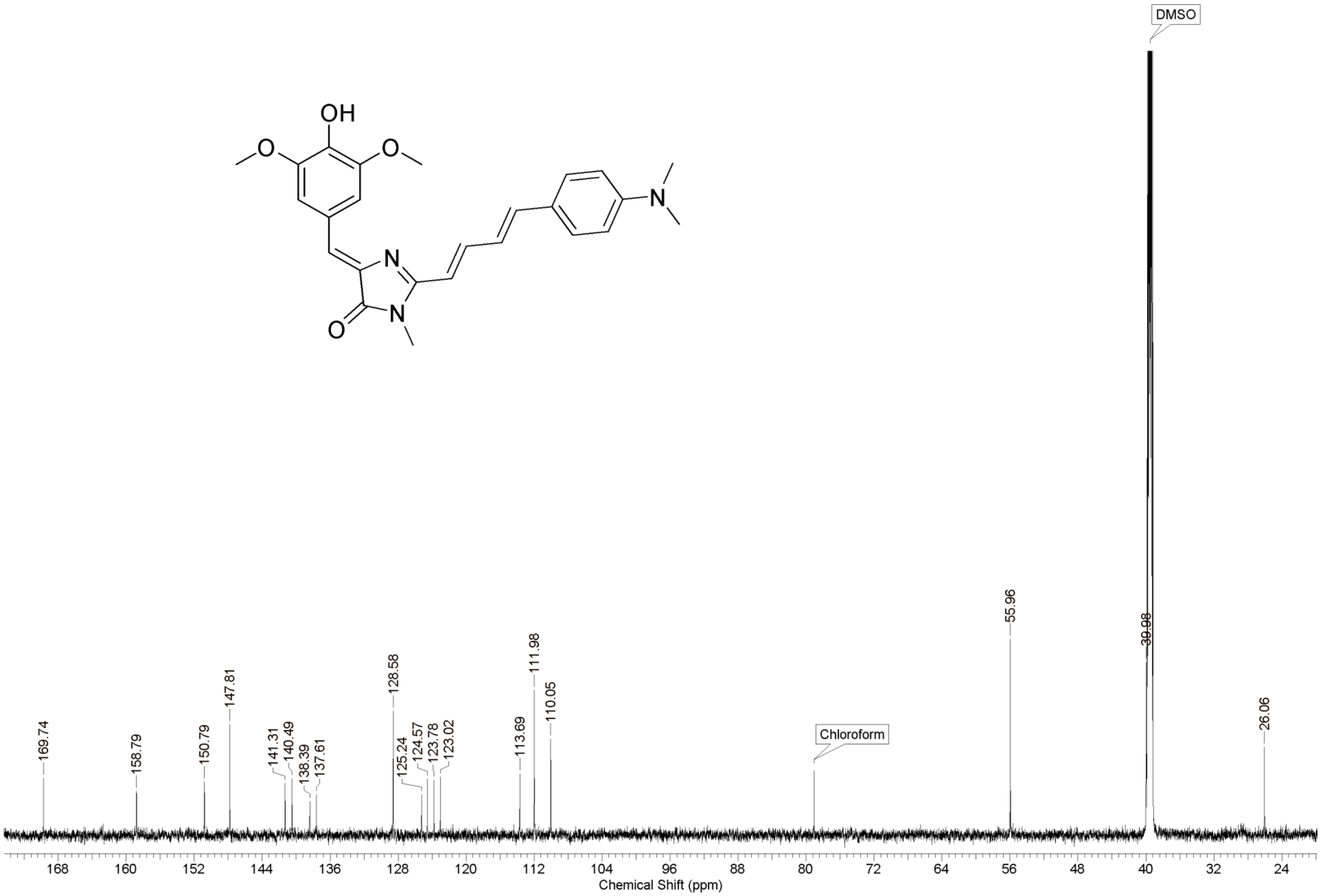

Back to Contents

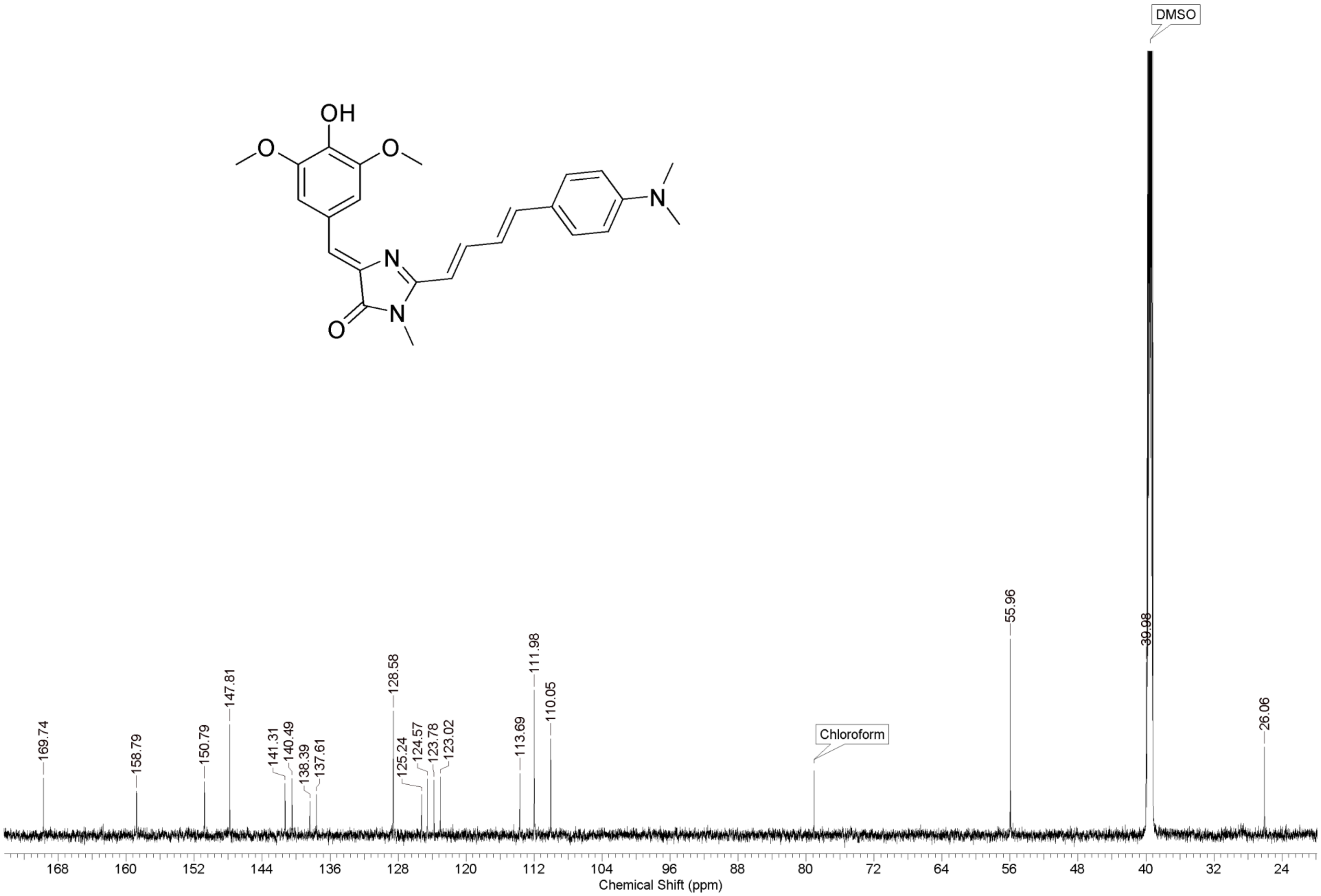

Back to Contents

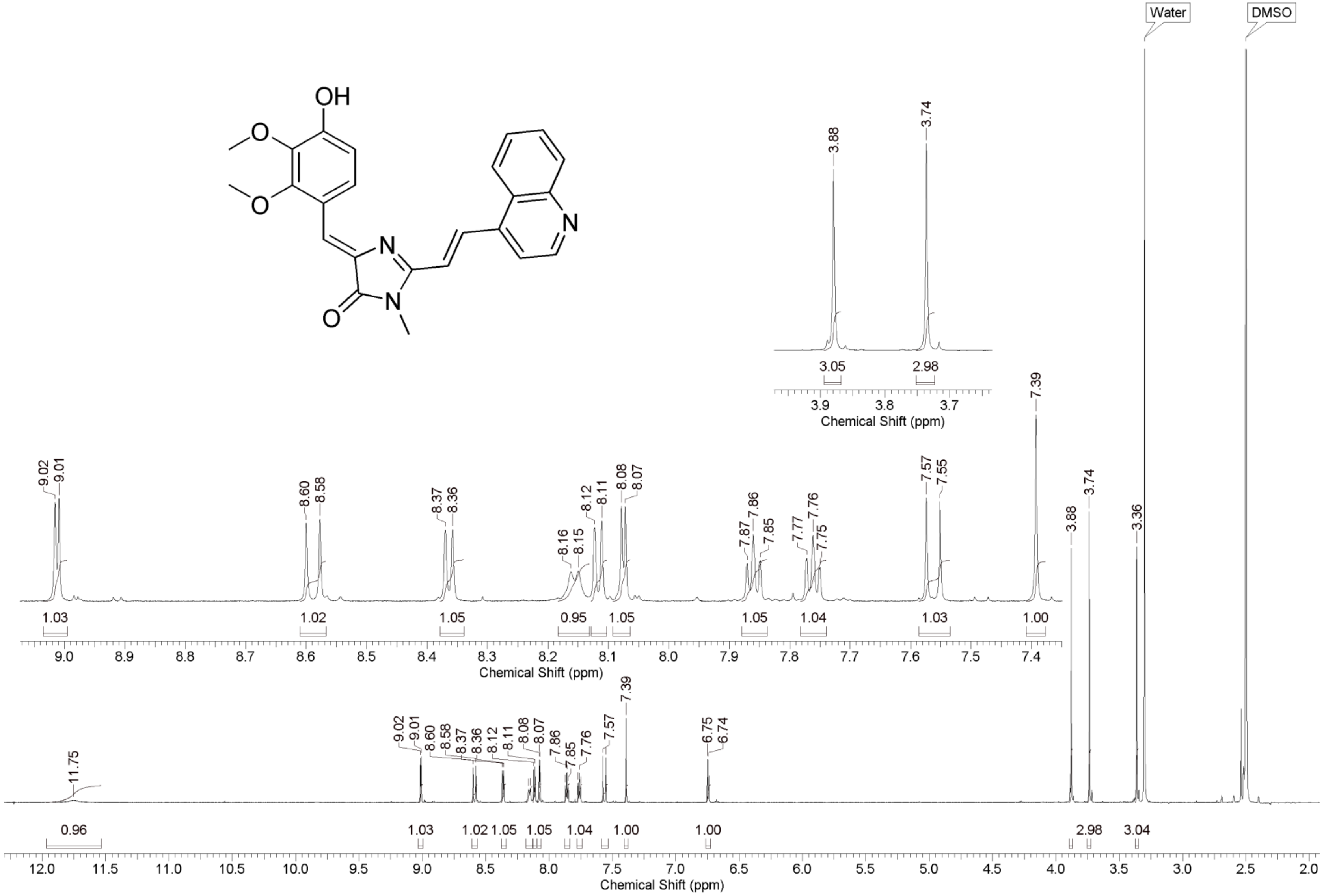

[Back to Contents]

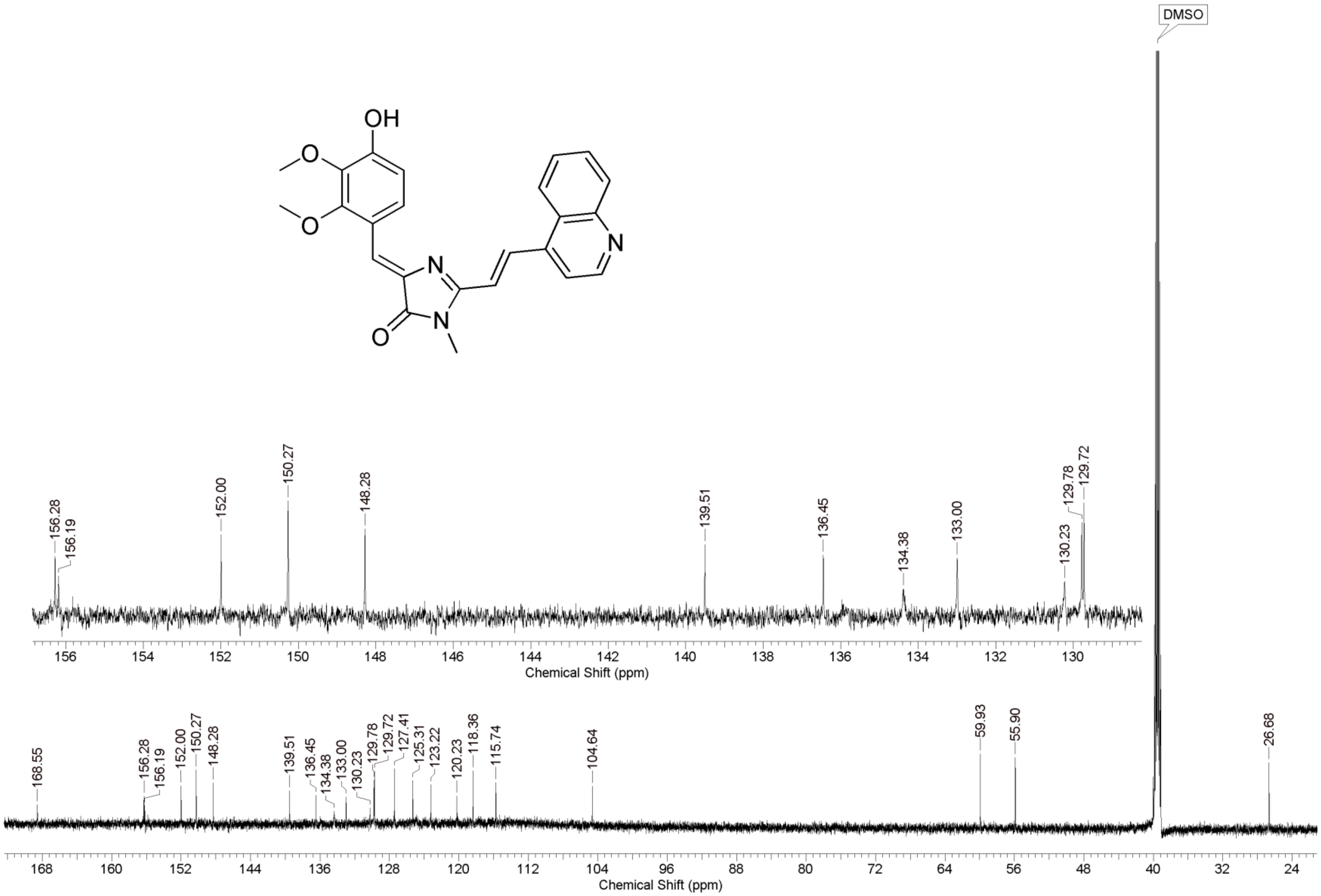

[Back to Contents](#)

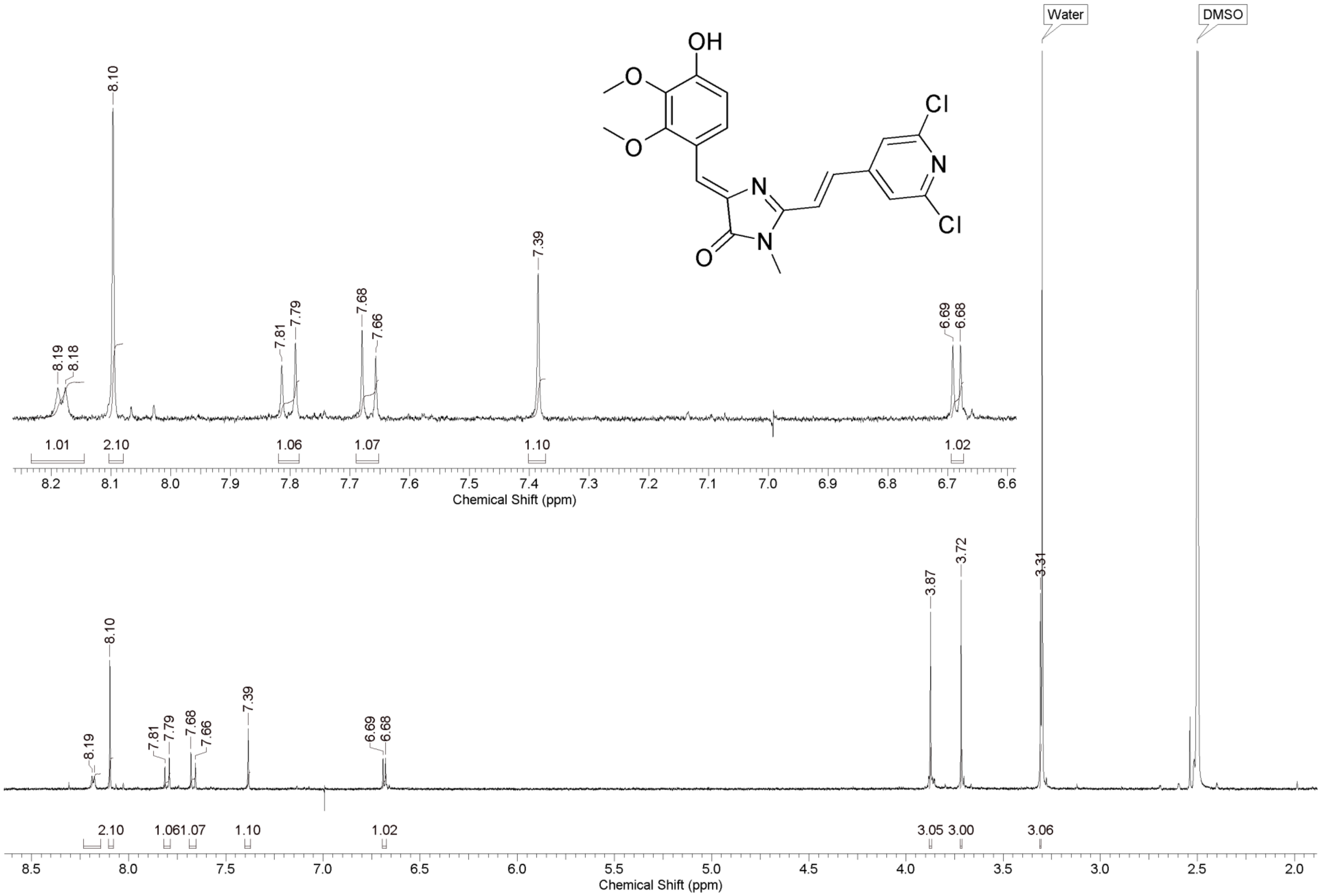

Back to Contents

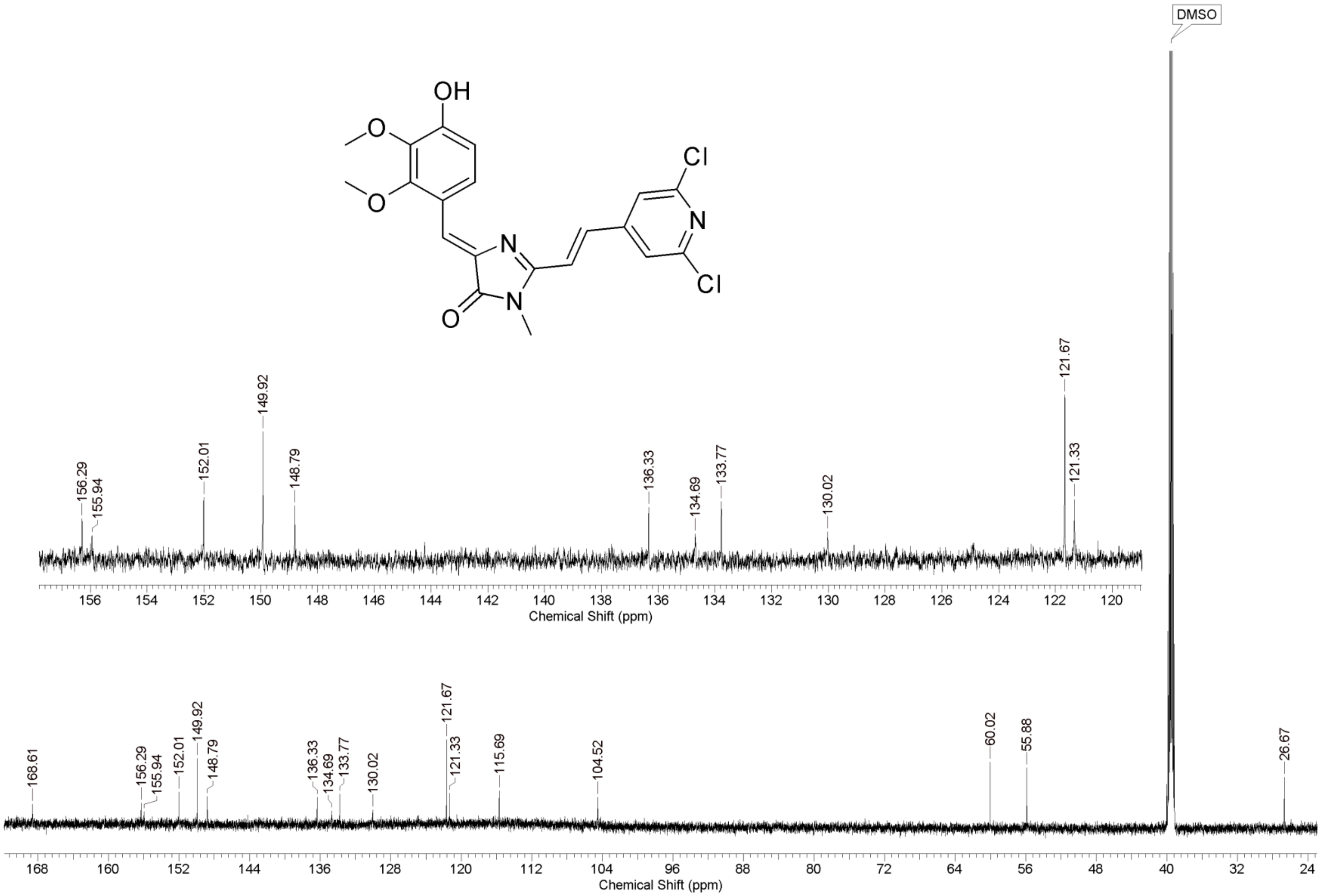

[Back to Contents](#)

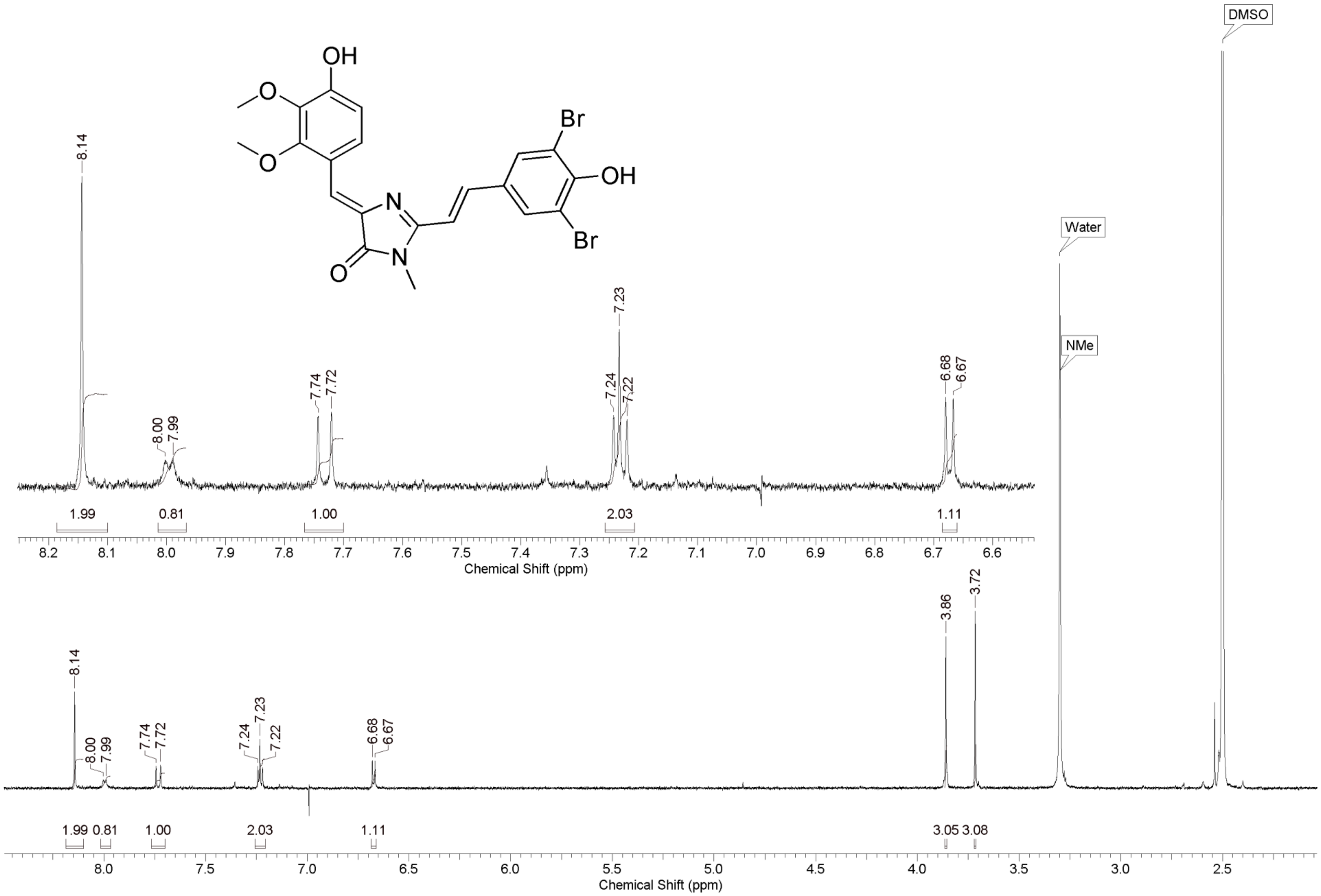

[Back to Contents]

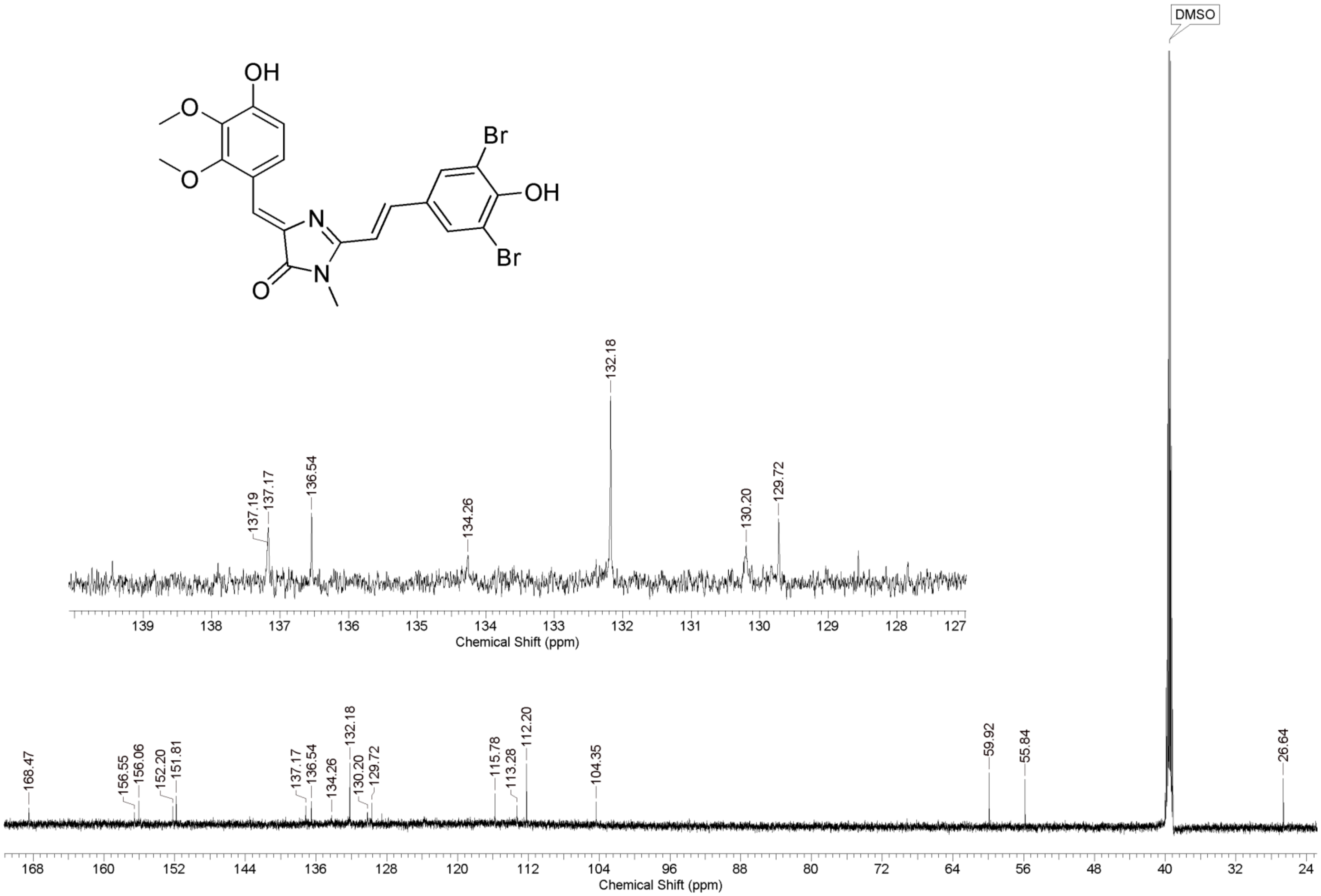

Back to Contents

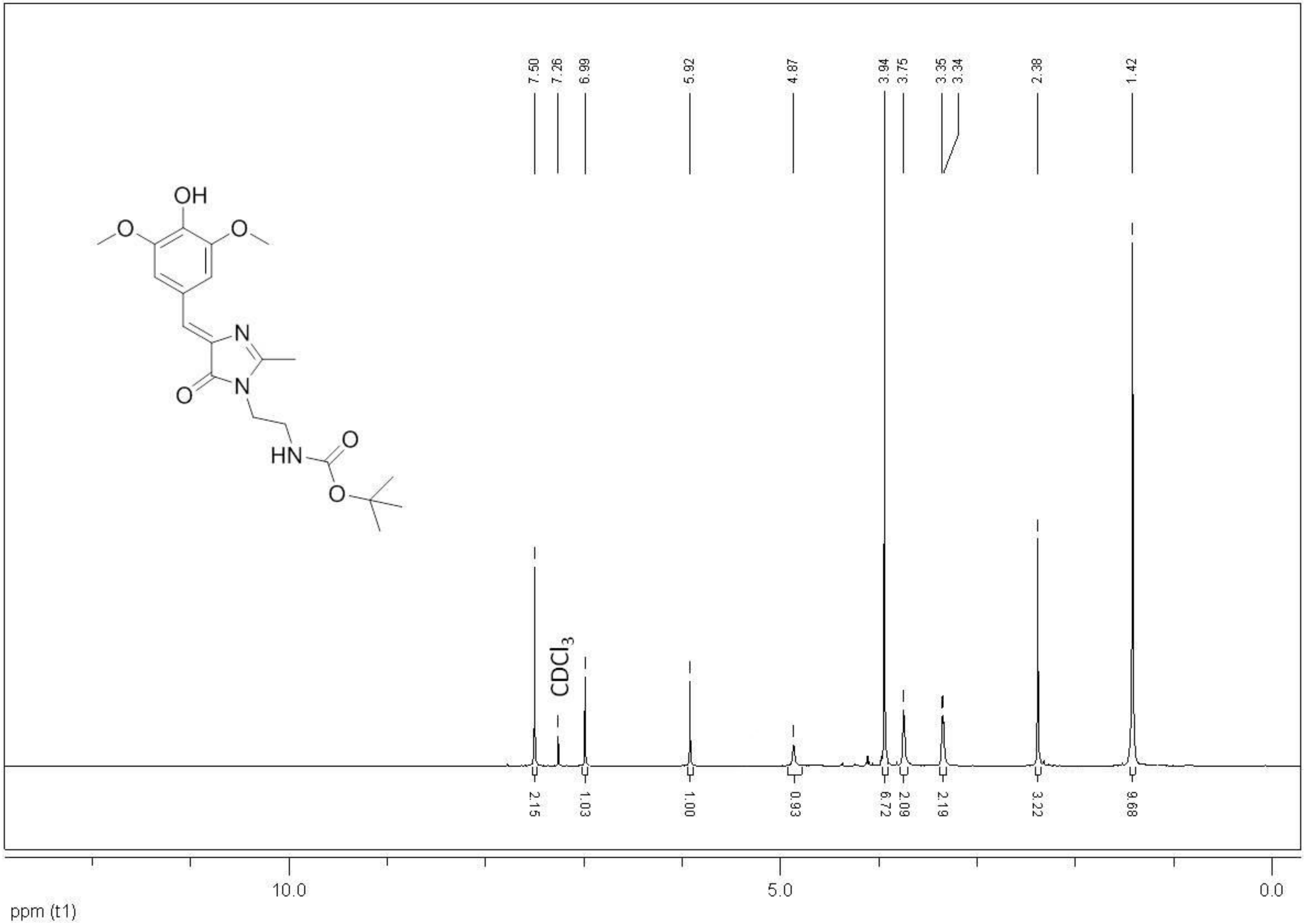

[Back to Contents]

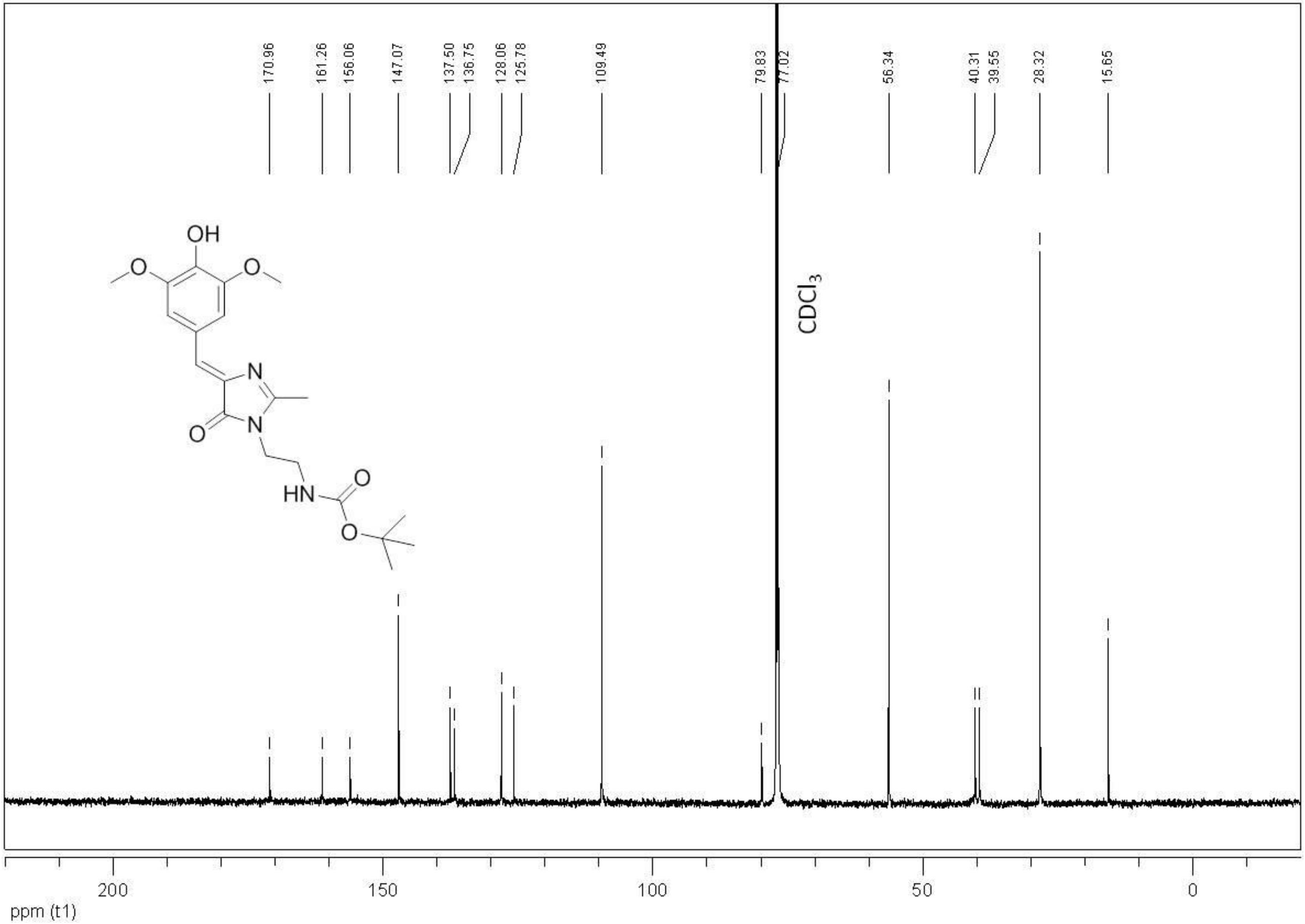

[Back to Contents]

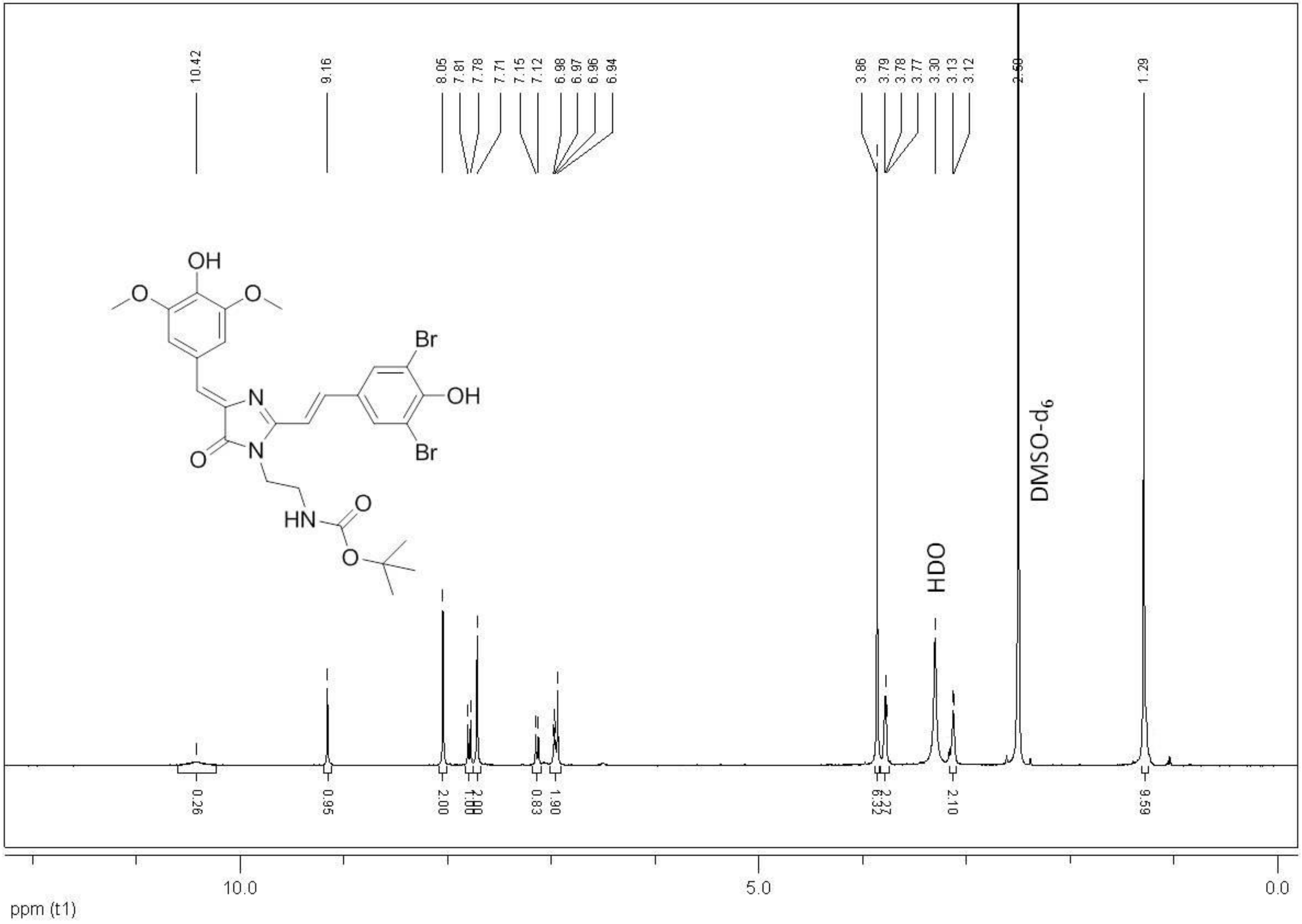

Back to Contents

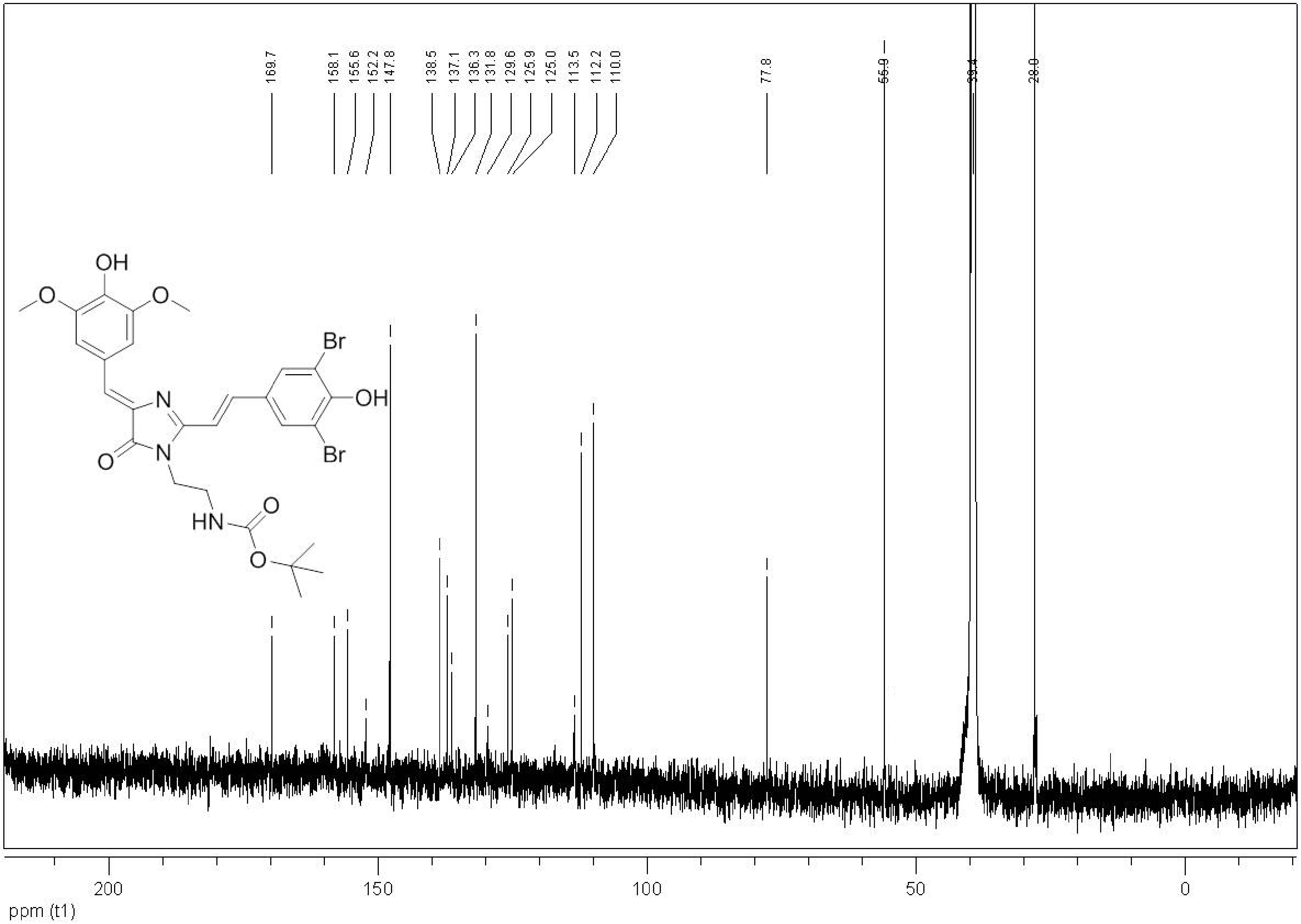

Back to Contents

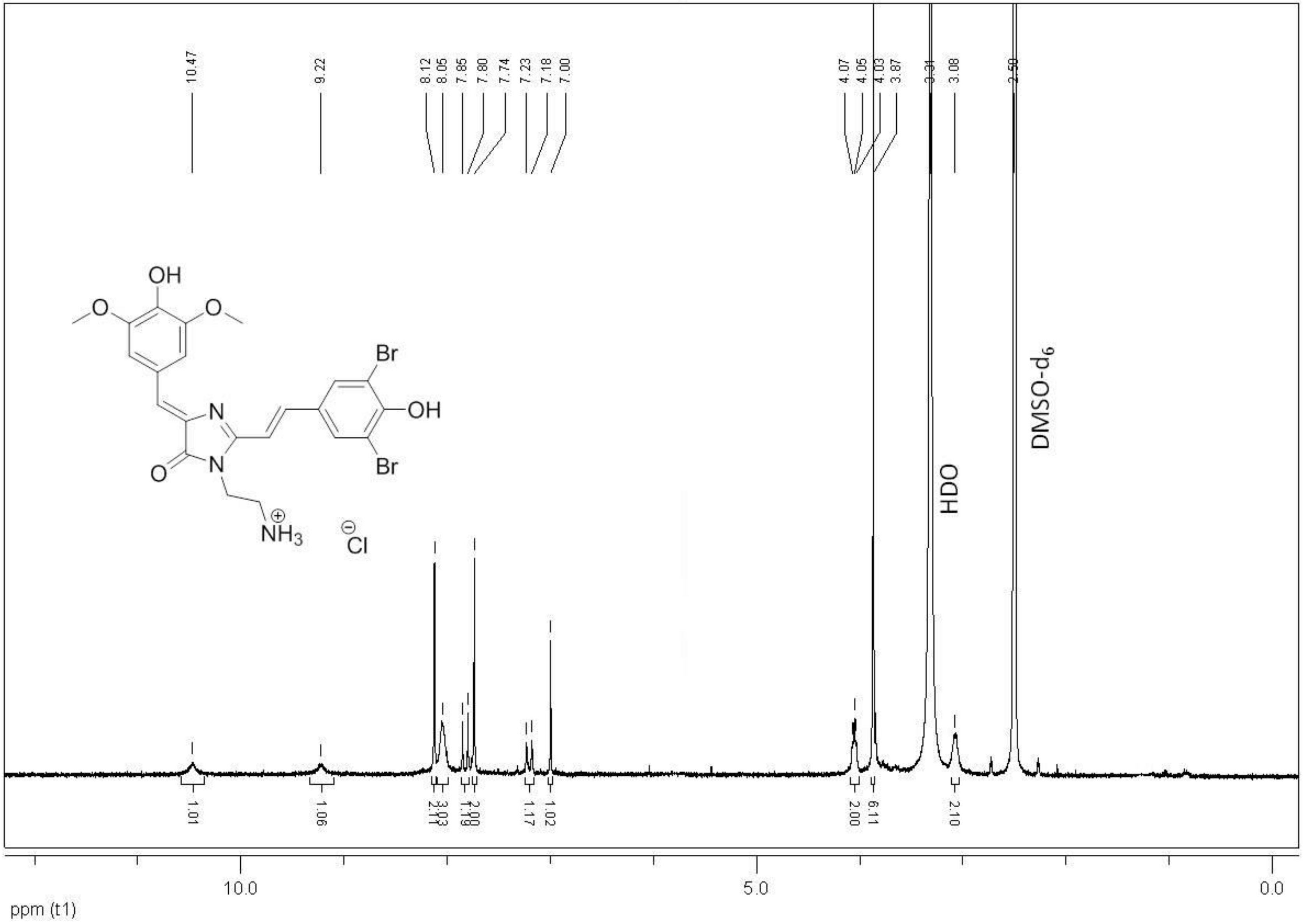

[Back to Contents](#)

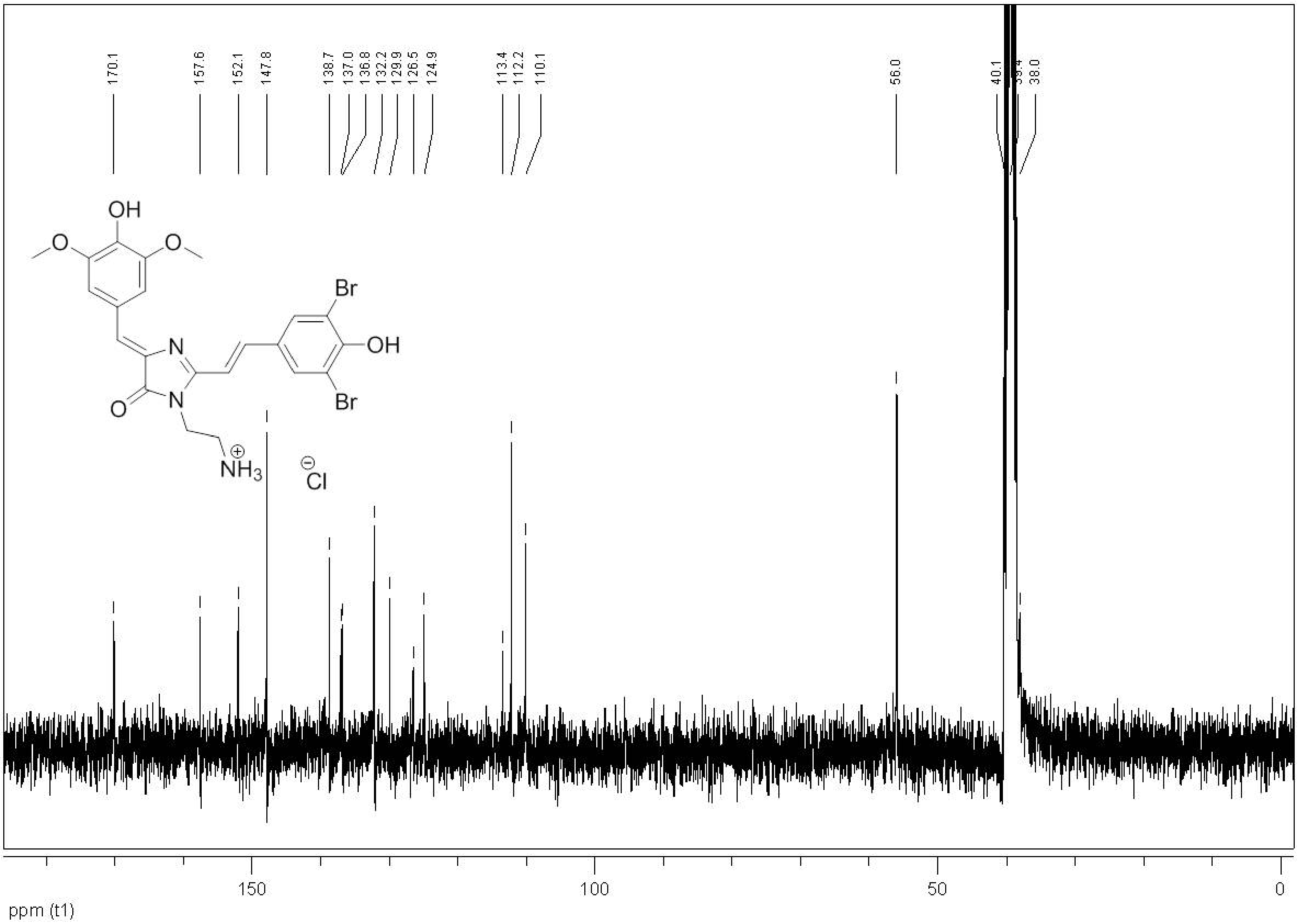

Back to Contents

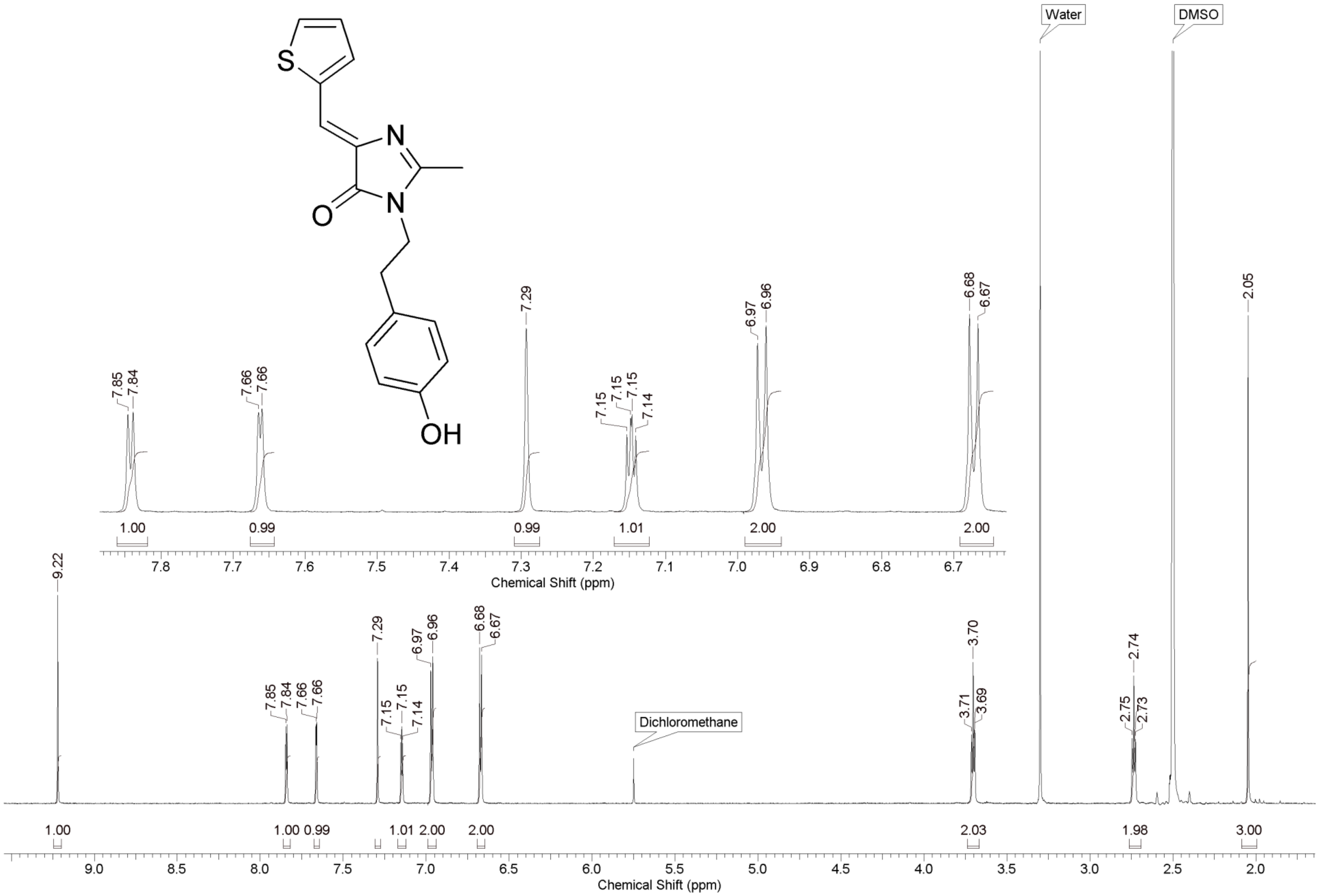

[Back to Contents](#)

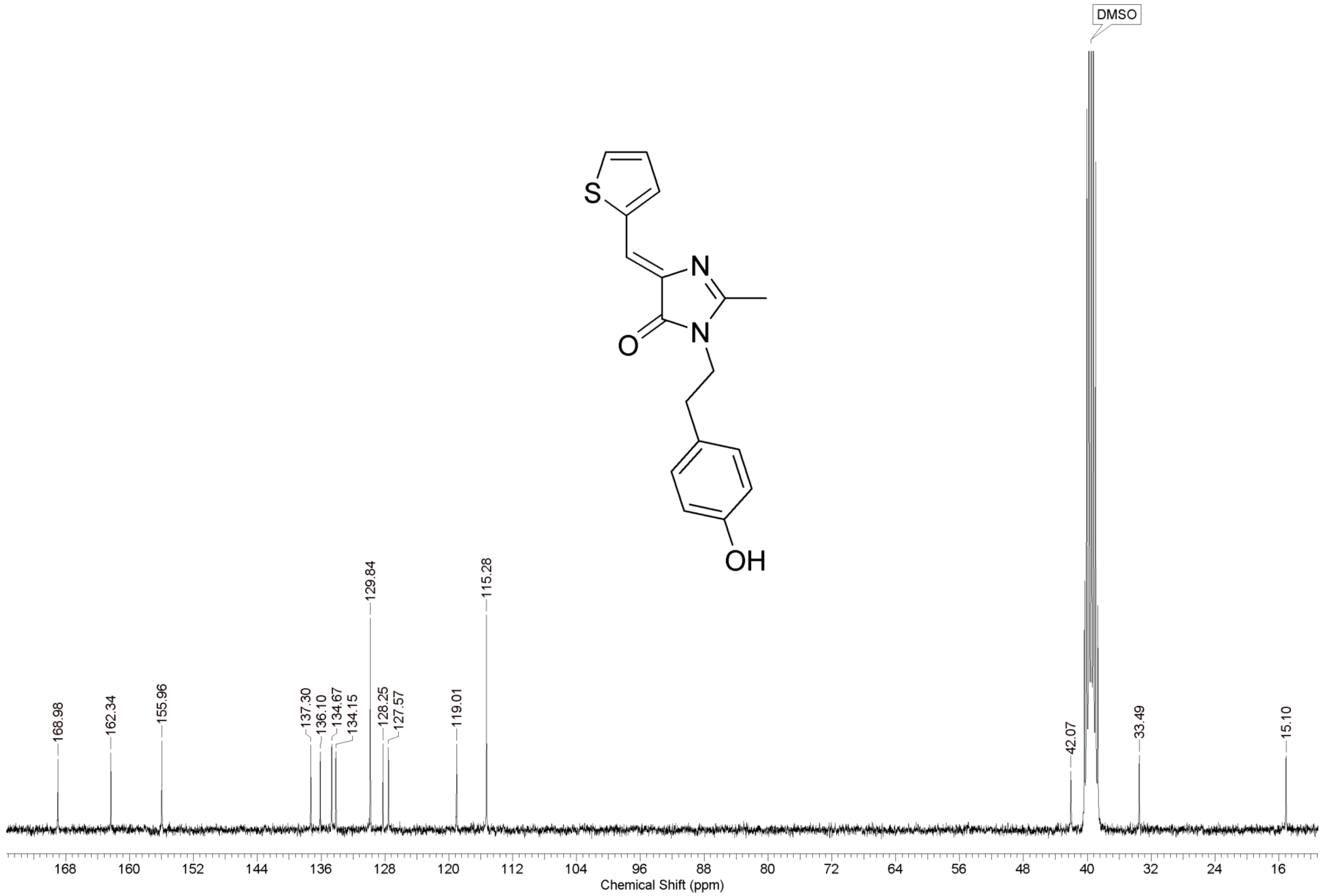

[Back to Contents](#)

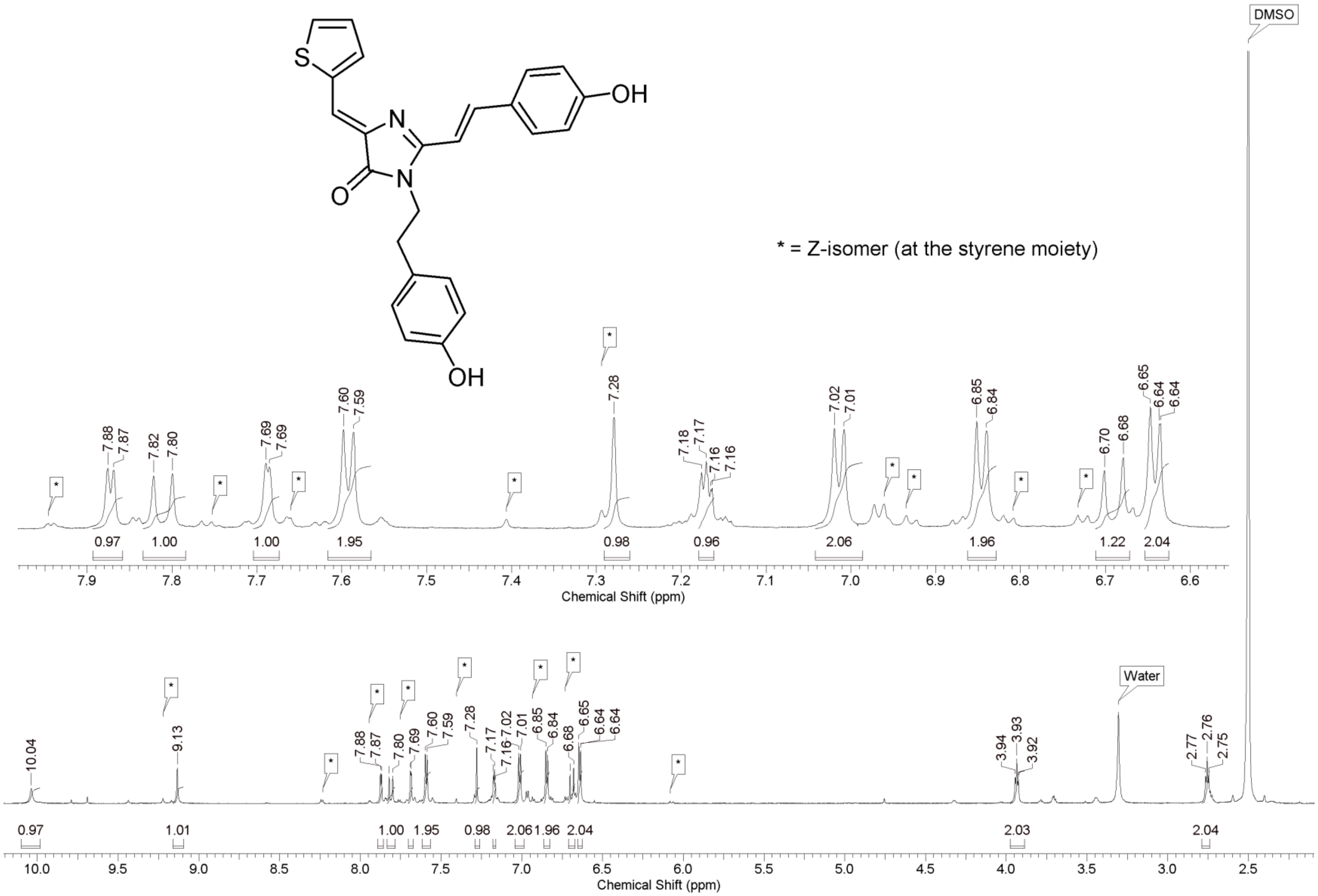

Back to Contents

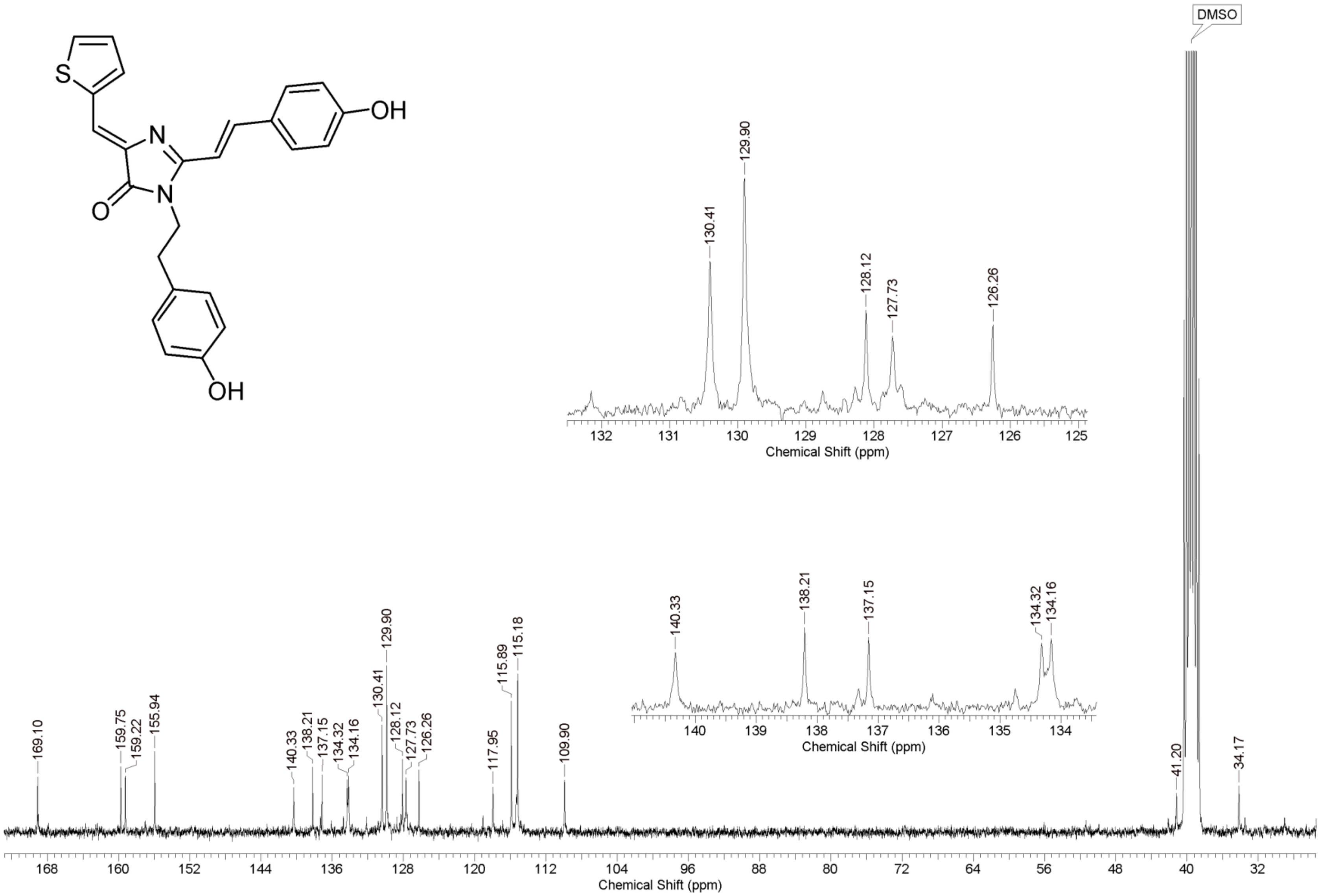

Back to Contents

Supplement S8. NMR titration data for the LTR-III/ZS260.1 complex

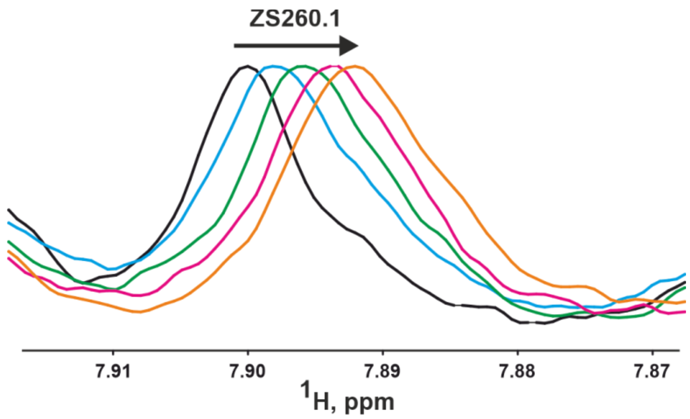

Figure S8-1. Chemical shifts changes in H8 G8 of 50 μM LTR-III upon addition of **ZS260.1** ligand at 20 °C, pH 7.0, 800 MHz.

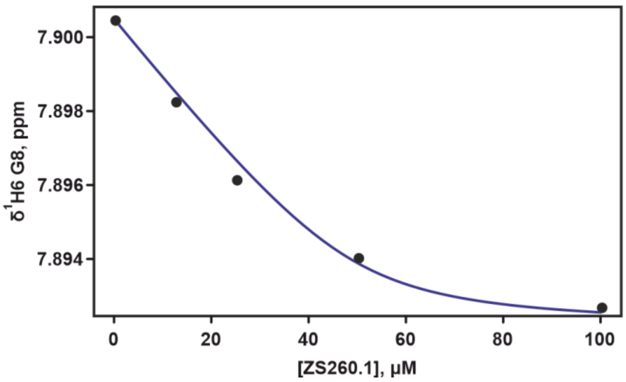

Figure S8-2. The binding curve, describing interaction of LTR-III G4 with **ZS260.1**, approximated by dissociation equation.

Back to Contents

Supplement S9. HIV LTR-III G4 MD simulation parameters

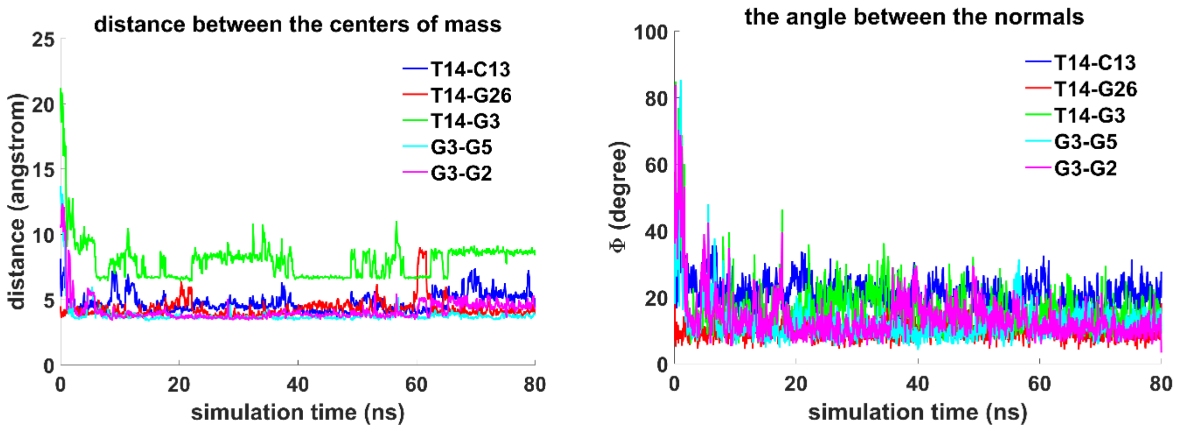

Figure S9-1. Time plots of the center of mass (COM) distances between T14, G3 and surrounding nucleobases (**A**) and of angles (Φ) between the normals to the nucleobases planes (**B**). The plots were smoothed using the moving average method (span = 5).

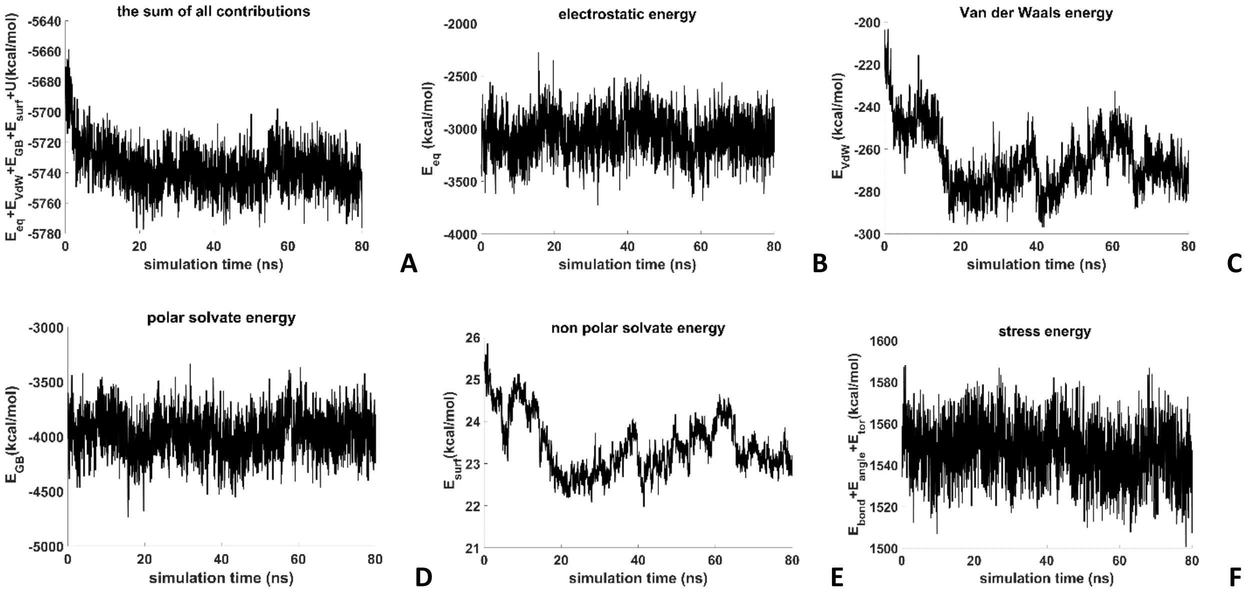

Figure S9-2. Evolution of the contributions to the free energy of the LTR-III structure. The values of parameters on the plots were smoothed using the moving average method (span = 5) . E_q – electrostatic, E_{vdw} - Van der Waals, E_{GB} - polar energy of solvation, E_{surf} - non-polar energy of solvation due to the hydrophobic surface available to the solvent, U – stress energy.

Back to Contents

Supplement S10. The conformation of the LTR-III/ZS260.1 complex (additional simulation angles)

The conformations of the complexes with **ZS260.1** within the of the LTR-III structure at the initial, 0 ns (left) and final, 80 ns (right) stages of MD simulations. The DNA shown by rendering with the nucleotides are coloured as follows: G – blue, A – red, T – light brown, C - cyan. Cavity-forming nucleotides and ligand are in ball-and-stick representation. Atoms are represented by the following colours: ligand carbon – yellow, DNA carbon – ice blue, oxygen – red, nitrogen – blue, and hydrogen – white.

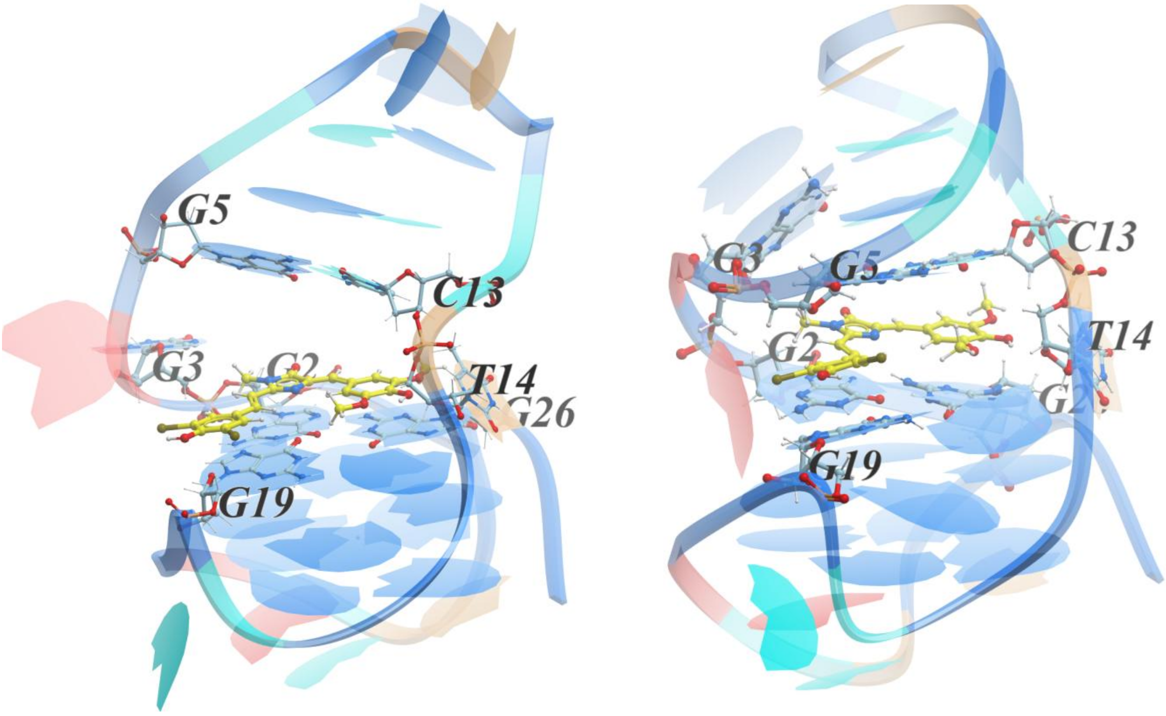

Back to Contents

Supplement S11. HIV LTR-III G4/ZS260.1 complex MD simulation parameters

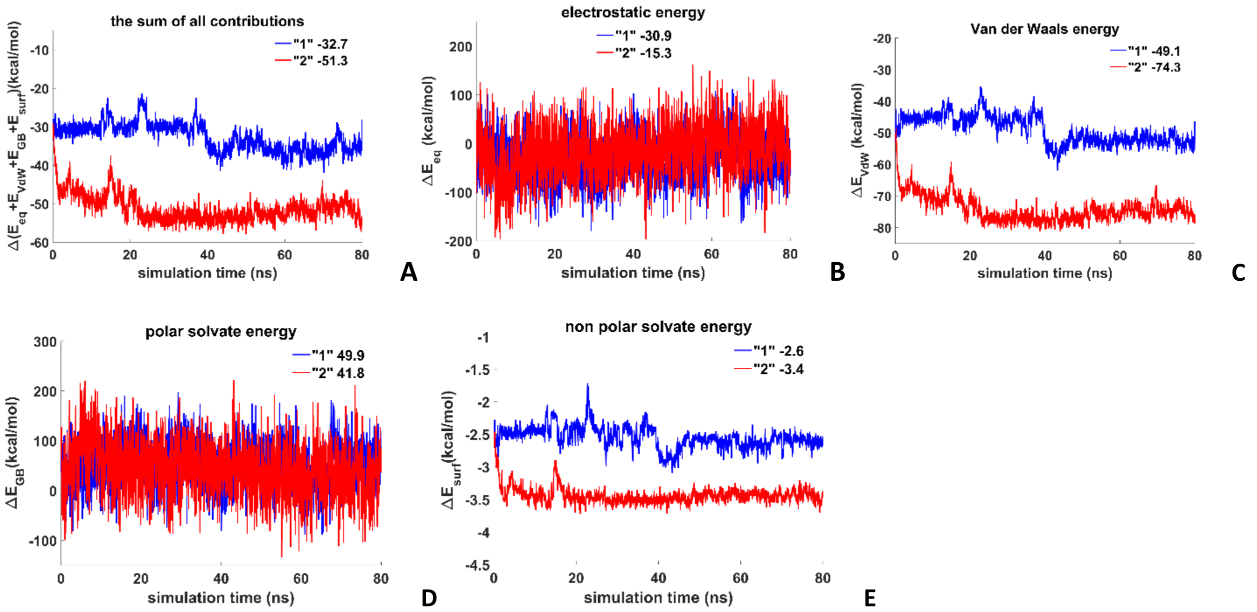

Figure S11-1. Evolution of the contributions to the interaction energy of LTR-III/ZS260.1 complexes. The plots were smoothed using the moving average method (span = 5). In the legends to the graphs, the average values are indicated. E_q – electrostatic, E_{vdw} - Van der Waals, E_{GB} - polar energy of solvation, E_{surf} - non-polar energy of solvation due to the hydrophobic surface available to the solvent.

Back to Contents

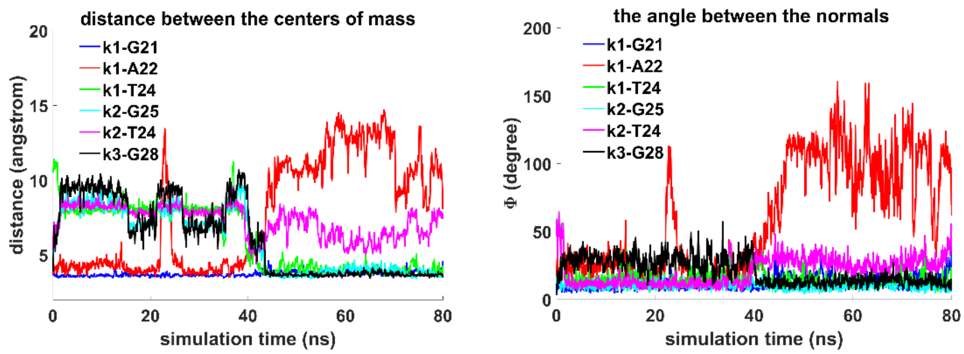

1

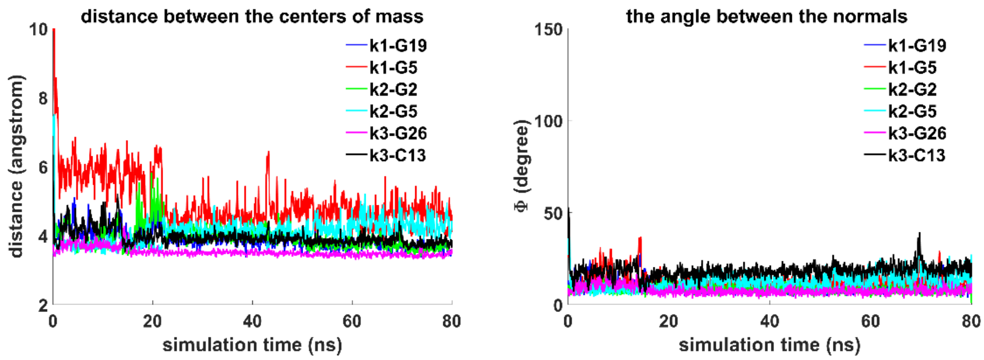

2

Figure S11-2. Time plots of the center of mass (COM) distances and angles (Φ) between the normals, characterizing the interposition of the ligand and adjacent nucleobases. K1 – COM of dibromophenyl moiety, K2 – COM of imidazolone moiety, K3 – COM of dimethoxyphenyl moiety.

Back to Contents

Table S12. Antiviral activity of the tested compounds in MT4 cells against HIV-1 (st. NL4-3).

cmpd	EC_{50}, µM	CC_{50} (MT4), µM	LTR-III (average), °C
N1193	n/a*	2.3±1.1	14
N1196	n/a	>25	11,7
N1197	n/a	1.66±0.79	11,3
ZS260	3.78±0.91	4.1±0.6	8,35
N1068.2	1.78±0.38	3.0±1.0	6,9
N1198	1.78±0.38	2.8±1.1	6,5
N960a	n/a	>25	5,2
N1052	n/a	6.6±3.1	4,5
N1199	3.3±1.1	4.6±2.5	4,3
N1195	3.78±0.91	5.9±2.6	4,2
ZS331	n/a	1.17±0.55	3,7
N848.3	n/a	5.5±2.2	3,3
N848.2	n/a	6.4±1.8	3,2
N683.1	n/a	>25	3,1
ccE4	n/a	>25	2,8
N1069	4.23±1.75	6.0±2.0	2,7
M903.2	n/a	>25	2,3
N1122	n/a	>25	2,3
N1049	2.1±1.4	5.2±2.5	1,8
M813	n/a	0.46±0.16	1,65
AR559	n/a	>25	1,2
N906	n/a	6.6±3.1	0,1
SA120	n/a	>25	-1,35
N840	n/a	>25	-1,55
MID161	7.7±2.2	17.3±5.7	-1,7
M802	n/a	6.6±3.1	-2,3
M2227	n/a	>25	-2,7
AZT	0.0071±0.00043	8.0±1.5	-

* – not active at non-toxic concentrations

Back to Contents
